# Dynamic Covalent Self‐Assembly of Chloride‐ and Ion‐Pair‐Templated Cryptates

**DOI:** 10.1002/anie.202201831

**Published:** 2022-05-12

**Authors:** Selina Hollstein, Oleksandr Shyshov, Marko Hanževački, Jie Zhao, Tamara Rudolf, Christof M. Jäger, Max von Delius

**Affiliations:** ^1^ Institute of Organic Chemistry Ulm University Albert-Einstein-Allee 11 89081 Ulm Germany; ^2^ Department of Chemical and Environmental Engineering University of Nottingham University Park Nottingham NG7 2RD UK

**Keywords:** Chloride Binding, Host–Guest Chemistry, Ion Pairs, Molecular Dynamics, Self-Assembly

## Abstract

While supramolecular hosts capable of binding and transporting anions and ion pairs are now widely available, self‐assembled architectures are still rare, even though they offer an inherent mechanism for the release of the guest ion(s). In this work, we report the dynamic covalent self‐assembly of tripodal, urea‐based anion cryptates that are held together by two orthoester bridgeheads. These hosts exhibit affinity for anions such as Cl^−^, Br^−^ or I^−^ in the moderate range that is typically advantageous for applications in membrane transport. In unprecedented experiments, we were able to dissociate the Cs⋅Cl ion pair by simultaneously assembling suitably sized orthoester hosts around the Cs^+^ and the Cl^−^ ion.

## Graphical abstract

The combination of urea‐based diols and simple orthoesters allowed the dynamic covalent encapsulation of the chloride ion and the simultaneous encapsulation of both ions present in cesium chloride.

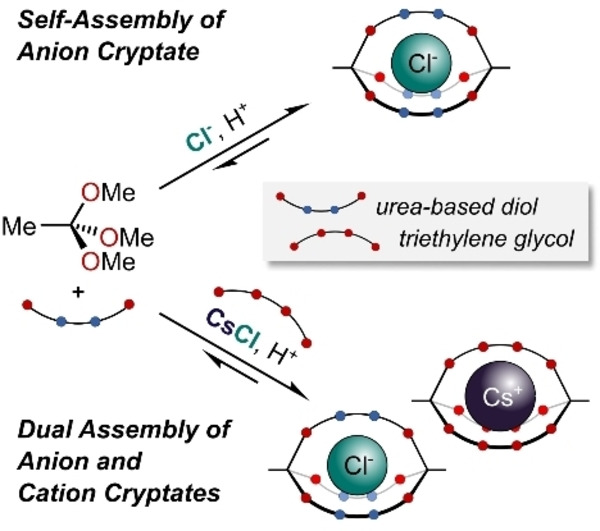

## Introduction

Half a century ago, pioneering reports by Park and Lehn^[1]^ demonstrated that simple anions such as chloride could be encapsulated within artificial receptors. Over the past two decades, progress in synthesis^[2]^ has been driven and accompanied by a growing body of research on applications of anion receptors,^[3]^ most notably in sensing,^[4]^ transport^[5]^ and for environmental^[6]^ as well as medicinal purposes.^[7]^

While a large number of conventionally synthesized anion receptors have been reported to date, with some macrobicyclic architectures featuring exceptionally high binding affinities,^[8]^ self‐assembled, purely organic^[9]^ hosts for anions are still scarce. To the best of our knowledge, self‐assembled, purely organic hosts for ion pairs are elusive. Examples for self‐assembled, organic anion receptors include hydrogen‐bonded cages (guest: PO_4_^3−^ or SO_4_^2−^),^[10]^ disulfide‐based dimers of cyclic peptides (guest: SO_4_^2−^)^[11]^ and architectures based on imines or acyl hydrazones.^[12]^ For applications where effective anion transport benefits from the stimuli‐responsive dissociation of the guest,^[13]^ it seems likely that self‐assembled hosts could play a decisive role, because they offer an inherent release mechanism.

**Figure 1:**
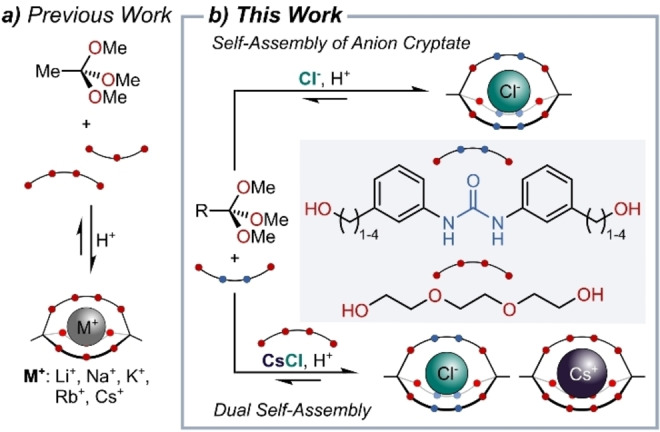
a) Previous work based on metal‐templated orthoester cryptates. b) Chloride‐templated self‐assembly of orthoester cryptates and one‐pot co‐assembly of anion and cation cryptates (R=CH_3_, H).

Given the fact that tripodal architectures are especially widespread in anion receptor chemistry,^[14]^ we wondered whether we could make use of the dynamic covalent chemistry^[15]^ of orthoesters,^[16]^ which we had previously employed to self‐assemble cryptates for simple cations (Figure 1a).^[17]^ Such orthoester cryptands degrade in aqueous solvent with tunable rate constants and therefore offer a pH‐driven release mechanism^[18]^ that could be of use in the context of supramolecular medicinal chemistry.[^7a^, ^7b^, ^19^] Herein, we report the dynamic covalent self‐assembly of such hosts based on the chloride‐templated reaction of simple orthoesters with urea‐based diols (Figure 1b, top). While their poor solubility precluded membrane transport experiments with this first generation of cryptands, we were able to use Cs⋅Cl as a dual template and simultaneously self‐assemble two different orthoester hosts to encapsulate the Cs^+^ cation and the Cl^−^ anion (Figure 1b, bottom).

## Results and Discussion

### Chloride‐Templated Self‐Assembly of Cryptates

When designing building blocks for self‐assembled anion‐binding architectures, the choice of anion‐binding motif is of paramount importance. We chose (di)phenylurea, because an abundance of non‐macrocyclic, tripodal anion receptors of this type are reported.^[14]^ We therefore synthesized bishydroxyalkylphenyl urea compounds with varying alkyl chain length (**1**–**3**) that should act as linkers between two orthoester bridgeheads in the envisaged cryptates. For orthoester‐based self‐assembly reactions, we chose an acid catalyst whose conjugate base would bind to urea only weakly, a mixed solvent system that represents a good compromise between solubility and (non)interference with hydrogen bonding and a chloride salt with a weakly binding counterion. Addition of molecular sieves (5 Å) helps keeping the reaction medium anhydrous and shifts the equilibrium to the product side by adsorption of methanol.[^16a^, ^17^, ^18^]

**Figure 2:**
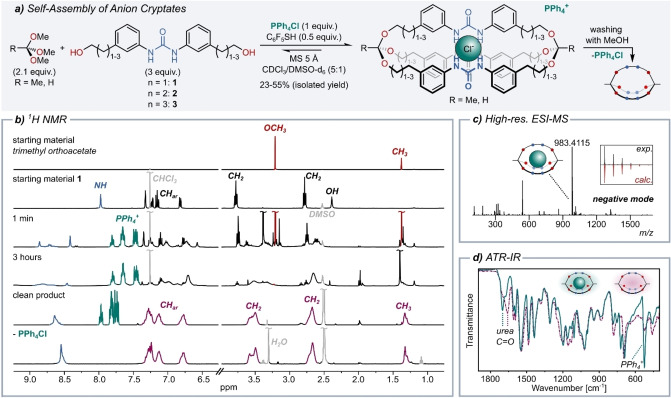
a) Chloride‐templated synthesis of orthoester/urea cryptates. Reaction conditions: 30 μmol tetraphenylphosphonium chloride, 63 μmol trimethyl orthoacetate or trimethyl orthoformate, 90 μmol diol **1**, **2** or **3** and 15 μmol 2,3,4,5,6‐pentafluorothiophenol in CDCl_3_/deuterated dimethyl sulfoxide (DMSO‐d_6_) mixture (5 : 1, 3.3 mL); MS: molecular sieves. Reaction time: 3 hours (*n*=1), 18 hours (*n*=2, 3). b) ^1^H‐NMR stacked plot for the synthesis and purification of ***o*** **‐Me_2_‐ur‐C2**. Spectra of starting materials and reaction control were recorded in CDCl_3_ and spectra of cryptate and cryptand were recorded in DMSO‐d_6_. c) High‐resolution electron spray mass spectrometry in negative mode of **[Cl** ^−^ ⊂ ***o*** **‐Me_2_‐ur‐C2]PPh_4_** ^+^. Minor peaks originate from solvents or hydrolysis products. Inset: experimental and calculated isotopic distribution of product peak. d) Attenuated total reflection‐infrared (ATR‐IR) spectra of **[Cl** ^−^ ⊂ ***o*** **‐Me_2_‐ur‐C2]PPh_4_** ^+^ (teal) and ***o*** **‐Me_2_‐ur‐C2** (purple).

In a typical self‐assembly experiment, we therefore treated a solution of diol **1** with trimethyl orthoacetate, a catalytic amount of acid 2,3,4,5,6‐pentafluorothiophenol, and a stoichiometric amount of the template tetraphenylphosphonium chloride (Figure 2a). The reaction was monitored by ^1^H‐NMR spectroscopy (Figure 2b), clearly indicating efficient orthoester exchange, because after only three hours the signal belonging to the methoxy groups of the orthoester had fully disappeared. Interestingly, the time required for complete methoxy group (and MeOH) displacement differed substantially between diols **1**, **2** and **3** (3 h, 18 h and 18 h, respectively), raising the question whether this could be due to a difference in Cl^−^ binding affinity.

After quenching the self‐assembly reactions by adding excess triethylamine, precipitation from diethyl ether furnished the pure products **[Cl** ^−^ ⊂ ***o*** **‐Me_2_‐ur‐C2]PPh_4_** ^+^, **[Cl** ^−^ ⊂ ***o*** **‐Me_2_‐ur‐C3]PPh_4_** ^+^, **[Cl** ^−^ ⊂ ***o*** **‐Me_2_‐ur‐C4]PPh_4_** ^+^ and **[Cl** ^−^ ⊂ ***o*** **‐H_2_‐ur‐C4]PPh_4_** ^+^ in isolated yields of 55%, 48%, 23% and 44%, respectively. The formation of the chloride‐binding cryptates was corroborated by ^1^H‐NMR and ^13^C‐NMR spectroscopy (Figure 2b and Figure S2), high‐resolution mass spectrometry (Figure 2c) and attenuated total reflection‐infrared spectroscopy (Figure 2d). The representative ^1^H‐NMR spectrum of **[Cl** ^−^ ⊂ ***o*** **‐Me_2_‐ur‐C2]PPh_4_** ^+^ is indicative of a pure compound, but exhibits significant peak broadening, which also has been found in a related thiourea host.^[20]^

### Molecular Dynamics: Structure and H‐Bonding

**Figure 3:**
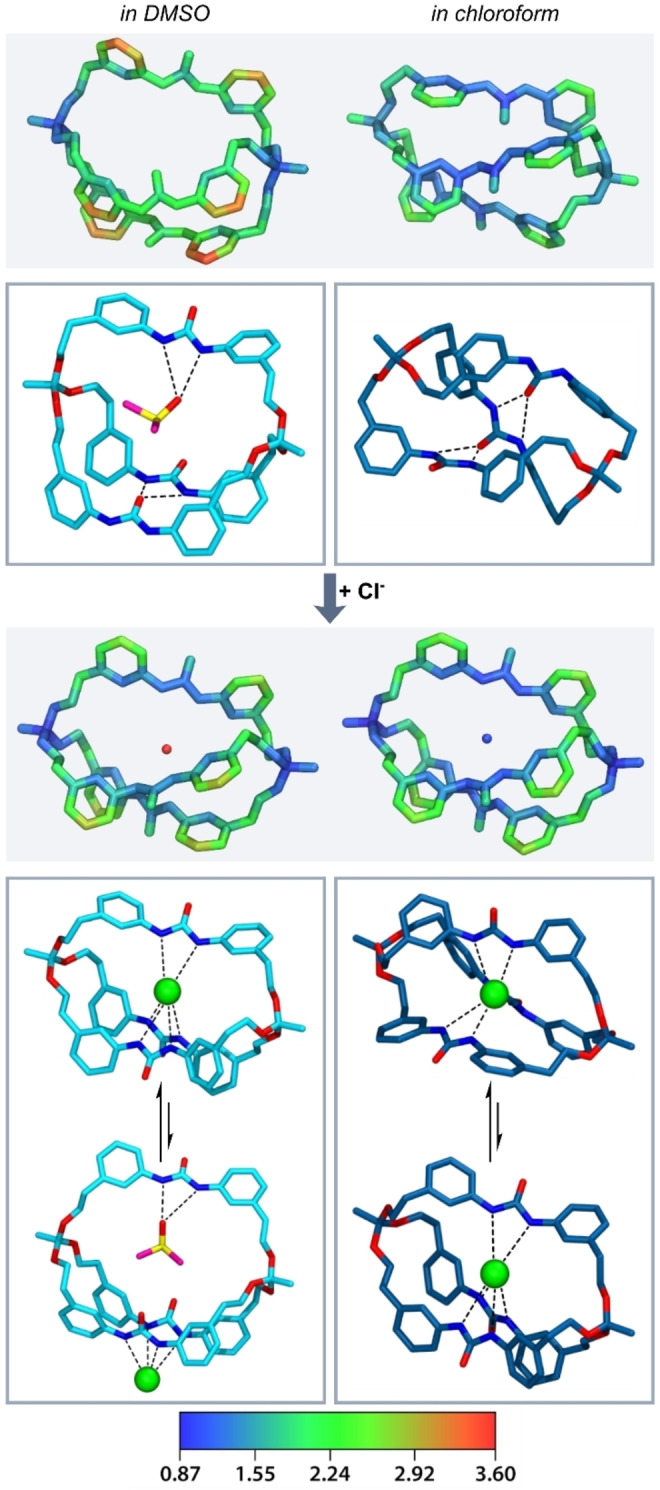
Dynamic structural analysis of ***o*** **‐Me_2_‐ur‐C2** from MD simulations. Flexibility of cages indicated by atomic root mean square fluctuations (rmsf) of heavy atoms applied as color scale on centroid structures. Color scale values presented in Å. Representative conformations in DMSO (left) and chloroform (right) without (top) and with (bottom) chloride ion binding demonstrate the possibility of DMSO molecules binding inside the cage with and without presence of the anion, whilst structures without chloride in chloroform demonstrate frequent intramolecular hydrogen bonding between urea units.

To gain insights into the dynamic structure of cryptands and cryptates, we carried out microsecond molecular‐dynamics (MD) simulations. These confirm a very high flexibility of the cryptate systems that is significantly influenced by the solvent composition. Simulations were carried out for ***o*** **‐Me_2_‐ur‐C2** in the presence and absence of Cl^−^ in pure chloroform or DMSO as well as in a 5 : 1 solvent mixture. In DMSO, the cryptand demonstrates high flexibility with the possibility for DMSO molecules to enter the cryptand and form hydrogen bonds with urea NH groups. In the apolar solvent chloroform on the other hand, ***o*** **‐Me_2_‐ur‐C2** demonstrates a slightly more compact structure, with hydrogen bonds frequently formed between the urea functionalities (Figure 3 and Figure S50).

In complex with Cl^−^, the predominant structure is such that all three urea entities bind to the encapsulated anion. Still, the flexibility of the cryptate framework remains high, as shown in Figure 3 (and Figure S51). Higher fluctuations were observed in the phenyl containing linkers, while orthoester bridgeheads and urea groups remained more static. The phenyl linkers demonstrate varying interactions, including frequent but transient π‐stacking interactions between linkers.

Importantly, an additional binding geometry can be found in longer simulations when DMSO is present. Here again, DMSO can enter the cavity, forming hydrogen bonds to urea, leaving the chloride ion only two further urea units to bind. This alternative binding mode, although less frequent, also demonstrated a lifetime of up to 500 ns in simulations in 5 : 1 (chloroform/DMSO) solvent (Figure S52c).

Thus, with DMSO present two different, slowly exchanging binding modes can exist (see Figure 3) where the Cl^−^ ion either binds all three or only two urea units. A similar change in the complexation of Cl^−^ by ***o*** **‐Me_2_‐ur‐C2** has not been observed in any of the performed simulations in chloroform underlining this important solvent effect. The combination of high conformational flexibility, with overall atomic fluctuations up to remarkable six times higher compared to the cation binding cryptate systems investigated before,^[21]^ asymmetric Cl^−^ coordination, and competitive DMSO inclusion could therefore explain the broadened peaks in the ^1^H‐NMR spectra as well as the moderate experimental binding affinities (see below). The dynamic binding mode may also be the reason why we were unable to obtain single crystals for X‐ray crystallography, despite extensive efforts.

### Host–Guest Titrations and MS/MS Studies of Heteroleptic Cryptands

To achieve the removal of the chloride template, we treated the solid cryptate **[Cl** ^−^ ⊂ ***o*** **‐Me_2_‐ur‐C2]PPh_4_** ^+^ with anhydrous methanol, in the hope that this would lead to the selective dissolution of the tetraphenylphosphonium chloride. After stirring the suspension for one day, the empty cryptand was obtained as indicated by the missing PPh_4_^+^ signals in the ^1^H‐NMR spectrum (Figure 2b, bottom). As expected, the signal of the proton belonging to the urea moiety shifted upfield upon removal of the chloride ion, but not as much as in diol **1**, which could be an additional indication for a (dynamic) competition between chloride binding and intramolecular hydrogen bonding. Evidence for hydrogen bonding was obtained by ATR‐IR measurements. When comparing the spectra of the cryptate and cryptand, the expected shift of the urea carbonyl band to lower wave number (from 1700 to 1660 cm^−1^)^[22]^ is observed (Figure 2d, exemplary for **[Cl** ^−^ ⊂ ***o*** **‐Me_2_‐ur‐C2]PPh_4_** ^+^).

With the empty cryptands in hand, we were able to determine the binding constants to different anions, namely chloride, bromide, iodide and nitrate (with counterion PPh_4_^+^ or NMe_4_^+^). The use of fluoride was ruled out as addition of fluoride would lead to deprotonation of the urea moieties.^[23]^ We performed the titration of ***o*** **‐Me_2_‐ur‐C2** with chloride in the solvent mixture chloroform/DMSO (5 : 1) to enable a direct comparison between association constants and self‐assembly reactions. We obtained an association constant (*K*_a_) of 286±23 M^−1^. At the concentration used in the self‐assembly reactions (9 mM) this binding constant implies that ca. 55% of the complex is formed (according to Bindsim on supramolecular.org^[24]^), which correlates with the observed isolated yield of 55% for **[Cl** ^−^ ⊂ ***o*** **‐Me_2_‐ur‐C2]PPh_4_** ^+^.

For solubility reasons, comparative titrations for all cryptands and different anions were performed in pure DMSO, even though this solvent is highly competitive due to its high dielectric constant (*ϵ*=47^[25]^). This should be kept in mind, when comparing the *K*_a_ values reported here with related work in the field (often carried out in less competitive medium). When considering the binding constants observed for the different halide ions for host ***o*** **‐Me_2_‐ur‐C2**, a moderate selectivity for chloride can be inferred (Table 1). The *K*_a_ values determined for ***o*** **‐Me_2_‐ur‐C2** and its larger analogues ***o*** **‐Me_2_‐ur‐C3** and ***o*** **‐Me_2_‐ur‐C4** are decreasing with ascending number of methylene groups, which could reflect the best size fit for ***o*** **‐Me_2_‐ur‐C2** and the chloride ion and/or the entropic cost derived from extending the aliphatic spacer towards the orthoester bridgehead.

**Table 1:** Binding affinities of orthoester cryptand ***o*** **‐Me_2_‐ur‐C2** to Cl^−^, Br^−^_,_ I^−^ and NO_3_^−^ salts and cryptands ***o*** **‐Me_2_‐ur‐C3**, ***o*** **‐Me_2_‐ur‐C4** and ***o*** **‐H_2_‐ur‐C4** to Cl^−^ salt (*K*_a_ values in CDCl_3_/DMSO‐d_6_ (5 : 1) or DMSO‐d_6_ containing up to 10% water at 298 K).

Host	*K*_a_ [M^−1^] for Guests			
	Cl^−[a]^	Br^−[a]^	I^−[a]^	NO_3_^−[a]^
***o*** **‐Me_2_‐ur‐C2** (in 5 : 1 CDCl_3_/DMSO‐d_6_)	286±23^[d]^	n.d.	n.d.	n.d.
***o*** **‐Me_2_‐ur‐C2** (in DMSO‐d_6_)	37±9^[c]^	16±1^[d]^	10±1^[d]^	8.6±0.3^[d]^
***o*** **‐Me_2_‐ur‐C3** (in DMSO‐d_6_)	27±1^[d]^	n.d.	n.d.	n.d.
***o*** **‐Me_2_‐ur‐C4** (in DMSO‐d_6_)	17±1^[d]^	n.d.	n.d.	n.d.
***o*** **‐H_2_‐ur‐C4** (in DMSO‐d_6_)	32.0±0.4^[d]^	n.d.	n.d.	n.d.

[a] Added as tetraphenylphosphonium salt. [b] Added as tetramethylammonium salt. [c] *K*_a_ determined in triplicate; this error corresponds to 95% confidence interval. [d] Error corresponds to goodness of fit produced by Bindfit on supramolecular.org.^[24]^ n.d.=not determined.

To leverage the orthoester bridgehead for increasing the binding strength, we self‐assembled cryptand ***o*** **‐H_2_‐ur‐C4**, which is based on orthoformate (R=H) in which additional hydrogen bonding could occur between the anion and an inverted bridgehead.[^21b^, ^26^] The fact that we observed a moderately higher binding affinity for this host, yet no shift of the orthoformate ^1^H‐NMR signal during titration with TPPCl, suggests that this small difference (factor 2) is due to structural differences between orthoacetate and orthoformate cages^[18]^ rather than the *in*/*in* binding we had originally hoped for. In any case, competition with intramolecular hydrogen bonding^[27]^ (and the competitive solvent) prevents even higher binding constants. In this context, it should be kept in mind that moderate binding constants are often sufficient or even beneficial for transmembrane anion transport.[^27^, ^28^]

**Figure 4:**
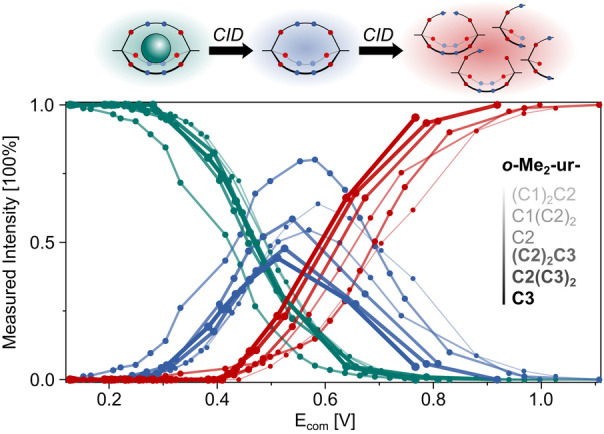
Ratio of cryptate, cryptand and products of degradation depending of applied voltage obtained by electrospray‐ionisation collision‐induced dissociation mass spectroscopy (ESI‐CID‐MS) measurements. After isolation of the product peak, increasing the voltage leads to release of the chloride ion (decrease of cryptate, green curve), formation of cryptand (blue curve) and subsequently, cryptand degradation (red curve). Ratio determined by relative intensities of corresponding mass peaks. Collision energy is given as center‐of‐mass frame (*E*_com_) in dependence on masses of collision gas (N_2_) and precursor ion. The lines are shown to guide the eye.

To identify the smallest possible size of cryptate for a chloride ion, we synthesized a diol containing a C1 chain (**S9**) in addition to diols **1**, **2** and **3**. Using our established self‐assembly conditions and C1 diol **S9**, we were unable to observe the formation of the corresponding chloride cryptate. Interestingly, when using a mixture of **S9** and **1**, the formation of two cryptates with different sizes (**[Cl** ^−^ ⊂ ***o*** **‐Me_2_‐ur‐(C1)_2_C2]PPh_4_** ^+^, **[Cl** ^−^ ⊂ ***o*** **‐Me_2_‐ur‐C1(C2)_2_]PPh_4_** ^+^) was observed in high resolution ESI‐MS measurements. We deduce that the smallest possible cryptate capable of strongly binding a chloride template incorporates two short **C1** diols and one longer **C2** diol (**[Cl** ^−^ ⊂ ***o*** **‐Me_2_‐ur‐(C1)_2_C2]PPh_4_** ^+^). When we used a mixture of **1** and **2**, all four possible cryptates (**[Cl** ^−^ ⊂ ***o*** **‐Me_2_‐ur‐C2]PPh_4_** ^+^, **[Cl** ^−^ ⊂ ***o*** **‐Me_2_‐ur‐(C2)_2_C3]PPh_4_** ^+^, **[Cl** ^−^ ⊂ ***o*** **‐Me_2_‐ur‐C2(C3)_2_]PPh_4_** ^+^, **[Cl** ^−^ ⊂ ***o*** **‐Me_2_‐ur‐C3]PPh_4_** ^+^) could be detected (Table S4). Tandem mass spectrometry (ESI‐MS/MS)^[29]^ allowed us to investigate the stability of such heteroleptic cryptands and their chloride complexes without having to isolate them from mixtures. In the gas‐phase, each precursor ion of the six cryptates was therefore subjected to increasing collision energies which first results in the release of the chloride ion (decrease of cryptate, green curve in Figure 4) and increase of cryptand (blue curve in Figure 4), followed by cryptand degradation via bond breakage (red curve in Figure 4; first urea then orthoester).

### Dual Self‐Assembly of Anion and Cation Cryptates

**Figure 5:**
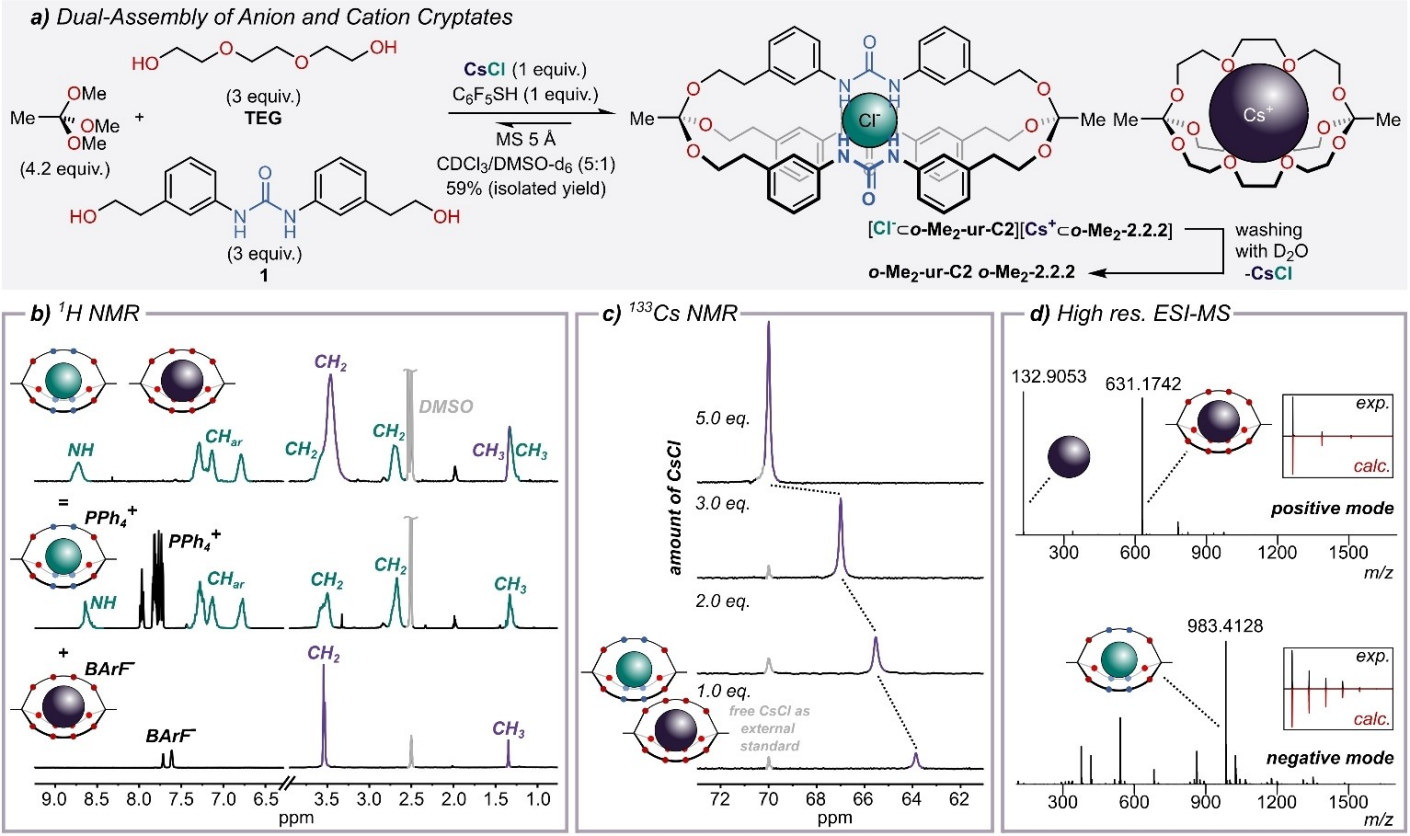
a) Cesium chloride‐templated co‐assembly of two orthoester cryptates. Reaction conditions: 45 μmol cesium chloride, 189 μmol trimethyl orthoacetate, 135 μmol triethylene glycol, 135 μmol diol **1** and 45 μmol 2,3,4,5,6‐pentafluorothiophenol in CDCl_3_/DMSO‐d_6_ mixture (MS: molecular sieves). Reaction time: 18 hours. b) Stacked plot of product after precipitation with diethyl ether (top), **[Cl** ^−^ ⊂ ***o*** **‐Me_2_‐ur‐C2]PPh_4_** ^+^ (middle) and **[Cs** ^+^ ⊂ ***o*** **‐Me_2_‐2.2.2]BArF** ^−^ (bottom). c) ^133^Cs‐NMR spectra of **[Cl** ^−^ ⊂ ***o*** **‐Me_2_‐ur‐C2][Cs** ^+^ ⊂ ***o*** **‐Me_2_‐2.2.2]** and addition of up to five equivalents CsCl. d) High‐resolution electron spray mass spectra. Top: positive mode. Main peaks belong to Cs^+^ ion and **[Cs** ^+^ ⊂ ***o*** **‐Me_2_‐2.2.2]**. Inset: top: experimental and bottom: calculated isotopic pattern of **[Cs** ^+^ ⊂ ***o*** **‐Me_2_‐2.2.2]**. Bottom: negative mode. Main peak belongs to **[Cl** ^−^ ⊂ ***o*** **‐Me_2_‐ur‐C2]**. Inset: top: experimental and bottom: calculated isotopic pattern of **[Cl** ^−^ ⊂ ***o*** **‐Me_2_‐ur‐C2]**.

Having isolated and investigated these chloride‐templated cryptands, we wondered whether CsCl could be used as an “ion pair template” in experiments aiming to self‐assemble hosts for the cation and anion simultaneously using the same dynamic covalent exchange reaction. To this end, we treated a chloroform/dimethyl sulfoxide (5 : 1) solution of diol **1** and triethylene glycol (TEG) with trimethyl orthoacetate, CsCl and 2,3,4,5,6‐pentafluorothiophenol as mild acid catalyst (Figure 5a). After only 18 hours, ^1^H‐NMR spectroscopy revealed the complete consumption of orthoesters, along with signals indicative of the expected orthoester cryptates. Similar to the aforementioned procedure, the pure product was obtained in good yield (59%) after quenching of the exchange reaction with triethylamine, followed by precipitation with diethyl ether. As expected, the ^1^H‐NMR spectrum of this material resembles the sum of spectra for the individual cryptates **[Cl** ^−^ ⊂ ***o*** **‐Me_2_‐ur‐C2]PPh_4_** ^+^ and **[Cs** ^+^ ⊂ ***o*** **‐Me_2_‐2.2.2]BArF** ^−^ (Figure 5b). The same “additive spectrum” is observed by ATR‐IR (Figures S38 and S39).

To investigate the association of the hosts to the two guest ions, additional amounts of CsCl were added and both ^1^H‐ and ^133^Cs‐NMR spectra were recorded. The ^1^H‐NMR spectra show a pronounced shift of the signal corresponding to the urea moiety, whereas the other signals are not affected (Figure S40a). We explain this finding by DMSO competing more effectively for Cs^+^ than for Cl^−^ binding, which is in agreement with previous observations.^[30]^ Nevertheless, a ^133^Cs‐NMR^[31]^ titration experiment clearly shows a shift of the signal towards the reference (CsCl as external standard in DMSO‐d_6_), confirming that the Cs^+^ ion is bound, albeit weakly (Figure 5c). The identity of the sample was further confirmed by high resolution ESI‐MS measurements. In the positive mode, only the positively charged part of the ion pair receptor **[Cs** ^+^ ⊂ ***o*** **‐Me_2_‐2.2.2]** was detected, while in the negative mode only **[Cl** ^−^ ⊂ ***o*** **‐Me_2_‐ur‐C2]** was observed. No larger complexes or mixed cryptates containing both kinds of diols were found (Figure 5d).

Next, we attempted to remove the CsCl from this unusual dual‐host ion pair receptor.^[32]^ Having failed with other methods (e.g. washing with MeOH or a solution of NaOH in D_2_O), we found that repeated washing of the precipitate with D_2_O decreased the amount of salt, such that only traces of Cs^+^ were detectable by ^133^Cs‐NMR (Figure S33). To gain further insights into the process of cryptate formation, we decided to deliberately inhibit the template effect of CsCl by using only dimethyl sulfoxide as a highly competitive solvent (Figures S35 and S36). While ^1^H‐NMR spectroscopy indicated that orthoester exchange proceeded successfully, no signals of the two expected cryptates could be detected by ESI‐MS. We deduce from this finding that the Cs^+^ and Cl^−^ ions indeed act in concert to drive the self‐assembly reaction forward.

To gain deeper insights into the driving force behind the formation of each cryptate, we performed the self‐assembly reaction with under‐stoichiometric amounts of trimethyl orthoacetate (2.1 equiv) such that diols **TEG** and **1** have to compete for the orthoester. In this experiment, we still observed the formation of both cryptates, but no longer in a 1 : 1 ratio. In the non‐competitive experiment, cryptates **[Cl** ^−^ ⊂ ***o*** **‐Me_2_‐ur‐C2]** and **[Cs** ^+^ ⊂ ***o*** **‐Me_2_‐2.2.2]** are formed in ca. 1 : 1 ratio (as determined by quant. ^13^C‐NMR spectroscopy). However, when using only half of the orthoester, the cryptates form in ca. 2 : 1 ratio, thus suggesting that the formation of the chloride cryptate is somewhat preferred. Additionally, we carried out the self‐assemblies of each cryptate separately using CsCl as the source for the Cs^+^ or Cl^−^ ion (previously these two ions had been complemented with weakly coordinating counterions BArF^−^ and PPh_4_^+^, respectively). Interestingly, compound **[Cl** ^−^ ⊂ ***o*** **‐Me_2_‐ur‐C2]Cs** ^+^ was obtained in 42% yield (Chapter 5.6 in the Supporting Information) in this way, whereas only traces of **[Cs** ^+^ ⊂ ***o*** **‐Me_2_‐2.2.2]Cl** ^−^ could be detected in this attempt to prepare this compound from CsCl (Chapter 5.7 in the Supporting Information). We conclude from these three experiments that the formation of the chloride cryptate is thermodynamically highly preferred in this solvent mixture (CDCl_3_/DMSO‐d_6_ 5 : 1) and provides most of the driving force in mixed experiments. In contrast, the cesium cryptate **[Cs** ^+^ ⊂ ***o*** **‐Me_2_‐2.2.2]** is only formed in the dual assembly experiment as a consequence of the formation of **[Cl** ^−^ ⊂ ***o*** **‐Me_2_‐ur‐C2]**, and the chloride cryptate can be seen as a large, weakly coordinating anion that enhances the Cs^+^ template effect. While the supramolecular binding of anions is generally seen as more difficult than the binding of cations, our CsCl‐driven dual host self‐assembly clearly represents a case where the opposite is true (at least in CDCl_3_/DMSO‐d_6_ 5 : 1).

## Conclusion

We report the self‐assembly of macrobicyclic hosts for simple anions based on the urea binding motif and orthoester bridgeheads. The cryptands and cryptates exhibit complex intramolecular hydrogen bonding equilibria that may be relevant in most multivalent anion receptors and transporters possessing a certain degree of flexibility. Our self‐assembly approach allowed facile access to (mixtures of) heteroleptic host–guest complexes, which, thanks to tandem mass spectrometry, could be studied in the gas‐phase without need of isolation in bulk. Having established both anion‐ and cation‐templated orthoester hosts, we attempted to combine the two chemistries in one pot and were indeed able to realize such a dual self‐assembly with CsCl playing the role of an “ion pair template”. Future work will focus on more water‐soluble building blocks for membrane transport and release studies.

## Conflict of interest

The authors declare no conflict of interest.

1

## Supporting information

As a service to our authors and readers, this journal provides supporting information supplied by the authors. Such materials are peer reviewed and may be re‐organized for online delivery, but are not copy‐edited or typeset. Technical support issues arising from supporting information (other than missing files) should be addressed to the authors.

Supporting Information

## Data Availability

The data that support the findings of this study are openly available in supramolecular.org at http://app.supramolecular.org/bindfit/view/442e7e14‐12ab‐4beb‐81fb‐0c5b3837e0ef and Figshare https://doi.org/10.6084/m9.figshare.19086995.

## References

[1a] H. E. Simmons , C. H. Park , J. Am. Chem. Soc. 1968, 90, 2428–2429;

[1b] C. H. Park , H. E. Simmons , J. Am. Chem. Soc. 1968, 90, 2429–2431;

[1c] C. H. Park , H. E. Simmons , J. Am. Chem. Soc. 1968, 90, 2431–2432;

[1d] J. Lehn , E. Sonveaux , A. K. Willard , J. Am. Chem. Soc. 1978, 100, 4914–4916;

[1e] F. P. Schmidtchen , M. Berger , Chem. Rev. 1997, 97, 1609–1646. 11851460 10.1021/cr9603845

[2a] S. O. Kang , J. M. Llinares , V. W. Day , K. Bowman-James , Chem. Soc. Rev. 2010, 39, 3980–4003; 20820597 10.1039/c0cs00083c

[2b] P. Ballester , Chem. Soc. Rev. 2010, 39, 3810–3830; 20820466 10.1039/b926229f

[2c] U. Manna , G. Das , Coord. Chem. Rev. 2021, 427, 213547;

[2d] J. Zhao , D. Yang , X. J. Yang , B. Wu , Coord. Chem. Rev. 2019, 378, 415–444;

[2e] M. Delecluse , C. Colomban , B. Chatelet , S. Chevallier-Michaud , D. Moraleda , J. P. Dutasta , A. Martinez , J. Org. Chem. 2020, 85, 4706–4711; 32153196 10.1021/acs.joc.9b03429

[2f] H. A. Fargher , N. Lau , H. C. Richardson , P. H. Y. Cheong , M. M. Haley , M. D. Pluth , D. W. Johnson , J. Am. Chem. Soc. 2020, 142, 8243–8251; 32283020 10.1021/jacs.0c00441 PMC7392143

[2g] M. J. Langton , S. W. Robinson , I. Marques , V. Félix , P. D. Beer , Nat. Chem. 2014, 6, 1039–1043; 25411880 10.1038/nchem.2111

[2h] C. J. Serpell , A. Y. Park , C. V. Robinson , P. D. Beer , Chem. Commun. 2021, 57, 101–104; 10.1039/d0cc06299e 33337451

[2i] C. L. Deng , J. P. Bard , J. A. Lohrman , J. E. Barker , L. N. Zakharov , D. W. Johnson , M. M. Haley , Angew. Chem. Int. Ed. 2019, 58, 3934–3938; 10.1002/anie.201814431 30702802

[3a] L. K. Macreadie , A. M. Gilchrist , D. A. McNaughton , W. G. Ryder , M. Fares , P. A. Gale , Chem 2022, 8, 46–118;

[3b] L. Chen , S. N. Berry , X. Wu , E. N. W. Howe , P. A. Gale , Chem 2020, 6, 61–141;

[3c] P. A. Gale , E. N. W. Howe , X. Wu , Chem 2016, 1, 351–422;

[3d] N. Busschaert , C. Caltagirone , W. Van Rossom , P. A. Gale , Chem. Rev. 2015, 115, 8038–8155; 25996028 10.1021/acs.chemrev.5b00099

[3e] N. H. Evans , P. D. Beer , Angew. Chem. Int. Ed. 2014, 53, 11716–11754; 10.1002/anie.201309937 25204549

[4a] R. Hein , P. D. Beer , J. J. Davis , Chem. Rev. 2020, 120, 1888–1935; 31916758 10.1021/acs.chemrev.9b00624

[4b] P. A. Gale , C. Caltagirone , Chem. Soc. Rev. 2015, 44, 4212–4227; 24975326 10.1039/c4cs00179f

[4c] W. Chen , C. Guo , Q. He , X. Chi , V. M. Lynch , Z. Zhang , J. Su , H. Tian , J. L. Sessler , J. Am. Chem. Soc. 2019, 141, 14798–14806; 31437397 10.1021/jacs.9b07170

[4d] A.-F. Li , J.-H. Wang , F. Wang , Y.-B. Jiang , Chem. Soc. Rev. 2010, 39, 3595–3596;

[4e] P. D. Beer , P. A. Gale , Angew. Chem. Int. Ed. 2001, 40, 486–516; 11180358

[4f] J. Bartl , L. Reinke , M. Koch , S. Kubik , Chem. Commun. 2020, 56, 10457–10460; 10.1039/d0cc04796a 32856639

[4g] D. P. Farrell , A. L. Sargent , W. E. Allen , Supramol. Chem. 2016, 28, 45–52;

[4h] D. A. McNaughton , M. Fares , G. Picci , P. A. Gale , C. Caltagirone , Coord. Chem. Rev. 2021, 427, 213573;

[4i] C. L. Vonnegut , A. M. Shonkwiler , L. N. Zakharov , M. M. Haley , D. W. Johnson , Chem. Commun. 2016, 52, 9506–9509; 10.1039/c6cc03795j PMC4973467 27375117

[4j] C. G. Collins , E. M. Peck , P. J. Kramer , B. D. Smith , Chem. Sci. 2013, 4, 2557–2563;

[4k] C. F. Chow , P. Y. Ho , C.-B. Gong , Analyst 2014, 139, 4256–4263. 24989109 10.1039/c4an00350k

[5a] X. Wu , E. N. W. Howe , P. A. Gale , Acc. Chem. Res. 2018, 51, 1870–1879; 30063324 10.1021/acs.accounts.8b00264

[5b] X. Wu , A. M. Gilchrist , P. A. Gale , Chem 2020, 6, 1296–1309;

[5c] A. Vargas Jentzsch , A. Hennig , J. Mareda , S. Matile , Acc. Chem. Res. 2013, 46, 2791–2800; 23547885 10.1021/ar400014r

[5d] M. J. Spooner , H. Li , I. Marques , P. M. R. Costa , X. Wu , E. N. W. Howe , N. Busschaert , S. J. Moore , M. E. Light , D. N. Sheppard , V. Félix , P. A. Gale , Chem. Sci. 2019, 10, 1976–1985; 30881627 10.1039/c8sc05155k PMC6381411

[5e] I. Carreira-Barral , M. Mielczarek , D. Alonso-Carrillo , V. Capurro , V. Soto-Cerrato , R. Pérez Tomás , E. Caci , M. García-Valverde , R. Quesada , Chem. Commun. 2020, 56, 3218–3221; 10.1039/d0cc00643b 32073062

[5f] G. Picci , I. Carreira-Barral , D. Alonso-Carrillo , D. Sanz-González , P. Fernández-López , M. García-Valverde , C. Caltagirone , R. Quesada , Supramol. Chem. 2020, 32, 112–118;

[5g] G. Grauwels , H. Valkenier , A. P. Davis , I. Jabin , K. Bartik , Angew. Chem. Int. Ed. 2019, 58, 6921–6925; 10.1002/anie.201900818 30925004

[5h] C. M. Dias , H. Li , H. Valkenier , L. E. Karagiannidis , P. A. Gale , D. N. Sheppard , A. P. Davis , Org. Biomol. Chem. 2018, 16, 1083–1087; 29376532 10.1039/c7ob02787g

[5i] X. Wu , L. W. Judd , E. N. W. Howe , A. M. Withecombe , V. Soto-Cerrato , H. Li , N. Busschaert , H. Valkenier , R. Pérez-Tomás , D. N. Sheppard , Y. B. Jiang , A. P. Davis , P. A. Gale , Chem 2016, 1, 127–146;

[5j] H. Valkenier , O. Akrawi , P. Jurček , K. Sleziaková , T. Lízal , K. Bartik , V. Šindelář , Chem 2019, 5, 429–444;

[5k] L. Martínez-Crespo , L. Halgreen , M. Soares , I. Marques , V. Félix , H. Valkenier , Org. Biomol. Chem. 2021, 19, 8324–8337. 34523662 10.1039/d1ob01279g

[6a] X. Ji , R. T. Wu , L. Long , C. Guo , N. M. Khashab , F. Huang , J. L. Sessler , J. Am. Chem. Soc. 2018, 140, 2777–2780; 29437394 10.1021/jacs.7b13656

[6b] M. T. Albelda , J. C. Frías , E. García-España , H. J. Schneider , Chem. Soc. Rev. 2012, 41, 3859–3877; 22441360 10.1039/c2cs35008d

[6c] D. Zhang , T. K. Ronson , J. Mosquera , A. Martinez , J. R. Nitschke , Angew. Chem. Int. Ed. 2018, 57, 3717–3721; 10.1002/anie.201800459 PMC6001518 29393989

[6d] W. Liu , A. G. Oliver , B. D. Smith , J. Org. Chem. 2019, 84, 4050–4057; 30827107 10.1021/acs.joc.9b00042

[6e] R. B. P. Elmes , K. K. Y. Yuen , K. A. Jolliffe , Chem. Eur. J. 2014, 20, 7373–7380; 24828677 10.1002/chem.201400292

[6f] A. Thevenet , A. Miljkovic , S. La Cognata , C. Marie , C. Tamain , N. Boubals , C. Mangano , V. Amendola , P. Guilbaud , Dalton Trans. 2021, 50, 1620–1630; 33470269 10.1039/d0dt04210b

[6g] C. Jia , B. Wu , S. Li , X. Huang , Q. Zhao , Q. S. Li , X. J. Yang , Angew. Chem. Int. Ed. 2011, 50, 486–490; 10.1002/anie.201004461 21132821

[7a] P. A. Gale , J. T. Davis , R. Quesada , Chem. Soc. Rev. 2017, 46, 2497–2519; 28379234 10.1039/c7cs00159b

[7b] J. T. Davis , P. A. Gale , R. Quesada , Chem. Soc. Rev. 2020, 49, 6056–6086; 10.1039/c9cs00662a 32692794

[7c] S. H. Park , S. H. Park , E. N. W. Howe , J. Y. Hyun , L. J. Chen , I. Hwang , G. Vargas-Zuñiga , N. Busschaert , P. A. Gale , J. L. Sessler , I. Shin , Chem 2019, 5, 2079–2098; 33791443 10.1016/j.chempr.2019.05.001 PMC8009298

[7d] H. Li , H. Valkenier , A. G. Thorne , C. M. Dias , J. A. Cooper , M. Kieffer , N. Busschaert , P. A. Gale , D. N. Sheppard , A. P. Davis , Chem. Sci. 2019, 10, 9663–9672; 32055336 10.1039/c9sc04242c PMC6984391

[7e] N. Busschaert , S. H. Park , K. H. Baek , Y. P. Choi , J. Park , E. N. W. Howe , J. R. Hiscock , L. E. Karagiannidis , I. Marques , V. Félix , W. Namkung , J. L. Sessler , P. A. Gale , I. Shin , Nat. Chem. 2017, 9, 667–675; 28644464 10.1038/nchem.2706 PMC5648535

[7f] S. K. Ko , S. K. Kim , A. Share , V. M. Lynch , J. Park , W. Namkung , W. Van Rossom , N. Busschaert , P. A. Gale , J. L. Sessler , I. Shin , Nat. Chem. 2014, 6, 885–892; 25242483 10.1038/nchem.2021

[7g] N. Akhtar , O. Biswas , D. Manna , Chem. Commun. 2020, 56, 14137–14153; 10.1039/d0cc05489e 33057487

[7h] L. A. Marchetti , L. K. Kumawat , N. Mao , J. C. Stephens , R. B. P. Elmes , Chem 2019, 5, 1398–1485;

[7i] L. Tapia , I. Alfonso , J. Solà , Org. Biomol. Chem. 2021, 19, 9527–9540; 34668919 10.1039/d1ob01737c

[7j] A. Roy , P. Talukdar , ChemBioChem 2021, 22, 2925–2940; 34043277 10.1002/cbic.202100112 PMC8596773

[7k] J. Yang , G. Yu , J. L. Sessler , I. Shin , P. A. Gale , F. Huang , Chem 2021, 7, 3256–3291.

[8a] Y. Liu , W. Zhao , C.-H. Chen , A. H. Flood , Science 2019, 365, 159–161; 31123106 10.1126/science.aaw5145

[8b] F. C. Parks , E. G. Sheetz , S. R. Stutsman , A. Lutolli , S. Debnath , K. Raghavachari , A. H. Flood , J. Am. Chem. Soc. 2022, 144, 1274–1287. 35015538 10.1021/jacs.1c10758

[9a] C. T. McTernan , T. K. Ronson , J. R. Nitschke , J. Am. Chem. Soc. 2021, 143, 664–670; 33382246 10.1021/jacs.0c11905 PMC7879535

[9b] M. Kieffer , R. A. Bilbeisi , J. D. Thoburn , J. K. Clegg , J. R. Nitschke , Angew. Chem. Int. Ed. 2020, 59, 11369–11373; 10.1002/anie.202004627 PMC7383889 32243707

[9c] Z. Lu , T. K. Ronson , J. R. Nitschke , Chem. Sci. 2020, 11, 1097–1101; 10.1039/c9sc05728e PMC8146419 34084365

[9d] S. Wang , Z. Huang , A. Li , Y. Zhao , W. Zuo , Y. Li , H. Miao , J. Ma , W. Sun , X. Wang , L. Cao , B. Wu , C. Jia , Angew. Chem. Int. Ed. 2021, 60, 9573–9579; 10.1002/anie.202100441 33586834

[9e] S. J. Allison , J. Bryk , C. J. Clemett , R. A. Faulkner , M. Ginger , H. B. S. Griffiths , J. Harmer , P. J. Owen-Lynch , E. Pinder , H. Wurdak , R. M. Phillips , C. R. Rice , Nat. Commun. 2021, 12, 3898; 34162854 10.1038/s41467-021-23983-3 PMC8222254

[9f] R. Andrews , S. Begum , C. J. Clemett , R. A. Faulkner , M. L. Ginger , J. Harmer , M. Molinari , G. M. B. Parkes , Z. M. H. Qureshi , C. R. Rice , M. D. Ward , H. M. Williams , P. B. Wilson , Angew. Chem. Int. Ed. 2020, 59, 20480–20484; 10.1002/anie.202009960 PMC7693201 32743891

[10a] W. Zhang , Y. Feng , B. Li , D. Yang , L. Hou , W. Zhao , X. J. Yang , B. Wu , Chem. Eur. J. 2022, 28, e20210367; 10.1002/chem.202103671 34687106

[10b] J. Fu , B. Zheng , H. Zhang , Y. Zhao , D. Zhang , W. Zhang , X. J. Yang , B. Wu , Chem. Commun. 2020, 56, 2475–2478; 10.1039/c9cc09752j 31998905

[10c] W. Zhang , D. Yang , J. Zhao , L. Hou , J. L. Sessler , X. J. Yang , B. Wu , J. Am. Chem. Soc. 2018, 140, 5248–5256; 29584424 10.1021/jacs.8b01488

[10d] X. Bai , C. Jia , Y. Zhao , D. Yang , S. C. Wang , A. Li , Y. T. Chan , Y. Y. Wang , X. J. Yang , B. Wu , Angew. Chem. Int. Ed. 2018, 57, 1851–1855; 10.1002/anie.201712080 29251815

[10e] D. Yang , J. Zhao , L. Yu , X. Lin , W. Zhang , H. Ma , A. Gogoll , Z. Zhang , Y. Wang , X. J. Yang , B. Wu , J. Am. Chem. Soc. 2017, 139, 5946–5951; 28335592 10.1021/jacs.7b01890

[10f] D. Yang , J. Zhao , Y. Zhao , Y. Lei , L. Cao , X. J. Yang , M. Davi , N. De Sousa Amadeu , C. Janiak , Z. Zhang , Y. Y. Wang , B. Wu , Angew. Chem. Int. Ed. 2015, 54, 8658–8661; 10.1002/anie.201502399 26053734

[10g] B. Wu , F. Cui , Y. Lei , S. Li , N. De Sousa Amadeu , C. Janiak , Y. J. Lin , L. H. Weng , Y. Y. Wang , X. J. Yang , Angew. Chem. Int. Ed. 2013, 52, 5096–5100; 10.1002/anie.201209930 23564728

[11a] Z. Rodriguez-Docampo , E. Eugenieva-Ilieva , C. Reyheller , A. M. Belenguer , S. Kubik , S. Otto , Chem. Commun. 2011, 47, 9798–9800; 10.1039/c1cc13451e 21808782

[11b] S. Otto , S. Kubik , J. Am. Chem. Soc. 2003, 125, 7804–7805. 12822990 10.1021/ja0351589

[12a] H. Xie , T. J. Finnegan , V. W. Liyana Gunawardana , R. Z. Pavlović , C. E. Moore , J. D. Badjić , J. Am. Chem. Soc. 2021, 143, 3874–3880; 33656878 10.1021/jacs.0c12329

[12b] E. A. Katayev , G. D. Pantos , M. D. Reshetova , V. N. Khrustalev , V. M. Lynch , Y. A. Ustynyuk , J. L. Sessler , Angew. Chem. Int. Ed. 2005, 44, 7386–7390; 10.1002/anie.200502393 16245380

[12c] S. R. Beeren , J. K. M. Sanders , J. Am. Chem. Soc. 2011, 133, 3804–3807. 21361379 10.1021/ja200130h

[13a] L. Tapia , Y. Pérez , M. Bolte , J. Casas , J. Solà , R. Quesada , I. Alfonso , Angew. Chem. Int. Ed. 2019, 58, 12465–12468; 10.1002/anie.201905965 31298461

[13b] H. Fernández-Caro , I. Lostalé-Seijo , M. Martínez-Calvo , J. Mosquera , J. L. Mascareñas , J. Montenegro , Chem. Sci. 2019, 10, 8930–8938; 32110291 10.1039/c9sc02906k PMC7017865

[13c] X. Chi , W. Cen , J. A. Queenan , L. Long , V. M. Lynch , N. M. Khashab , J. L. Sessler , J. Am. Chem. Soc. 2019, 141, 6468–6472; 30957995 10.1021/jacs.9b01241

[13d] M. Fares , X. Wu , D. Ramesh , W. Lewis , P. A. Keller , E. N. W. Howe , R. Pérez-Tomás , P. A. Gale , Angew. Chem. Int. Ed. 2020, 59, 17614–17621; 10.1002/anie.202006392 32583552

[13e] A. Kerckhoffs , Z. Bo , S. E. Penty , F. Duarte , M. J. Langton , Org. Biomol. Chem. 2021, 19, 9058–9067; 34617944 10.1039/d1ob01457a

[13f] S. J. Wezenberg , L.-J. Chen , J. E. Bos , B. L. Feringa , E. N. W. Howe , X. Wu , M. A. Siegler , P. A. Gale , J. Am. Chem. Soc. 2022, 144, 331–338; 34932344 10.1021/jacs.1c10034 PMC8759083

[13g] Y. Eygeris , M. Gupta , J. Kim , G. Sahay , Acc. Chem. Res. 2022, 55, 2–12. 34850635 10.1021/acs.accounts.1c00544

[14a] C. Jia , W. Zuo , D. Zhang , X. J. Yang , B. Wu , Chem. Commun. 2016, 52, 9614–9627; 10.1039/c6cc03761e 27352298

[14b] V. Blažek Bregović , N. Basarić , K. Mlinarić-Majerski , Coord. Chem. Rev. 2015, 295, 80–124;

[14c] J. Zhao , D. Yang , Y. Zhao , L. Cao , Z. Zhang , X. J. Yang , B. Wu , Dalton Trans. 2016, 45, 7360–7365; 27028977 10.1039/c6dt00672h

[14d] N. Busschaert , P. A. Gale , C. J. E. Haynes , M. E. Light , S. J. Moore , C. C. Tong , T. Davis , W. A. Harrell , Chem. Commun. 2010, 46, 6252–6254; 10.1039/c0cc01684e 20694202

[14e] A. Basu , G. Das , J. Org. Chem. 2014, 79, 2647–2656. 24558963 10.1021/jo500102e

[15a] A. Herrmann , Chem. Soc. Rev. 2014, 43, 1899–1933; 24296754 10.1039/c3cs60336a

[15b] Y. Jin , C. Yu , R. J. Denman , W. Zhang , Chem. Soc. Rev. 2013, 42, 6634–6654; 23749182 10.1039/c3cs60044k

[15c] Y. Jin , Q. Wang , P. Taynton , W. Zhang , Acc. Chem. Res. 2014, 47, 1575–1586; 24739018 10.1021/ar500037v

[15d] J. Li , P. Nowak , S. Otto , J. Am. Chem. Soc. 2013, 135, 9222–9239; 23731408 10.1021/ja402586c

[15e] P. T. Corbett , J. Leclaire , L. Vial , K. R. West , J. Wietor , J. K. M. Sanders , S. Otto , Chem. Rev. 2006, 106, 3652–3711; 16967917 10.1021/cr020452p

[15f] S. J. Rowan , S. J. Cantrill , G. R. L. Cousins , J. K. M. Sanders , J. F. Stoddart , Angew. Chem. Int. Ed. 2002, 41, 898–952; 10.1002/1521-3773(20020315)41:6<898::aid-anie898>3.0.co;2-e 12491278

[15g] J. F. Reuther , S. D. Dahlhauser , E. V. Anslyn , Angew. Chem. Int. Ed. 2019, 58, 74–85; 10.1002/anie.201808371 PMC10851707 30098086

[15h] E. Moulin , G. Cormos , N. Giuseppone , Chem. Soc. Rev. 2012, 41, 1031–1049; 21909573 10.1039/c1cs15185a

[15i] Dynamic Covalent Chemistry: Principles, Reactions, and Applications (Eds.: W. Zhang , Y. Jin ), Wiley-VCH, Weinheim, 2018.

[16a] R. C. Brachvogel , M. von Delius , Chem. Sci. 2015, 6, 1399–1403; 29560228 10.1039/c4sc03528c PMC5811105

[16b] R. C. Brachvogel , M. von Delius , Eur. J. Org. Chem. 2016, 3662–3670.

[17a] R. C. Brachvogel , F. Hampel , M. von Delius , Nat. Commun. 2015, 6, 7129; 25997913 10.1038/ncomms8129 PMC4455094

[17b] O. Shyshov , R. C. Brachvogel , T. Bachmann , R. Srikantharajah , D. Segets , F. Hampel , R. Puchta , M. von Delius , Angew. Chem. Int. Ed. 2017, 56, 776–781; 10.1002/anie.201609855 27958672

[18] H. Löw , E. Mena-Osteritz , M. von Delius , Chem. Sci. 2018, 9, 4785–4793. 29910929 10.1039/c8sc01750f PMC5982201

[19a] J. Zhou , L. Rao , G. Yu , T. R. Cook , X. Chen , F. Huang , Chem. Soc. Rev. 2021, 50, 2839–2891; 33524093 10.1039/d0cs00011f

[19b] J. Zhou , G. Yu , F. Huang , Chem. Soc. Rev. 2017, 46, 7021–7053; 28980674 10.1039/c6cs00898d

[19c] D. A. Uhlenheuer , K. Petkau , L. Brunsveld , Chem. Soc. Rev. 2010, 39, 2817–2826; 20461247 10.1039/b820283b

[19d] G. Gasparini , E. K. Bang , J. Montenegro , S. Matile , Chem. Commun. 2015, 51, 10389–10402. 10.1039/c5cc03472h 26030211

[20] L. H. Perruchoud , A. Hadzovic , X. Zhang , Chem. Eur. J. 2015, 21, 8711–8715. 25931372 10.1002/chem.201500989

[21a] X. Wang , O. Shyshov , M. Hanževački , C. M. Jäger , M. von Delius , J. Am. Chem. Soc. 2019, 141, 8868–8876; 31117548 10.1021/jacs.9b01350

[21b] H. Löw , E. Mena-Osteritz , K. M. Mullen , C. M. Jäger , M. von Delius , ChemPlusChem 2020, 85, 1008–1012. 32347636 10.1002/cplu.202000254

[22] A. M. Rijs , E. R. Kay , D. A. Leigh , W. J. Buma , J. Phys. Chem. A 2011, 115, 9669–9675. 21524109 10.1021/jp200909v

[23] G. Jakab , C. Tancon , Z. Zhang , K. M. Lippert , P. R. Schreiner , Org. Lett. 2012, 14, 1724–1727. 22435999 10.1021/ol300307c

[24a] P. Thordarson , Chem. Soc. Rev. 2011, 40, 1305–1323; 21125111 10.1039/c0cs00062k

[24b] D. Brynn Hibbert , P. Thordarson , Chem. Commun. 2016, 52, 12792–12805. 10.1039/c6cc03888c 27779264

[25] R. A. Hovermale , P. G. Sears , J. Phys. Chem. 1956, 60, 1579–1580.

[26] H. Löw , E. Mena-Osteritz , M. von Delius , Chem. Commun. 2019, 55, 11434–11437. 10.1039/c9cc05968g 31486819

[27] C. J. E. Haynes , N. Busschaert , I. L. Kirby , J. Herniman , M. E. Light , N. J. Wells , I. Marques , V. Félix , P. A. Gale , Org. Biomol. Chem. 2014, 12, 62–72. 24056984 10.1039/c3ob41522h

[28a] S. J. Moore , C. J. E. Haynes , J. González , J. L. Sutton , S. J. Brooks , M. E. Light , J. Herniman , G. J. Langley , V. Soto-Cerrato , R. Pérez-Tomás , I. Marques , P. J. Costa , V. Félix , P. A. Gale , Chem. Sci. 2013, 4, 103–117;

[28b] S. J. Edwards , H. Valkenier , N. Busschaert , P. A. Gale , A. P. Davis , Angew. Chem. Int. Ed. 2015, 54, 4592–4596; 10.1002/anie.201411805 PMC4405043 25690527

[29a] M. B. Minameyer , Y. Xu , S. Frühwald , A. Görling , M. von Delius , T. Drewello , Chem. Eur. J. 2020, 26, 8729–8741; 32476186 10.1002/chem.202001503 PMC7497255

[29b] Z. Qi , T. Heinrich , S. Moorthy , C. A. Schalley , Chem. Soc. Rev. 2015, 44, 515–531. 24956973 10.1039/c4cs00167b

[30] R.-C. Brachvogel , H. Maid , M. von Delius , Int. J. Mol. Sci. 2015, 16, 20641–20656. 26334274 10.3390/ijms160920641 PMC4613223

[31] D. Zhang , T. K. Ronson , J. L. Greenfield , T. Brotin , P. Berthault , E. Leónce , J. L. Zhu , L. Xu , J. R. Nitschke , J. Am. Chem. Soc. 2019, 141, 8339–8345. 31034215 10.1021/jacs.9b02866

[32a] M. Cametti , M. Nissinen , A. Dalla Cort , L. Mandolini , K. Rissanen , J. Am. Chem. Soc. 2005, 127, 3831–3837; 15771518 10.1021/ja042807n

[32b] Q. He , G. I. Vargas-Zúñiga , S. H. Kim , S. K. Kim , J. L. Sessler , Chem. Rev. 2019, 119, 9753–9835; 31081334 10.1021/acs.chemrev.8b00734

[32c] S. K. Kim , J. L. Sessler , Chem. Soc. Rev. 2010, 39, 3784–3809; 20737073 10.1039/c002694h PMC3016456

[32d] A. J. McConnell , P. D. Beer , Angew. Chem. Int. Ed. 2012, 51, 5052–5061; 10.1002/anie.201107244 22419667

[32e] A. J. McConnell , A. Docker , P. D. Beer , ChemPlusChem 2020, 85, 1824–1841; 32833334 10.1002/cplu.202000484

[32f] J. H. Yang , V. M. Lynch , J. L. Sessler , S. K. Kim , Supramol. Chem. 2019, 31, 203–210; 32523324 10.1080/10610278.2018.1535711 PMC7286541

[32g] R. C. Knighton , P. D. Beer , Org. Chem. Front. 2021, 8, 2468–2472;

[32h] Y. C. Tse , A. Docker , Z. Zhang , P. D. Beer , Chem. Commun. 2021, 57, 4950–4953; 10.1039/d1cc01287h 33876159

[32i] Z. Kokan , M. J. Chmielewski , J. Am. Chem. Soc. 2018, 140, 16010–16014. 30415543 10.1021/jacs.8b08689

