# Supplementary material for: Dynamic Covalent Self‐Assembly of Chloride‐ and Ion‐Pair‐Templated Cryptates
Source: Angew Chem Int Ed Engl. 2022 May 12;61(28):e202201831. doi: 10.1002/anie.202201831 (PMC9400851; doi:10.1002/anie.202201831)
Supplement: Supplementary file 1 — Supporting Information [file ANIE-61-0-s001.pdf]

Angewandte Chemie
Eine Zeitschrift der Gesellschaft Deutscher Chemiker

Supporting Information

Dynamic Covalent Self-Assembly of Chloride- and Ion-Pair-Templated Cryptates

*S. Hollstein, O. Shyshov, M. Hanževački, J. Zhao, T. Rudolf, C. M. Jäger, M. von Delius**

Table of Contents

1. General Experimental Section ... 3
2. Synthesis and Characterization of Diol Building Blocks ... 5
Synthesis of 1,3-bis(3-(2-hydroxyethyl)phenyl)urea (**1**) ... 5
Synthesis of 1,3-bis(3-(hydroxymethyl)phenyl)urea (**S9**) ... 7
Synthesis of 1,3-bis(3-(3-hydroxypropyl)phenyl)urea (**2**) ... 8
Synthesis of 1,3-bis(3-(4-hydroxybutyl)phenyl)urea (**3**) ... 10
3. Synthesis and Characterization of Chloride-Templated Cryptates ... 11
3.1 General Procedures ... 11
3.2 Characterization of Cryptates ... 13
Characterization of **[Cl$^-$⊂*o*-Me$_2$-ur-C2]PPh$_4$$^+$** and ***o*-Me$_2$-ur-C2** ... 13
Characterization of **[Cl$^-$⊂*o*-Me$_2$-ur-C3]PPh$_4$$^+$** and ***o*-Me$_2$-ur-C3** ... 16
Characterization of **[Cl$^-$⊂*o*-Me$_2$-ur-C4]PPh$_4$$^+$** and ***o*-Me$_2$-ur-C4** ... 20
Characterization of **[Cl$^-$⊂*o*-H$_2$-ur-C4]PPh$_4$$^+$** and ***o*-H$_2$-ur-C4** ... 24
4. Synthesis and Characterization of Ion-Pair-Templated Cryptates ... 28
5. Miscellaneous Experiments ... 34
5.1 Evidence for template effect I: Attempting synthesis of ***o*-Me$_2$-ur-C3** without PPh$_4$Cl ... 34
5.2 Evidence for template effect II: Unsuccessful synthesis of **[Cl$^-$⊂*o*-Me$_2$-ur-C2][Cs$^+$⊂*o*-Me$_2$-2.2.2**] in pure DMSO ... 35
5.3 Comparison of ATR-IR spectra of **[Cl$^-$⊂*o*-Me$_2$-ur-C2][Cs$^+$⊂*o*-Me$_2$-2.2.2]**, **[Cl$^-$⊂*o*-Me$_2$-ur-C2]PPh$_3$$^+$** and **[Cs$^+$⊂*o*-Me$_2$-2.2.2]BArF$^-$** ... 37
5.4 ^{1}H NMR and ^{133}Cs NMR studies of binding of CsCl to **[Cl$^-$⊂*o*-Me$_2$-ur-C2][Cs$^+$⊂*o*-Me$_2$-2.2.2]** 38
5.5 Competition Experiment: Synthesis of **[Cl$^-$⊂*o*-Me$_2$-ur-C2][Cs$^+$⊂*o*-Me$_2$-2.2.2]** with under-stoichiometric amounts of trimethyl orthoacetate ... 39
5.6 Using CsCl as a template: Synthesis of **[Cl$^-$⊂*o*-Me$_2$-ur-C2]Cs$^+$** ... 42
5.7 Using CsCl as a template: Unsuccessful synthesis of **[Cs$^+$⊂*o*-Me$_2$-2.2.2]Cl$^-$** ... 43
6. Computational Chemistry ... 44
7. Host-Guest Titrations ... 46
7.1 General Procedure ... 46
7.2 NMR Titration Data, Isotherms and Residuals ... 48
***o*-Me$_2$-ur-C2** and TPPCl in DMSO-d$_6$ ... 48
***o*-Me$_2$-ur-C2** and TPPBr in DMSO-d$_6$ ... 50
***o*-Me$_2$-ur-C2** and TPPI in DMSO-d$_6$ ... 51
***o*-Me$_2$-ur-C2** and TMANO$_3$ in DMSO-d$_6$ ... 52
***o*-Me$_2$-ur-C3** and TPPCl in DMSO-d$_6$ ... 53
***o*-Me$_2$-ur-C4** and TPPCl in DMSO-d$_6$ ... 54
***o*-H$_2$-ur-C4** and TPPCl in DMSO-d$_6$ ... 55
***o*-Me$_2$-ur-C2** and TPPCl in CDCl$_3$/DMSO-d$_6$ (5:1) ... 56
8. High resolution ESI-MS/MS measurements ... 57
9. NMR Spectra ... 59
References ... 71
Author Contributions ... 71

1. General Experimental Section

All commercially available chemicals were purchased from Sigma Aldrich, ABCR GmbH, TCI Deutschland GmbH, Acros Organics and Alfa Aesar and used without further purification. $CDCl_3$ and DMSO-d_6 were stored over molecular sieves (3 Å). Molecular sieves and aluminum oxide were dried at 150 °C under reduced pressure (10^{-2} mbar). Anhydrous solvents were dried prior to use in a MBraun SPS-800 instrument. All solvents used in exchange reactions were stored over molecular sieves (3 Å) for at least 24 hours prior to usage. All orthoester exchange reactions were carried out under nitrogen atmosphere.

NMR spectra were recorded on Bruker Avance 400, Bruker Avance Neo 400, Bruker Avance 500 or Bruker Avance Neo 600 spectrometers (^{1}H: 400 MHz, 500 MHz or 600 MHz, ^{13}C: 101 MHz or 151 MHz, ^{133}Cs: 52 MHz) at 293 K. The spectra were calibrated to the residual solvent peaks (^{1}H NMR: 7.26 ppm ($CDCl_3$), 2.50 ppm (DMSO-d_6), ^{13}C NMR: 77.16 ppm ($CDCl_3$), 39.52 ppm (DMSO-d_6)). Chemical shifts (δ) are denoted in ppm and coupling constants (*J*) in Hz.

High resolution mass spectra were recorded on Bruker solariX (Hybrid 7T FT-ICR) or Agilent QTOF 6546 using electrospray ionization (ESI). Acetonitrile was used as solvent.

ATR-IR spectra were recorded on a Bruker Alpha II with an ATR platinum Diamond using 60 scans in a range of 400 to 4000 cm^{-1}.

The normal-phase flash column chromatography was performed using silica 60 with a particle size of 0.04 – 0.063 mm from Macherey-Nagel.

Molecular dynamics (MD) simulations were carried out with Amber 20 software package using the GPU accelerated *pmemd.cuda* module.[1] The General Amber Force Field (GAFF) parameters were used to define bonded and non-bonded parameters of the cryptand.[2] The partial charges have been derived applying the restrained electrostatic potential (RESP)[3] point charge fitting procedure based on the gas phase geometry optimizations at the HF/6-31G(d) level of theory, followed by ESP charge generation from single point calculations with the HF/6-31G(d) method and RESP fitting using *antechamber* program of the Amber tools.[4] All cryptand structures have been solvated in periodic truncated octahedral boxes of chloroform or DMSO.[5] The final solvated systems contained around 3000 and 1500 molecules of chloroform or DMSO, respectively. The monovalent ion parameters used for simulations with encapsulated chloride ion are based on the non-bonded model parameterisations by Li et al.[6]

Following a steepest descent and conjugate gradient minimisation for 10000 steps, all simulations were carried out for at least 1 µs using integration time step intervals of 2 fs and applying constant pressure (NPT) molecular dynamics at 1 atm and 300 K using a Langevin dynamics. All bonds including hydrogens were constrained applying SHAKE algorithm. Electrostatic long-range interactions were treated with the Particle Mesh Ewald (PME) method and a 12 Å cut-off for non-bonded interactions.

All simulation parameters (topology and coordinate) necessary to reproduce MD simulations are available online on the Figshare repository (DOI: 10.6084/m9.figshare.19086995).

List of abbreviations

BArF	tetrakis[3,5-bis(trifluoromethyl)-phenyl]borate
CDI	*N,N'*-carbonyldiimidazole
CID	collision-induced dissociation
DCM	dichloromethane
DMSO	dimethyl sulfoxide
DMF	*N*,*N*-dimethylformamide
ESI	electronspray ionization
EtOAc	ethyl acetate
HRMS	high resolution mass spectrometry
MeCN	acetonitrile
MeOH	methanol
r.t.	room temperature
TBDMS	*tert*-butyldimethylsilyl
TEA	triethylamine
TEG	triethylene glycol
THF	tetrahydrofuran
TPP	tetraphenylphosphonium
TMA	tetramethylammonium

2. Synthesis and Characterization of Diol Building Blocks

*Overview of the synthesis of diols (**1**) and (**S9**)*

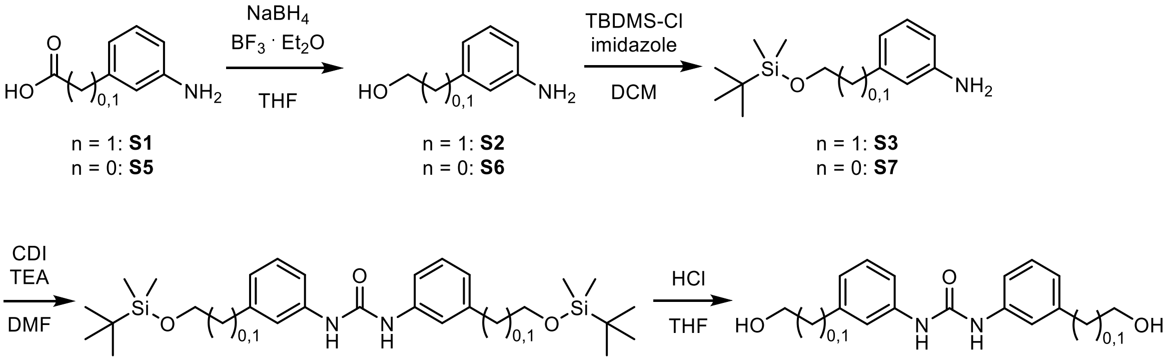

n = 1: **S1**, n = 0: **S5**; n = 1: **S2**, n = 0: **S6**; n = 1: **S3**, n = 0: **S7**; n = 1: **S4**, n = 0: **S8**; n = 1: **1**, n = 0: **S9**

*Synthesis of 1,3-bis(3-(2-hydroxyethyl)phenyl)urea (**1**)*

Synthesis of 2-(3-aminophenyl)ethan-1-ol (S2)

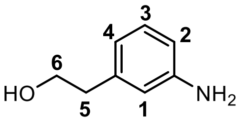

2-(3-Aminophenyl)acetic acid (**S1**) (5.00 g, 33.1 mmol, 1 equiv.) and $NaBH_4$ (1.89 g, 49.7 mmol, 1.5 equiv.) were suspended in 50 mL anhydrous THF under argon and cooled to 0 °C. $BF_3 \cdot Et_2O$ (6.3 mL, 7.06 g, 49.7 mmol, 1.5 equiv.) was added dropwise. The mixture was left stirring at room temperature for 20 h. After cooling to 0 °C, 50 mL 1 M NaOH solution (aq.) were added carefully. After filtration, THF was removed under vacuum. The product was extracted with EtOAc and the combined organic layers were washed with water and brine and dried over $MgSO_4$. Removal of the solvent under reduced pressure yielded the product as a yellow-brown solid (3.57 g, 26.1 mmol, 79%).

^{1}H NMR (400 MHz, DMSO-d$_6$): δ = 6.88 (t, ^3J = 7.64 Hz, 1 H, **3**), 6.41-6.31 (m, 3 H, **C*H**$_{arom.}$), 4.90 (s, 2 H, **-N*H*$_2$**), 4.56 (t, ^3J = 5.35 Hz, 1 H, **-O*H***), 3.53 (td, ^3J = 7.40, 5.23 Hz, 2 H, **6**), 2.55 (t, ^3J = 7.38 Hz, 2 H, **5**) ppm.

^{13}C NMR {^{1}H} (101 MHz, DMSO-d$_6$): 148.5, 139.8, 128.7, 116.6, 114.6, 111.7, 62.5, 39.5 ppm.

The received characterization data corresponds to literature[7].

Synthesis of 3-(2-((*tert*-butyldimethylsilyl)oxy)ethyl)aniline (S3)

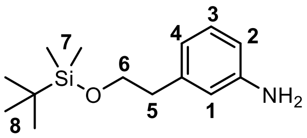

2-(3-Aminophenyl)ethan-1-ol (**S2**) (2.35 g, 17.1 mmol, 1 equiv.) was dissolved in 50 mL anhydrous DCM under argon and imidazole (3.49 g, 51.3 mmol, 3 equiv.) was added. After cooling the mixture to 0 °C, TBDMS-Cl (3.10 g, 20.5 mmol, 1.2 equiv.) was added. The reaction was left stirring at room temperature overnight. The organic layer was washed with sat. $NaHCO_3$ solution, water and brine, and dried over $MgSO_4$. The solvent was removed under reduced pressure and the pure product was obtained as a yellow oil (3.93 g, 15.7 mmol, 92%).

^{1}H NMR (400 MHz, DMSO-d$_6$): δ = 6.90 (t, ^{3}J = 7.34 Hz, 1 H, **3**), 6.41-6.32 (m, 3 H, **C*H*$_{arom.}$**), 4.91 (s, 2 H, **-N*H*$_2$**), 3.71 (t, ^{3}J = 7.21 Hz, 2 H, **6**), 2.59 (t, ^{3}J = 7.22 Hz, 2 H, **5**), 0.85 (s, 9 H, **8**), -0.01 (s, 6 H, **7**) ppm.

^{13}C NMR {^{1}H} (101 MHz, DMSO-d$_6$): δ = 148.7, 139.3, 128.8, 116.8, 114.8, 112.1, 64.4, 39.5, 26.1, 18.2, -5.1 ppm.

ESI-MS: m/z = 252.17790 [M+H]$^+$ (calculated: m/z = 252.17837).

Synthesis of 1,3-bis(3-(2-((*tert*-butyldimethylsilyl)oxy)ethyl)phenyl)urea (S4)

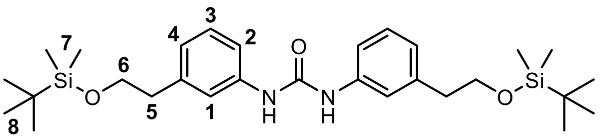

3-(2-((*Tert*-butyldimethylsilyl)oxy)-ethyl)aniline (**S3**) (3.00 g, 11.9 mmol, 2 equiv.) and *N,N'*-carbonyldiimidazole (968 mg, 5.98 mmol, 1 equiv.) were dissolved in 25 mL anhydrous DMF under argon. Triethylamine (4.2 mL, 3.02 g, 29.9 mmol, 5 equiv.) was added dropwise. The mixture was left stirring at room temperature for 3 days. The solvent was removed under reduced pressure, and the residue was recrystallized from EtOH. The pure product was obtained as a yellow solid (2.47 g, 4.67 mmol, 78%).

^{1}H NMR (400 MHz, DMSO-d$_6$): δ = 8.57 (s, 2 H, **-N*H***), 7.32 – 7.30 (m, 2 H, **1**), 7.28 – 7.25 (m, 2 H, **C*H*$_{arom.}$**), 7.16 (t, ^{3}J = 7.76 Hz, 2 H, **3**), 6.84 – 6.80 (m, 2 H, **C*H*$_{arom.}$**), 3.76 (t, ^{3}J = 6.81 Hz, 4 H, **6**), 2.71 (t, ^{3}J = 6.80 Hz, 4 H, **5**), 0.83 (s, 18 H, **8**), -0.04 (s, 12 H, **7**) ppm.

^{13}C NMR {^{1}H} (101 MHz, DMSO-d$_6$): δ = 152.4, 139.5, 139.5, 128.4, 122.6, 118.7, 115.9, 63.9, 39.7, 25.8, 17.9, -5.4 ppm.

ESI-MS: m/z = 551.31120 [M+Na]$^+$ (calculated: m/z = 551.31012), 567.28509 [M+K]$^+$ (calculated: m/z = 567.28406).

Synthesis of 1,3-bis(3-(2-hydroxyethyl)phenyl)urea (1)

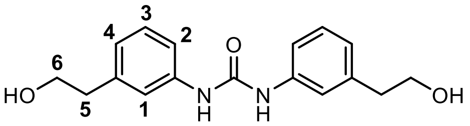

1,3-Bis(3-(2-((*tert*-butyldimethylsilyl)oxy)ethyl)phenyl)urea (**S4**) (559 mg, 1.06 mmol, 1 equiv.) was dissolved in 10 mL THF and 2.5 mL 5 M HCl were added dropwise. The reaction mixture was left stirring at room temperature for 45 minutes and further heated to 60 °C for 1.5 hours. All volatiles were removed under reduced pressure, and the crude product was recrystallized from MeOH. The pure compound was obtained as a colorless solid (342 mg, 1.06 mmol, quant.).

^{1}H NMR (400 MHz, DMSO-d$_6$): δ = 8.61 (s, 2 H, **-N*H***), 7.32 (s, 2 H, **1**), 7.28 (d, ^{3}J = 9.13 Hz, 2 H, **C*H*$_{arom.}$**), 7.17 (t, ^{3}J = 7.77 Hz, 2 H, **3**), 6.82 (d, ^{3}J = 7.58 Hz, 2 H, **C*H*$_{arom.}$**), 4.66 (s$_{br}$, 2 H, **-O*H***), 3.60 (t, ^{3}J = 7.10 Hz, 4 H, **6**), 2.69 (t, ^{3}J = 7.08 Hz, 4 H, **5**) ppm.

^{13}C NMR {^{1}H} (101 MHz, DMSO-d$_6$): δ = 152.6, 140.2, 139.6, 128.6, 122.5, 118.7, 115.9, 62.3, 39.3 ppm.

ESI-MS: m/z = 323.13676 [M+Na]$^+$ (calculated: m/z = 323.13716), 623.28639 [2M+Na]$^+$ (calculated: m/z = 623.28455).

*Synthesis of 1,3-bis(3-(hydroxymethyl)phenyl)urea (**S9**)*

Synthesis of (3-aminophenyl)methanol (S6)

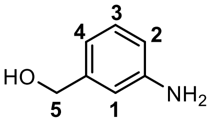

3-Aminobenzoic acid (**S5**) (5.04 g, 36.8 mmol, 1 equiv.) was suspended in 30 mL anhydrous THF under argon and cooled to 0 °C. $LiAlH_4$ solution (1 M in THF, 44 mL, 44.0 mmol, 1.2 equiv.) was added dropwise. The mixture was warmed up to room temperature and left stirring for 1 h. Afterwards, the mixture was heated to 60 °C for 3 h. After cooling to room temperature, it was quenched with 110 mL cooled water. The aqueous layer was extracted with chloroform and the combined organic layers were washed with brine and dried over $MgSO_4$. Removal of the solvent under reduced pressure yielded the product as a beige solid (2.55 g, 20.7 mmol, 56%).

^{1}H NMR (400 MHz, $CDCl_3$): δ = 7.15 (t, ^3J = 7.72 Hz, 1 H, **3**), 6.77 – 6.70 (m, 2 H, **-C*H*$_{arom.}$**), 6.64 – 6.60 (m, 1 H, **-C*H*$_{arom.}$**), 4.61 (s, 2 H, **5**) ppm.

The observed ^{13}C NMR data corresponds to the literature[8].

Synthesis of 3-(((*tert*-butyldimethylsilyl)oxy)methyl)aniline (S7)

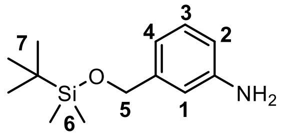

Compound (**S7**) was synthesized according to the same procedure as compound (**S3**) and was obtained as a yellow liquid (4.85 g, 20.5 mmol, quant.).

^{1}H NMR (400 MHz, $CDCl_3$): δ = 7.11 (t, ^3J = 7.65 Hz, 1 H, **3**), 6.72 – 6.66 (m, 2 H, **-C*H*$_{arom.}$**), 6.58 – 6.55 (m, 1 H, **-C*H*$_{arom.}$**), 4.66 (s, 2 H, **5**), 3.64 (s_{br}, 2 H, **-N*H$_2$***), 0.94 (s, 9 H, **7**), 0.10 (s, 6 H, **6**) ppm.

The observed ^{13}C NMR data corresponds to the literature[9].

Synthesis of 1,3-bis(3-(((*tert*-butylsilyldimethyl)oxy)methyl)phenyl)urea (S8)

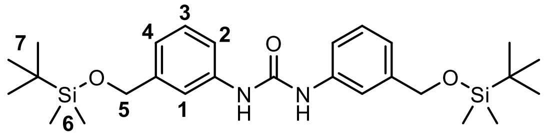

Compound (**S8**) was synthesized similarly to compound (**S4**) with some deviations. The reaction mixture was left stirring at room temperature for 7 days. The crude compound was purified by flash column chromatography (DCM/MeOH, 50:1). The pure product was obtained as a colorless solid (2.05 g, 4.10 mmol, 48%).

^{1}H NMR (400 MHz, DMSO-d$_6$): δ = 8.62 (s, 2 H, **-N*H***), 7.41 (s, 2 H, **1**), 7.36 – 7.32 (m, 2 H, **-C*H*$_{arom.}$**), 7.23 (t, ^3J = 7.77 Hz, 2 H, **3**), 6.92 – 6.88 (m, 2 H, **-C*H*$_{arom.}$**), 4.67 (s, 4 H, **5**), 0.91 (s, 18 H, **7**), 0.09 (s, 12 H, **6**) ppm.

^{13}C NMR {^{1}H} (101 MHz, DMSO- d$_6$): 152.4, 141.8, 139.6, 128.5, 119.5, 116.7, 115.8, 64.3, 25.8, 18.0, -5.3 ppm.

ESI-MS: m/z = 523.27857 [M+Na]$_+$ (calculated: m/z = 523.27827).

Synthesis of 1,3-bis(3-(hydroxymethyl)phenyl)urea (S9)

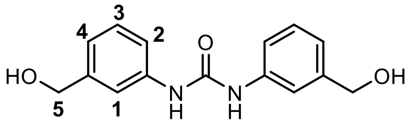

Compound (**S9**) was synthesized according to the same procedure as compound (**1**). The pure product was obtained as a colorless solid (995 mg, 3.66 mmol, quant.).

^{1}H NMR (400 MHz, DMSO- d$_6$): δ = 8.60 (s, 2 H, **-N*H***), 7.43 (s, 2 H, **1**), 7.31 (d, ^3J = 8.94 Hz, 2 H, **-C*H*$_{arom.}$**), 7.21 (t, ^3J = 7.77 Hz, 2 H, **3**), 6.93 – 6.89 (m, 2 H, **-C*H*$_{arom.}$**), 5.15 (t, ^3J = 5.77 Hz, 2 H, **-O*H***), 4.46 (d, ^3J = 5.82 Hz, 4 H, **5**) ppm.

^{13}C NMR {^{1}H} (101 MHz, DMSO-d$_6$): 152.5, 143.2, 139.6, 128.4, 119.9, 116.4, 116.2, 62.9 ppm.

ESI-MS: m/z = 295.10610 [M+Na]$^+$ (calculated: m/z = 295.10531), 567.22388 [2M+Na]$^+$ (calculated: m/z = 567.22141), 839.34268 [3M+Na]$^+$ (calculated: m/z = 839.33750).

*Overview of the synthesis of diols (**2**) and (**3**)*

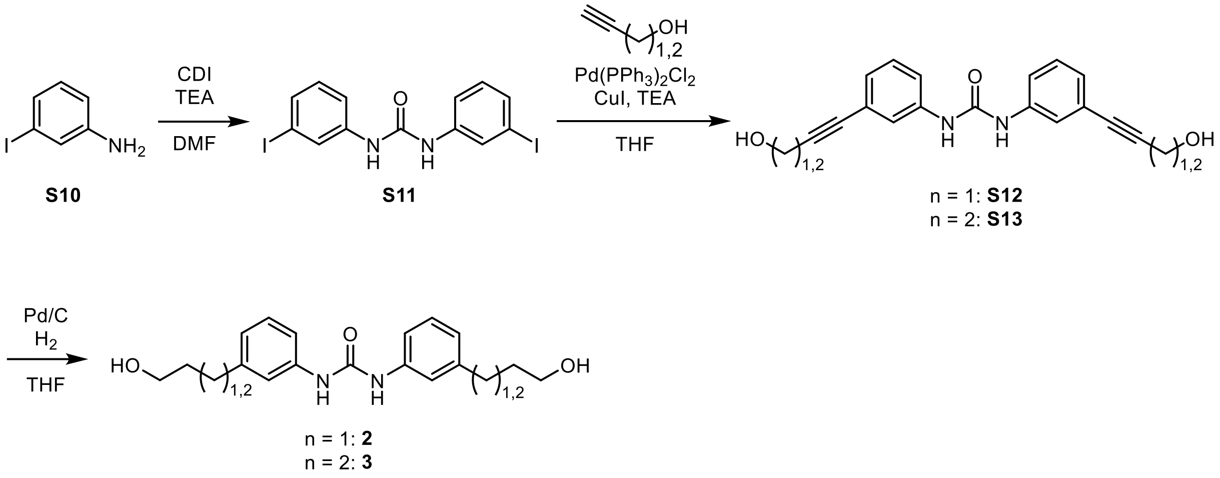

*Synthesis of 1,3-bis(3-(3-hydroxypropyl)phenyl)urea (**2**)*

Synthesis of 1,3-bis(3-iodophenyl)urea (S11)

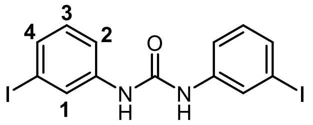

3-Iodoaniline (**S10**) (10.0 g, 5.49 mL, 45.7 mmol, 2 eq.) and *N,N'*-carbonyldiimidazole (3.70 g, 22.8 mmol, 1 eq.) were dissolved in anhydrous DMF under argon and TEA (11.5 g, 15.9 mL, 114 mmol, 5 eq.) was added dropwise. The solution was left stirring at room temperature for 3 days. Water was added to the reaction mixture and the clean product precipitated from the solution. The product was filtered off and the colorless solid was washed with water (6.50 g, 14.0 mmol, 67%).

^{1}H NMR (400 MHz, DMSO-d$_6$): δ = 8.85 (s, 2 H, **NH**), 8.00 (s, 2 H, **1**), 7.37 – 7.28 (m, 4 H, **2/4**), 7.08 (t, ^3J = 8.00 Hz, 2 H, **3**) ppm.

^{13}C NMR {^{1}H} (101 MHz, DMSO-d$_6$): 152.1, 140.9, 130.8, 130.5, 126.4, 117.7, 94.8 ppm.

ESI-MS: m/z = 462.88142 [M-H]$^-$ (calculated: m/z = 462.88044), 498.85757 [M+Cl]$^-$ (calculated: m/z = 498.85712).

Synthesis of 1,3-bis(3-(3-hydroxyprop-1-yn-1-yl)phenyl)urea (S12)

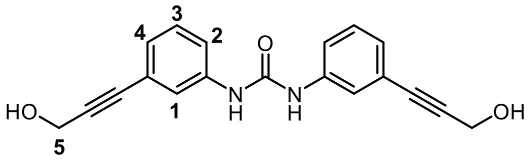

1,3-Bis(3-iodophenyl)urea (**S11**) (500 mg, 1.08 mmol, 1 eq.) and $Pd(PPh_3)_2Cl_2$ (151 mg, 0.216 mmol, 20 mol-%) were dissolved in 10 mL degassed THF. 5 mL TEA and 3-propargyl-1-ol (157 µL, 151 mg, 2.70 mmol, 2.5 eq.) were added. The reaction mixture was left stirring at room temperature for 5 minutes. Copper(I) iodide (82.1 mg, 0.432 mmol, 40 mol-%) was added. After 18 h of stirring at room temperature, the mixture was filtered through a silica plug using DCM/MeOH (8:2) as eluent. The crude product was recrystallized from MeOH to yield the pure product as a colorless solid (127 mg, 0.396 mmol, 37%).

^{1}H NMR (400 MHz, DMSO-d$_6$): δ = 8.83 (s, 2 H, N***H***), 7.66 – 7.61 (m, 2 H, **1**), 7.40 – 7.33 (m, 2 H, **C*H*arom.**), 7.28 (t, ^{3}J = 7.88 Hz, 2 H, **3**), 7.05 – 7.00 (m, 2 H, **C*H*arom.**), 5.35 (t, ^{3}J = 5.96 Hz, 2 H, **-OH**), 4.30 (t, ^{3}J = 5.86 Hz, 4 H, **5**) ppm.

^{13}C NMR {^{1}H} (101 MHz, DMSO-d$_6$): 152.5, 139.8, 129.2, 124.8, 122.8, 120.8, 118.5, 89.6, 83.7, 49.5 ppm.

ESI-MS: m/z = 382.10377 $[M+NO_3]^-$ (calculated: m/z = 382.10391).

Synthesis of 1,3-bis(3-(3-hydroxypropyl)phenyl)urea (2)

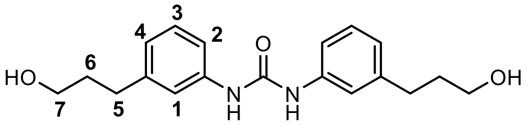

1,3-Bis(3-(3-hydroxyprop-1-yn-1-yl)phenyl)urea (**S12**) (127 mg, 0.396 mmol, 1 equiv.) was dissolved in 15 mL THF and palladium on charcoal (10 wt-%, 63.1 mg, 59.5 µmol, 15 mol-%) was added. The reaction mixture was left stirring (100 rpm) at room temperature under a hydrogen atmosphere. After 18 hours, the suspension was filtered through a syringe filter to remove the heterogeneous catalyst. The crude product was purified by column chromatography (DCM/MeOH, 9:1). After removal of the solvent under reduced pressure, the pure product was obtained as a beige solid (70.3 mg, 0.214 mmol, 54%).

^{1}H NMR (400 MHz, DMSO-d$_6$): δ = 8.57 (s, 2 H, N***H***), 7.33 (s, 2 H, **1**), 7.24 – 7.12 (m, 4 H, **C*H*** ***arom.***), 6.80 (d, ^{3}J = 7.29 Hz, 2 H, **C*H*** ***arom.***), 4.48 (t, ^{3}J = 5.15 Hz, 2 H, **-O*H***), 3.42 (q, ^{3}J = 6.39, 6.40 Hz, 4 H, **7**), 2.61 – 2.52 (m, 4 H, **5**), 1.74 – 1.66 (m, 4 H, **6**) ppm.

^{13}C NMR {^{1}H} (101 MHz, DMSO-d$_6$): 152.51, 142.82, 139.67, 128.64, 121.89, 118.03, 115.57, 60.15, 34.36, 31.84 ppm.

ESI-MS: m/z = 351.16772 $[M+Na]^+$ (calculated: m/z = 351.16846).

*Synthesis of 1,3-bis(3-(4-hydroxybutyl)phenyl)urea (**3**)*

Synthesis of 1,3-bis(3-(4-hydroxybut-1-yn-1-yl)phenyl)urea (S13)

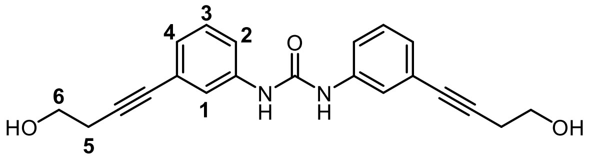

Compound (**S13**) was synthesized according to the same procedure as compound (**S12**). After 18 h of stirring at room temperature, the product precipitated. The product was filtered off and the precipitate was washed with THF and Et_2O. The crude product was recrystallized from THF to yield the pure product as a colorless solid (943 mg, 2.56 mmol, 40%).

^{1}H NMR (400 MHz, DMSO-d$_6$): δ = 8.76 (s, 2 H, **N*H***), 7.61 (s, 2 H, **1**), 7.39 – 7.13 (m, 4 H, **C*H*arom.**), 6.99 (d, ^3J = 7.33 Hz, 2 H, **C*H*arom.**), 4.91 (t, ^3J = 5.53 Hz, 2 H, **-OH**), 3.58 (q, ^3J = 6.57 Hz, 4 H, **6**), 2.55 (t, ^3J = 6.78 Hz, 4 H, **5**).

^{13}C NMR {^{1}H} (101 MHz, DMSO-d$_6$): δ = 152.4, 139.7, 129.0, 124.8, 123.6, 120.8, 118.0, 88.2, 81.1, 59.8, 23.2 ppm.

ESI-MS: m/z = 371.13789 [M+Na]$^+$ (calculated: m/z = 371.13716), 719.28527 [2M+Na]$^+$ (calculated: m/z = 719.28455).

Synthesis of 1,3-bis(3-(4-hydroxybutyl)phenyl)urea (3)

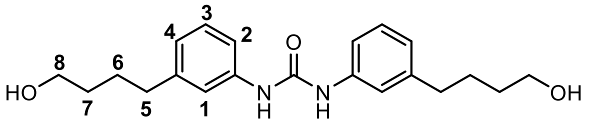

Compound (**3**) was synthesized according to the same procedure as compound (**2**). The crude product was purified by column chromatography (EtOAc). The pure product was obtained as a colorless solid (604 mg, 1.70 mmol, 66%).

^{1}H NMR (400 MHz, DMSO-d$_6$): δ = 8.55 (s, 2 H **N*H***), 7.36 - 7.28 (m, 2 H, **1**), 7.24 – 7.10 (m, 4 H, **C*H*arom.**), 6.82 – 6.76 (m, 2 H, **C*H*arom.**), 4.36 (t, ^3J = 5.19 Hz, 2 H, **-O*H***), 3.41 (td, ^3J = 6.49, 5.15 Hz, 4 H, **8**), 2.54 (t, ^3J = 7.67 Hz, 4 H, **5**), 1.64 – 1.52 (m, 4 H, **-C*H*$_2$-**), 1.50 – 1.39 (m, 4 H, **-C*H*$_2$-**) ppm.

^{13}C NMR {^{1}H} (101 MHz, DMSO-d$_6$): δ = 152.5, 142.9, 139.6, 128.6, 121.9, 118.0, 115.6, 60.5, 35.2, 32.1, 27.4 ppm.

ESI-MS: m/z = 379.19922 [M+Na]$^+$ (calculated: m/z = 379.19976).

3. Synthesis and Characterization of Chloride-Templated Cryptates

3.1 General Procedures

Drying of starting materials

Diols (**1**, **2**, **3**, **S9**), CsCl and salts used for titration experiments (TPPCl, TPPBr, TPPI, $TMANO_3$) were filled in screw threaded glass vials and dried at 80 °C under vacuum over phosphorus pentoxide for three days. Tetraphenylphosphonium chloride (TPPCl) used as template in self-assembly experiments was dissolved in anhydrous acetonitrile, and trimethyl orthoacetate (7 equiv.) and TFA (1.2 equiv.) were added. After stirring for 1 hour, the volatiles were removed under reduced pressure to obtain the dry salt. Triethylene glycol, trimethyl orthoacetate and trimethyl orthofomate were stored over 3 Å MS and aluminum oxide. 2,3,4,5,6-pentafluorobenzenethiol was used as purchased. The solvents were dried over 3 Å MS for at least 24 hours.

General procedure (A) for the synthesis of orthoacetate-based Chloride Cryptates

The reaction was performed under nitrogen atmosphere. Diol (**1**, **2**, **3**) (90 µmol, 3 equiv.) and TPPCl (11.3 mg, 30 µmol, 1 equiv.) were dissolved in 550 µL dry deuterated DMSO and 2.75 mL dry $CDCl_3$. Trimethyl orthoacetate (8.0 µL, 7.6 mg, 63 µmol, 2.1 equiv.) and 2,3,4,5,6-pentafluorobenzenethiol (2.0 µL, 3.0 mg, 15 µmol, 0.5 equiv.) were added. To facilitate the slow removal of emerging methanol during the reaction, 5 Å molecular sieves (around 1.2 g) were admixed to the reaction vessel. The reaction progress was monitored by ^{1}H NMR spectroscopy. After disappearance of all signals corresponding to -*OMe* groups (**[Cl^-⊂*o*-Me_2-ur-C2]PPh_4^+**: 3 h, **[Cl^-⊂*o*-Me_2-ur-C3]PPh_4^+**: 18 h, **[Cl^-⊂*o*-Me_2-ur-C4]PPh_4^+**: 18 h), the reaction mixture was quenched with TEA (41.7 µL, 30.3 mg, 300 µmol, 10 eq.) and filtered to remove residual molecular sieves. The product was precipitated by the addition of 10 mL dry diethyl ether. After washing with diethyl ether, the pure product was dried under high vacuum.

General procedure (B) for the synthesis of orthoformate-based Chloride Cryptates

The reaction was performed under nitrogen atmosphere. Diol (**1**, **2**, **3**) (45.0 µmol, 3 equiv.) and TPPCl (5.6 mg, 15.0 µmol, 1 equiv.) were dissolved in 275 µL dry deuterated DMSO and 1.38 mL dry $CDCl_3$. Trimethyl orthoformate (3.5 µL, 3.3 mg, 31.5 µmol, 2.1 equiv.) and 2,3,4,5,6-pentafluorobenzenethiol (1.0 µL, 1.5 mg, 7.50 µmol, 0.5 equiv.) were added. To facilitate the slow removal of emerging methanol during the reaction, 5 Å molecular sieves (around 700 mg) were admixed to the reaction vessel. The reaction progress was monitored by ^{1}H NMR spectroscopy. After one day, an additional amount of 2,3,4,5,6-pentafluorobenzenethiol (1.0 µL, 1.5 mg, 7.50 µmol, 0.5 equiv.) was added. After one additional day, when using diol (**3**) disappearance of all signals corresponding to -*OMe* groups indicated completion of the reaction and the reaction mixture was treated in the same way as described in General procedure A. Even after longer reaction times, no full conversion could be detected for experiments using Diol (**1**) and (**2**).

General procedure (C) for the synthesis of heteroleptic orthoacetate Cryptates

In each experiment, two diols in different ratios (**S9**/**1**: 1:1, **1**/**2**: 1:2, 1:1, 2:1) were utilized. For the experiment, General procedure **A** was followed. After one day, aliquots (10 µL) of the reaction mixture were taken and diluted with 1 mL $CHCl_3$ and 1 mL acetonitrile. This solution was immediately used for ESI-CID-MS measurements.

General procedure (D) for the synthesis of Cryptands (chloride removal)

About 30 mg of corresponding cryptate were loaded into a screw capped vial. 100 µL TEA (to prevent undesired hydrolysis) and 4 mL dry MeOH were added and the suspension was stirred for 7 hours. The solution was removed and the TEA/MeOH mixture was added a second time. After stirring overnight, the solution was removed and the product was dried in vacuum. The cryptands were obtained in quantitative yields.

3.2 Characterization of Cryptates

*Characterization of **[Cl⁻⊂o-Me$_2$-ur-C2]PPh$_4^+$** and **o-Me$_2$-ur-C2***

[Cl⁻⊂*o*-Me$_2$-ur-C2]PPh$_4^+$

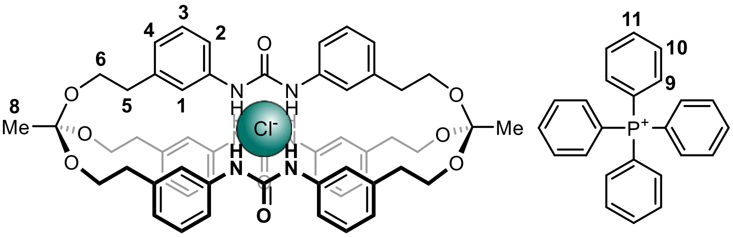

Yield: 21.9 mg (16.5 µmol, 55%)

^{1}H NMR (400 MHz, DMSO-*d$_6$*): δ = 8.73 – 8.49 (m, 6 H, N*H*), 8.03 – 7.91 (m, 4 H, **11**), 7.87 – 7.67 (m, 16 H, **9/10**), 7.40 - 7.00 (m, 18 H, C*H$_{arom.}$*), 6.89 – 6.68 (m, 6 H, C*H$_{arom.}$*), 3.66 – 3.40 (m, 12 H, **6**), 2.80 – 2.56 (m, 12 H, **5**), 1.40 – 1.20 (m, 6 H, **8**) ppm.

^{13}C NMR {^{1}H} (101 MHz, DMSO-*d$_6$*): δ = 152.7, 139.5, 135.4, 134.6, 134.5, 130.5, 130.4, 128.6, 122.3, 118.1, 117.2, 115.9, 113.9, 62.2, 35.8, 20.2, 15.2 ppm.

ESI-MS: m/z = 983.41152 [M-PPh$_4$]$^-$ (calculated: m/z = 983.41103).

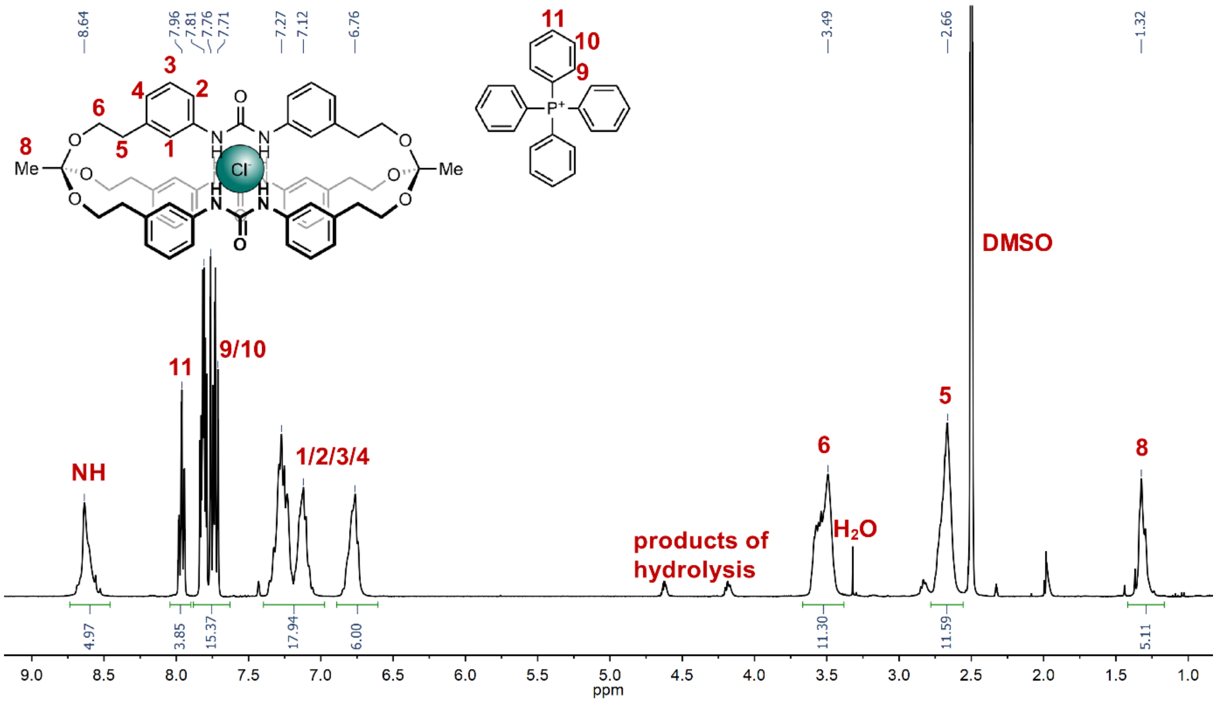

Figure S1: ^{1}H NMR (DMSO-d$_6$, 400 MHz, 293 K) spectrum of **[Cl⁻⊂*o*-Me$_2$-ur-C2]PPh$_4^+$**.

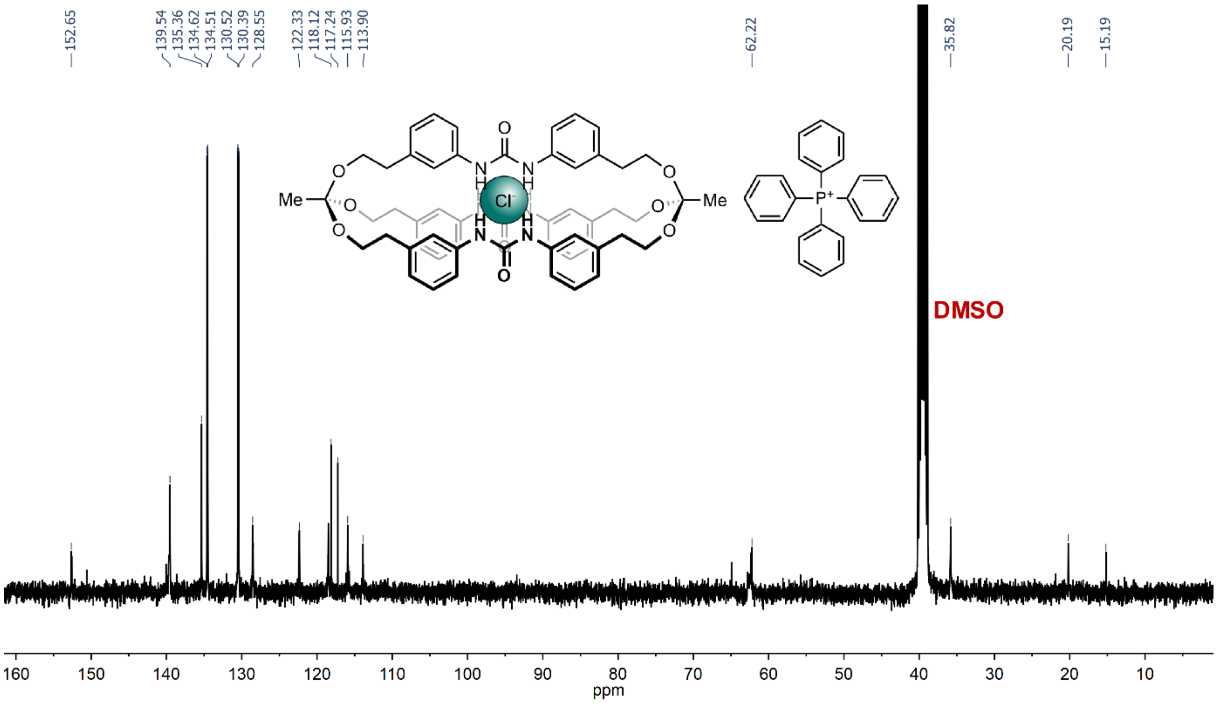

Figure S2: ^{13}C NMR (DMSO-d$_6$, 101 MHz, 293 K) spectrum of **[Cl$^-$⊂*o*-Me$_2$-ur-C2]PPh$_4^+$**.

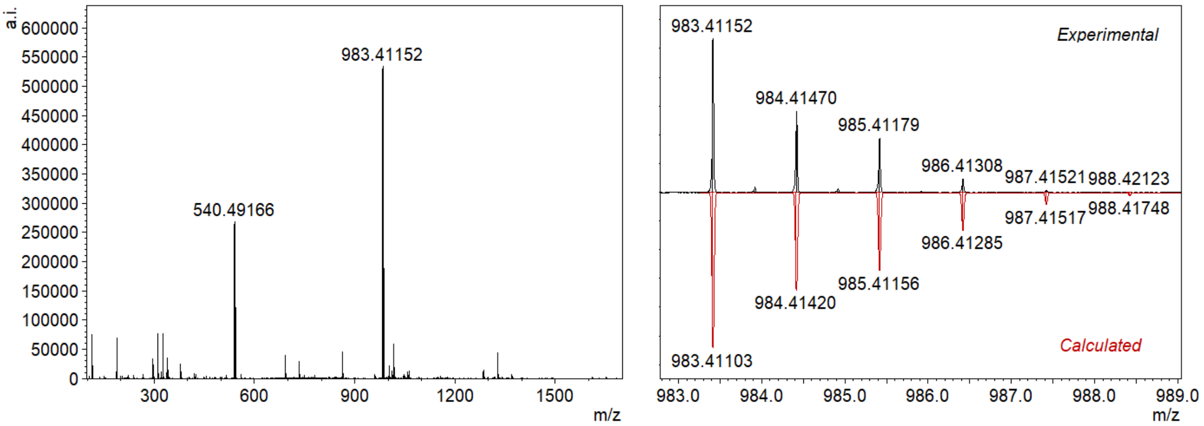

Figure S3: left: high resolution ESI-MS spectrum of **[Cl$^-$⊂*o*-Me$_2$-ur-C2]PPh$_4^+$** right: experimental (top) and simulated (bottom) isotopic pattern of product peak.

o-Me$_2$-ur-C2

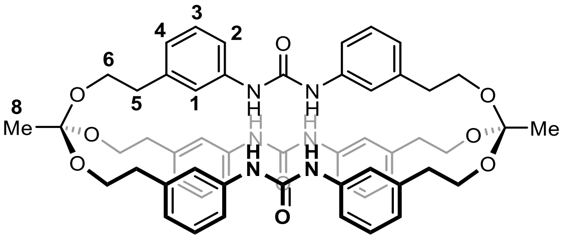

^{1}H NMR (400 MHz, DMSO-*d$_6$*): δ = 8.65 – 8.42 (m, 6 H, **N*H***), 7.44 – 7.01 (m, 18 H, **C*H*$_{arom.}$**), 6.87 – 6.72 (m, 6 H, **C*H*$_{arom.}$**), 3.66 – 3.41 (m, 12 H, **6**), 2.79 – 2.55 (m, 12 H, **5**), 1.41 – 1.21 (m, 6 H, **8**) ppm.

Each attempted recording of a ^{13}C NMR spectrum showed too much of the hydrolysed compound. For related ^{13}C NMR data, please refer to Figure S10 (***o*-Me₂-ur-C3**).

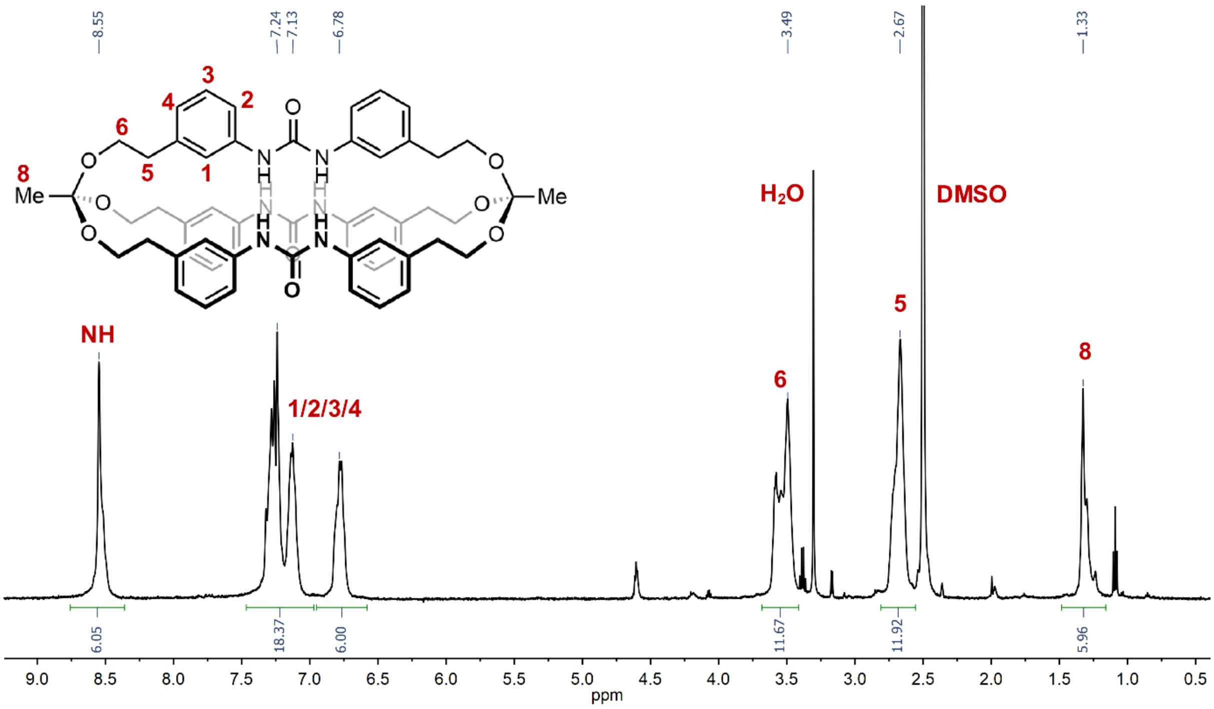

Figure S4: ^{1}H NMR (DMSO-d$_6$, 400 MHz, 293 K) spectrum of ***o*-Me$_2$-ur-C2.**

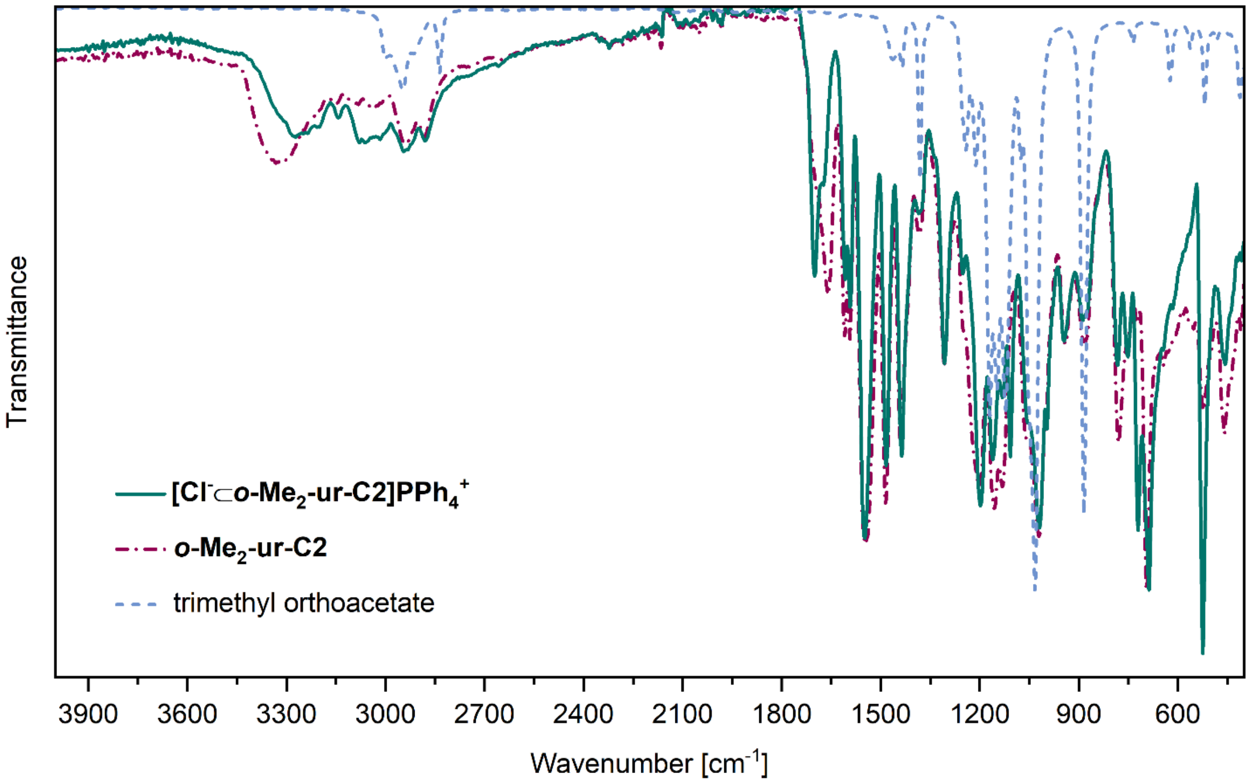

Figure S5: ATR-IR spectra of **[Cl$^-$⊂*o*-Me$_2$-ur-C2]PPh$_4^+$**, ***o*-Me$_2$-ur-C2** and trimethyl orthoacetate.

*Characterization of **[Cl⁻⊂o-Me$_2$-ur-C3]PPh$_4^+$** and **o-Me$_2$-ur-C3***

[Cl⁻⊂*o*-Me$_2$-ur-C3]PPh$_4^+$

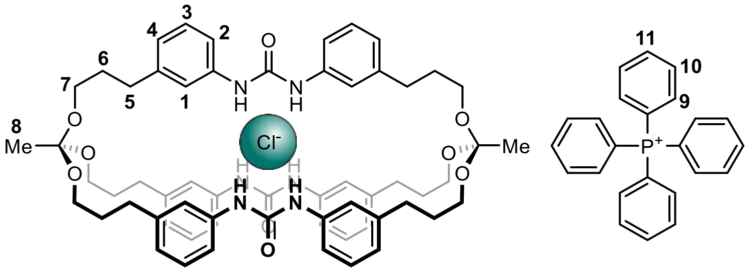

Yield: 20.4 mg (14.5 µmol, 48%)

^{1}H NMR (400 MHz, DMSO-*d$_6$*): δ = 8.64 (s$_{br}$, 6 H, N*H*), 7.99 – 7.92 (m, 4 H, **11**), 7.86 – 7.68 (m, 16 H, **9/10**), 7.41 - 7.27 (m, 6 H, **C*H*$_{arom.}$**), 7.27 – 7.04 (m, 12 H, **C*H*$_{arom.}$**), 6.81 – 6.69 (m, 6 H, **C*H*$_{arom.}$**), 3.45 – 3.36 (m, 12 H, **7**), 2.60 – 2.52 (m, 12 H, **5**), 1.72 (s$_{br}$, 12 H, **6**), 1.39 – 1.29 (m, 6 H, **8**) ppm.

^{13}C NMR {^{1}H} (101 MHz, DMSO-*d$_6$*): δ = 152.5, 142.3, 139.7, 135.4, 134.6, 134.5, 130.5, 130.4, 128.6, 121.8, 118.1, 117.9, 117.3, 115.6, 60.7, 60.5, 31.8, 31.0, 20.2 ppm.

ESI-MS: m/z = 1067.55576 [M-PPh$_4$]$^-$ (calculated: m/z = 1067.50493).

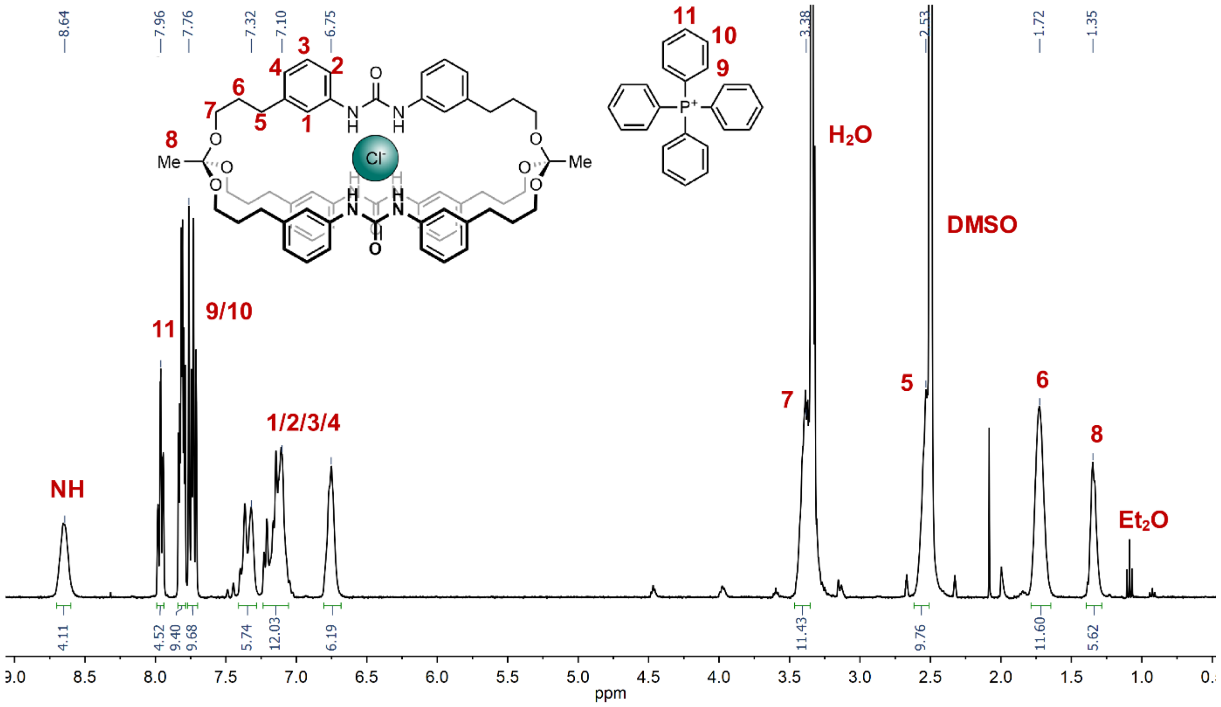

Figure S6: ^{1}H NMR (DMSO-d$_6$, 400 MHz, 293 K) spectrum of **[Cl⁻⊂*o*-Me$_2$-ur-C3]PPh$_4^+$**.

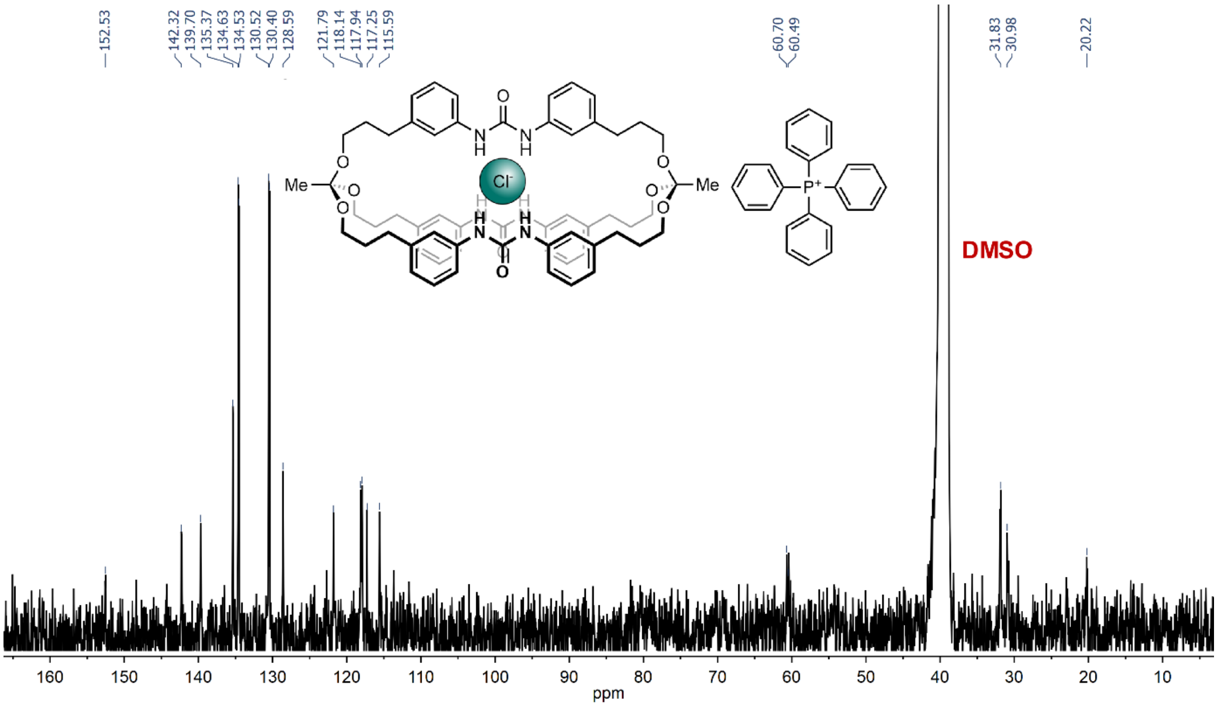

Figure S7: ^{13}C NMR (DMSO-d$_6$, 101 MHz, 293 K) spectrum of **[Cl$^-$⊂*o*-Me$_2$-ur-C3]PPh$_4^+$**.

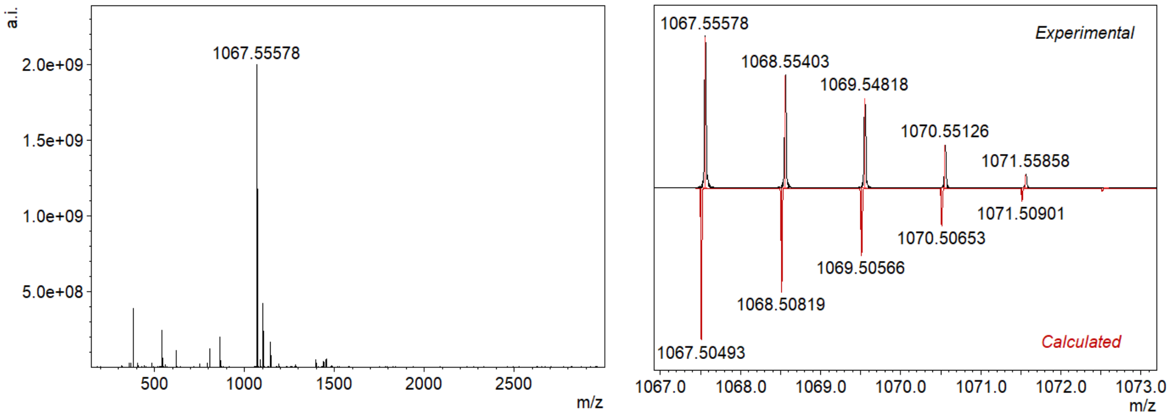

Figure S8: left: high resolution ESI-MS spectrum of **[Cl$^-$⊂*o*-Me$_2$-ur-C3]PPh$_4^+$** (solvent: acetonitrile), right: experimental (top) and simulated (bottom) isotopic pattern of product peak.

o-Me₂-ur-C3

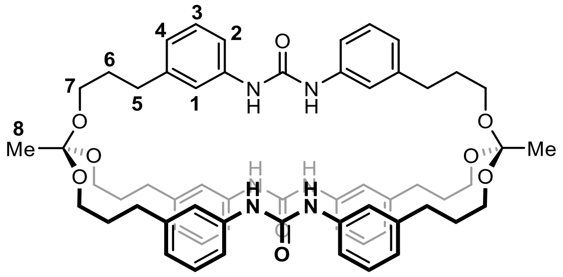

^{1}H NMR (400 MHz, DMSO-*d*$_6$): δ = 8.52 (s_{br}, 6 H, N*H*), 7.51 – 6.97 (m, 18 H, C*H$_{arom.}$*), 6.87 – 6.62 (m, 6 H, C*H$_{arom.}$*), 3.40 (s_{br}, 12 H, **7**), 2.55 (s_{br}, 12 H, **5**), 1.74 (s_{br}, 12 H, **6**), 1.36 (s_{br}, 6 H, **8**) ppm.

^{13}C NMR {^{1}H} (101 MHz, DMSO-*d*$_6$): δ = 152.4, 142.7, 142.2, 139.6, 128.5, 121.8, 118.0, 115.7, 113.7, 60.6, 60.4, 60.1, 34.2, 31.9, 30.9, 20.2 ppm.

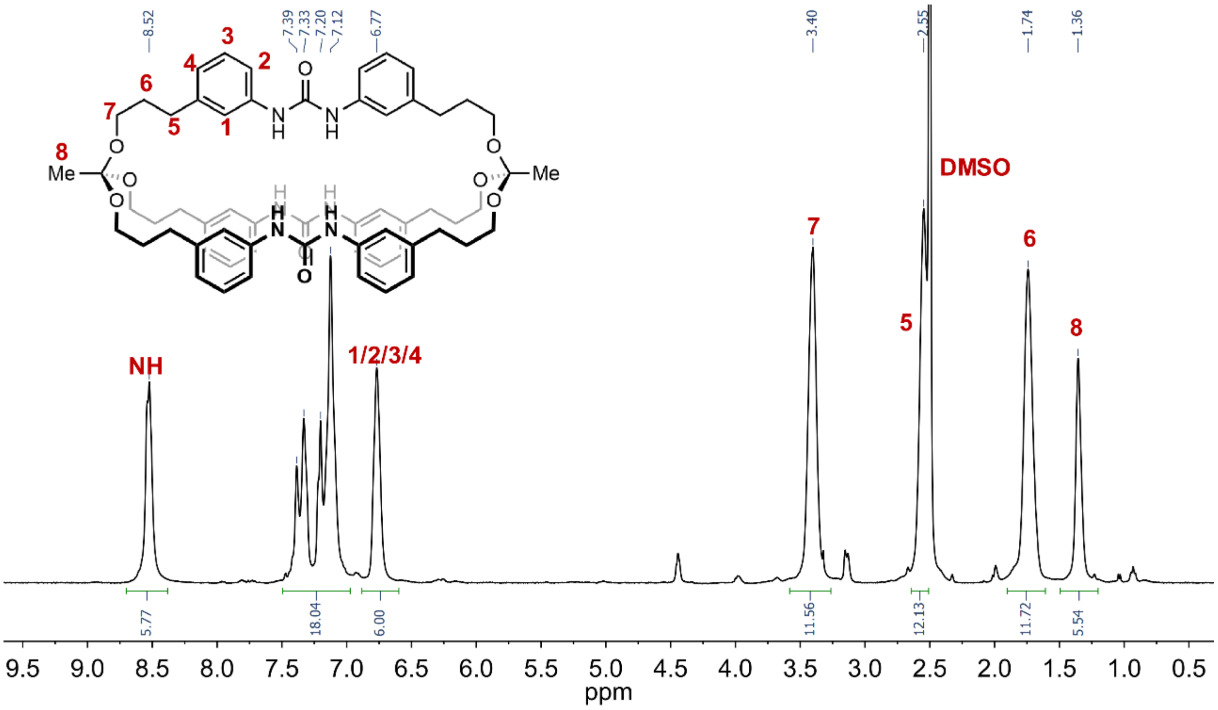

Figure S9: ^{1}H NMR (DMSO-d$_6$, 400 MHz, 293 K) spectrum of ***o*-Me₂-ur-C3**.

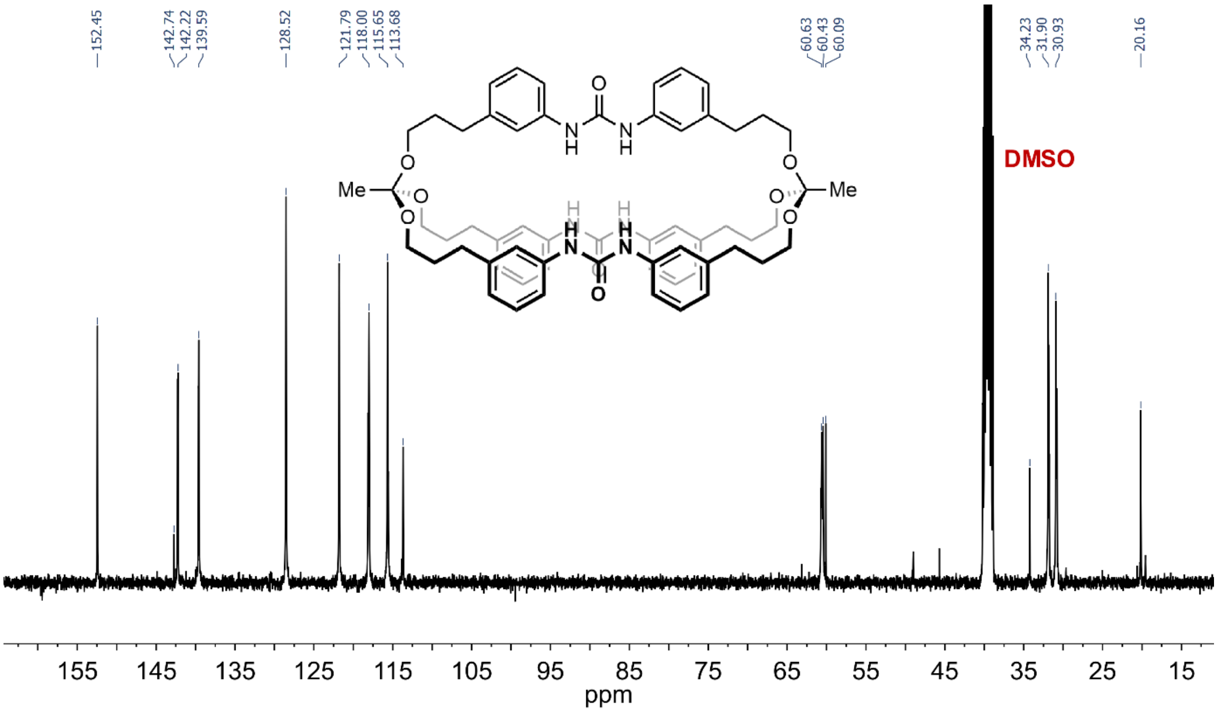

Figure S10: ^{13}C NMR (DMSO-d$_6$, 101 MHz, 293 K) spectrum of ***o*-Me$_2$-ur-C3**.

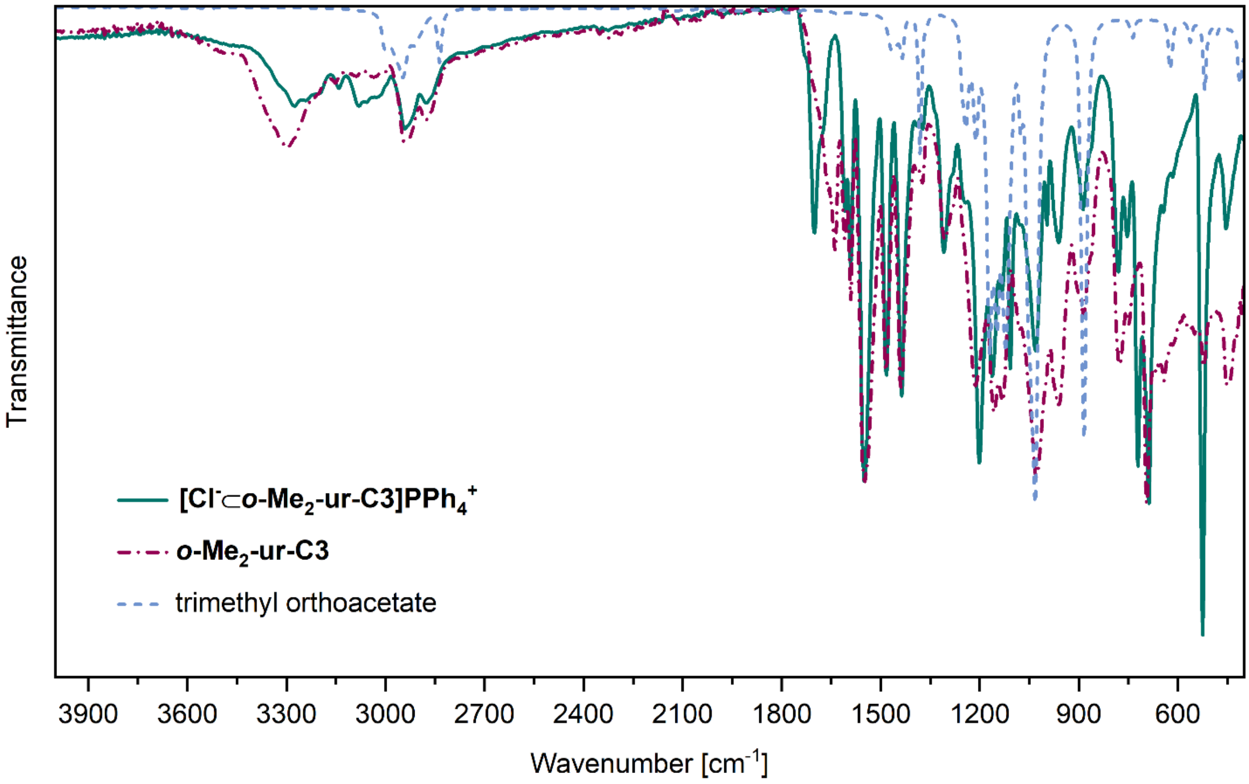

Figure S11: ATR-IR spectra of **[Cl$^-$⊂*o*-Me$_2$-ur-C3]PPh$_4^+$**, ***o*-Me$_2$-ur-C3** and trimethyl orthoacetate.

Characterization of ***[Cl⁻⊂o-Me₂-ur-C4]PPh₄⁺*** *and* ***o-Me₂-ur-C4***

[Cl⁻⊂*o*-Me₂-ur-C4]PPh₄⁺

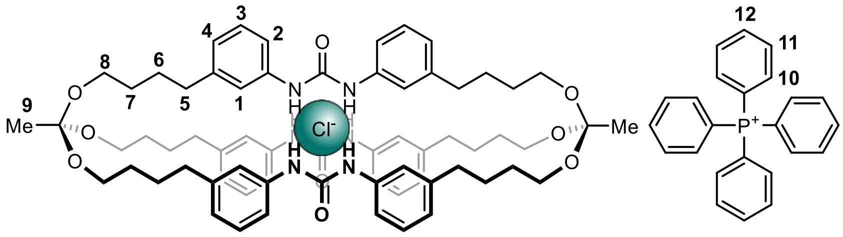

Yield: 10.3 mg (6.90 µmol, 23%) Note: isolated yield not representative for self-assembly due to partial precipitation (Et_2O).

^{1}H NMR (400 MHz, DMSO-*d₆*): δ = 8.81 (s$_{br}$, 6 H, **N*H***), 8.03 – 7.91 (m, 4 H, **12**), 7.86 – 7.66 (m, 16 H, **10/11**), 7.40 – 7.02 (m, 18 H, **C*H*$_{arom.}$**), 6.80 – 6.63 (m, 6 H, **C*H*$_{arom.}$**), 3.48 – 3.23 (m, 12 H, **8**), 2.59 – 2.49 (m, 12 H, **5**), 1.66 – 1.37 (m, 24 H, **6/7**), 1.32 (s$_{br}$, 6 H, **9**) ppm.

^{13}C NMR {^{1}H} (101 MHz, DMSO-*d₆*): δ = 152.5, 142.7, 139.7, 135.3, 134.5, 130.5, 130.4, 128.5, 121.7, 118.1, 117.9, 117.2, 115.5, 113.6, 64.9, 63.6, 61.0, 60.5, 35.0, 32.1, 28.9, 27.7, 27.4, 20.1, 15.2 ppm.

ESI-MS: m/z = 1169.60109 [M+H_2O-PPh_4]$^-$ (calculated: m/z = 1169.60940).

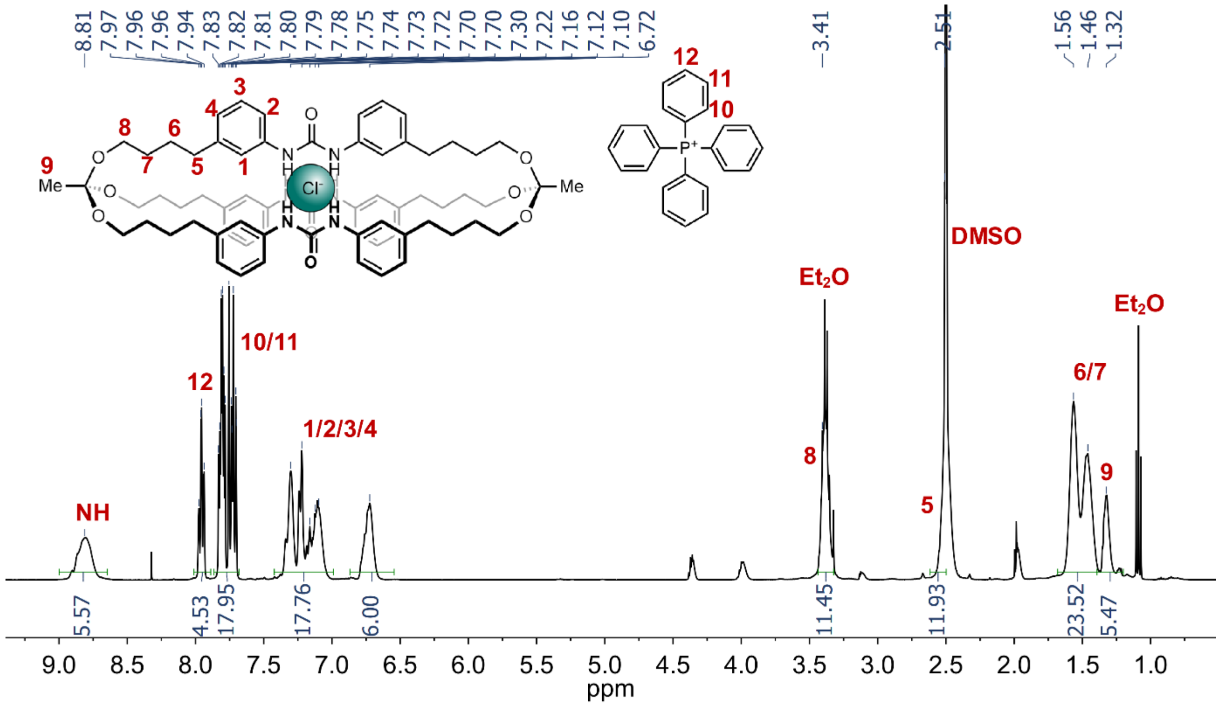

Figure S12: ^{1}H NMR (DMSO-d₆, 400 MHz, 293 K) spectrum of **[Cl⁻⊂*o*-Me₂-ur-C4]PPh₄⁺**.

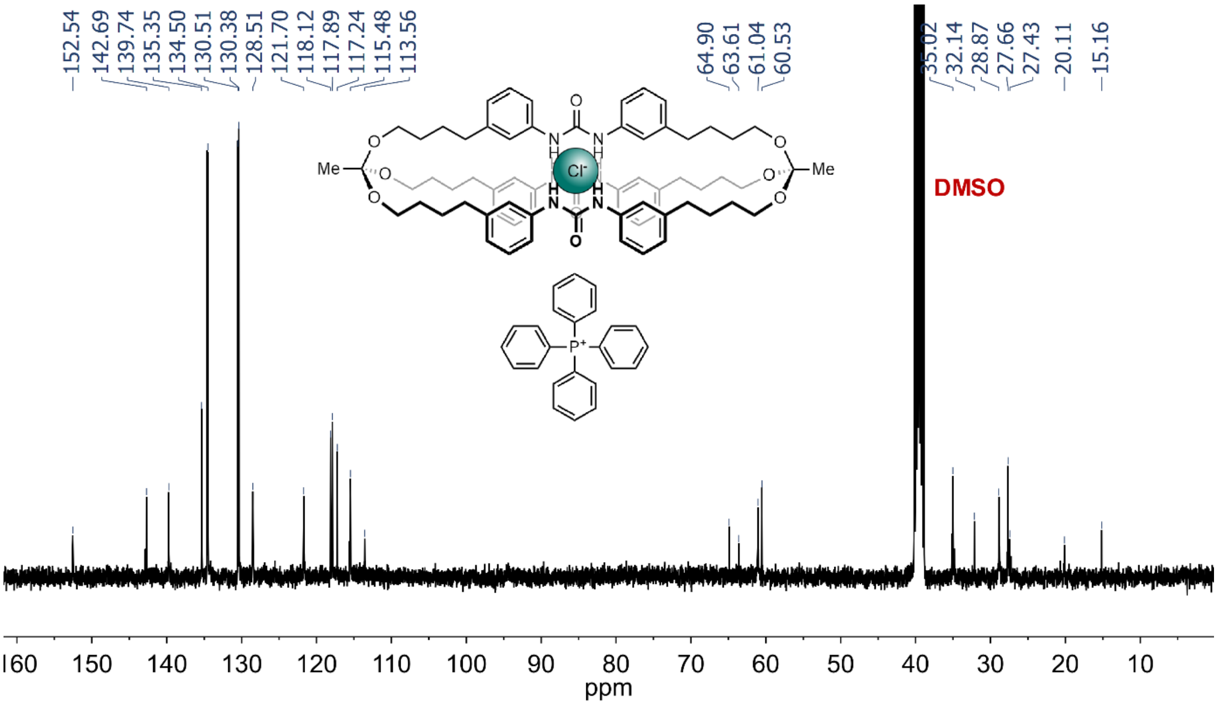

Figure S13: ^{13}C NMR (DMSO-d$_6$, 101 MHz, 293 K) spectrum of **[Cl$^-$⊂*o*-Me$_2$-ur-C4]PPh$_4$$^+$**.

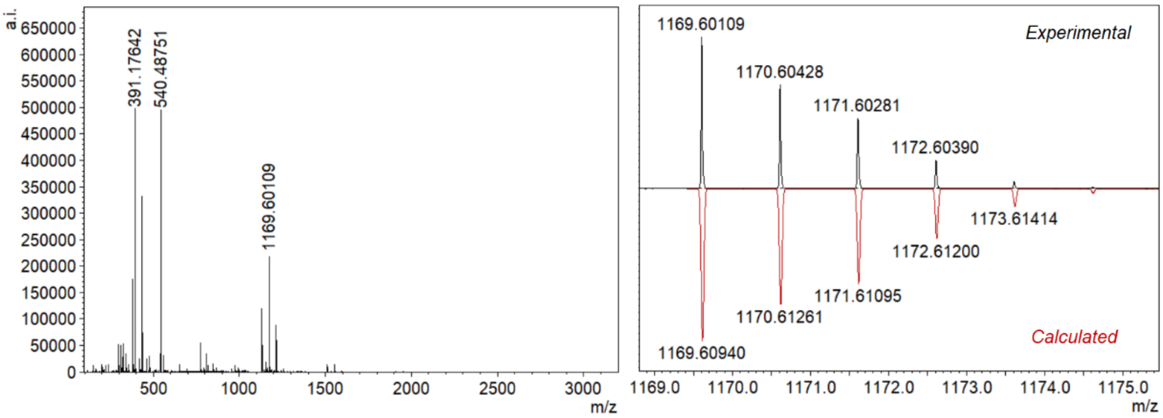

Figure S14: left: high resolution ESI-MS spectrum of **[Cl$^-$⊂*o*-Me$_2$-ur-C4]PPh$_4$$^+$** (solvent: acetonitrile), right: experimental (top) and simulated (bottom) isotopic pattern of product peak.

***o*-Me$_2$-ur-C4**

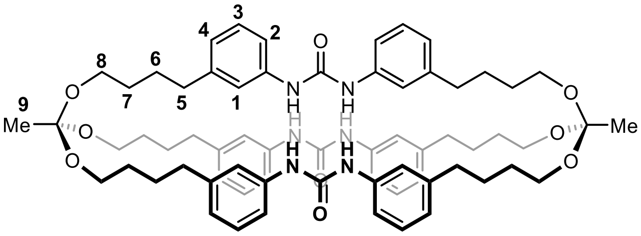

^{1}H NMR (400 MHz, DMSO-*d$_6$*): δ = 8.53 (s_{br}, 6 H, **N*H***), 7.31 (s_{br}, 6 H, **C*H*$_{arom.}$**), 7.24 – 6.99 (m, 12 H, **C*H*$_{arom.}$**), 6.73 (s_{br}, 6 H, **C*H*$_{arom.}$**), 3.39 (s_{br}, 12 H, **8**), 2.50 (s_{br}, 12 H, **5**), 1.64 – 1.38 (m, 24 H, **6/7**), 1.33 (s_{br}, 6 H, **9**) ppm.

^{13}C NMR {^{1}H} (150 MHz, DMSO-*d$_6$*): δ = 152.5, 142.7, 139.6, 128.5, 121.8, 121.8, 118.0, 115.6, 113.5, 79.2, 64.9, 63.6, 61.1, 60.5, 48.6, 35.1, 35.0, 32.1, 28.9, 27.6, 27.4, 20.1, 15.1 ppm.

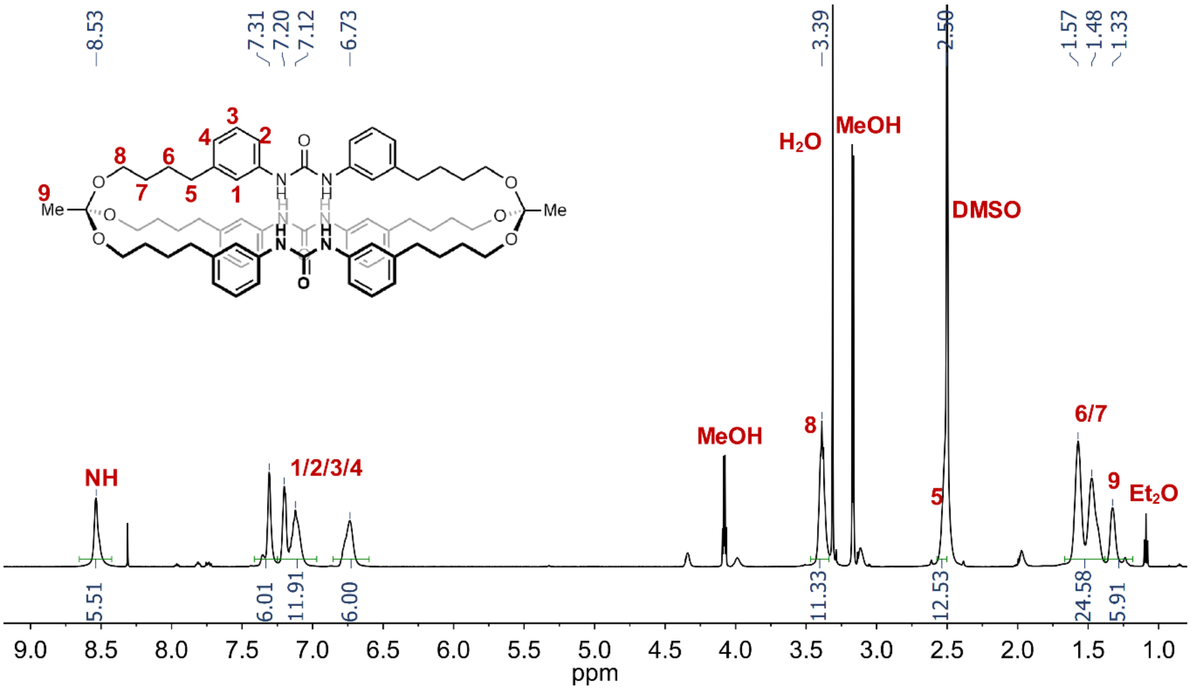

Figure S15: ^{1}H NMR (DMSO-d$_6$, 400 MHz, 293 K) spectrum of ***o*-Me$_2$-ur-C4**.

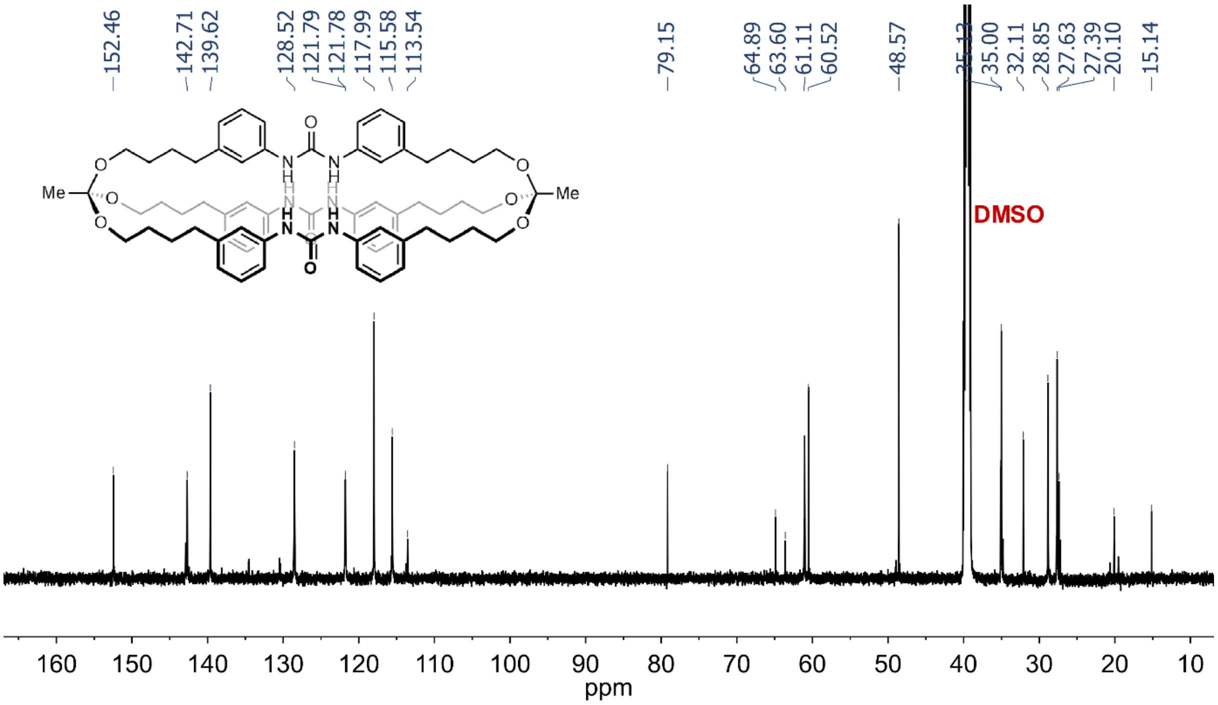

Figure S16: ^{13}C NMR (DMSO-d$_6$, 150 MHz, 293 K) spectrum of ***o*-Me$_2$-ur-C4**.

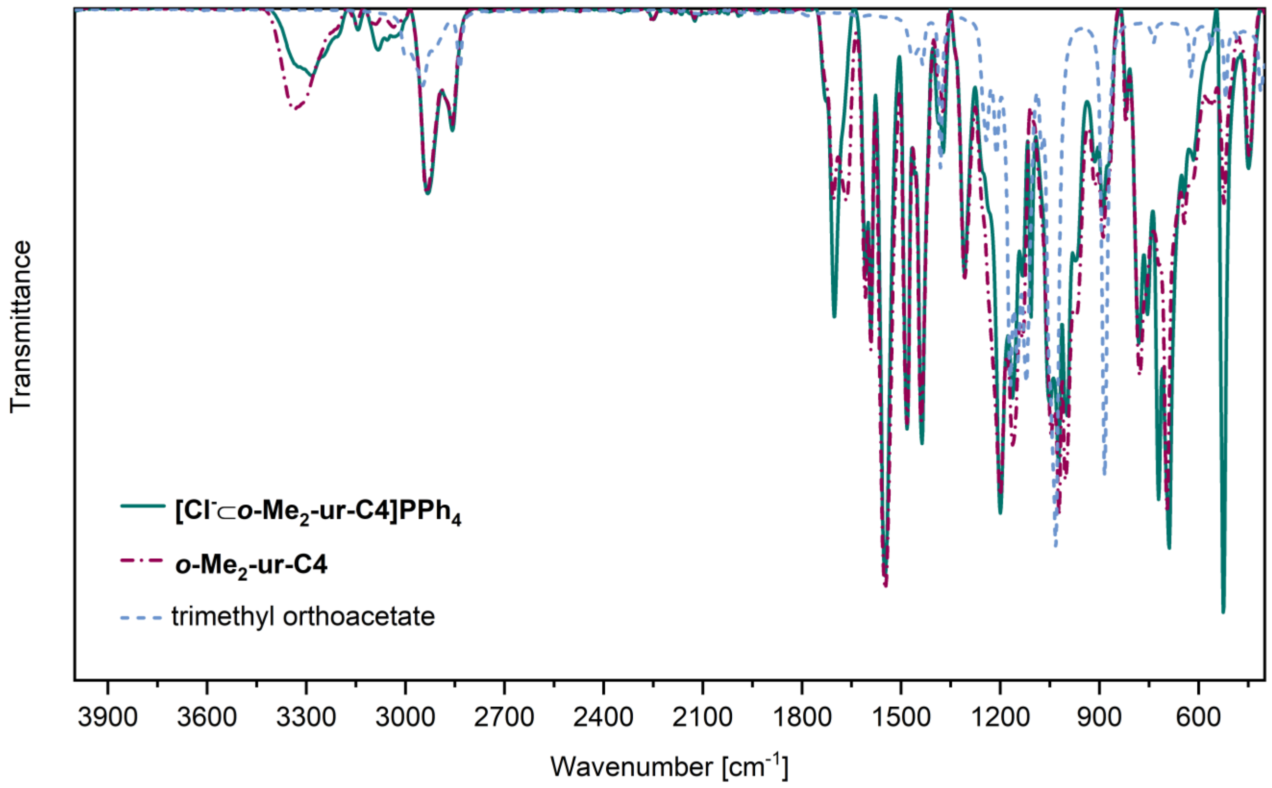

Figure S17: ATR-IR spectra of **[Cl$^-$⊂*o*-Me$_2$-ur-C4]PPh$_4$$^+$**, ***o*-Me$_2$-ur-C4** and trimethyl orthoacetate.

Characterization of ***[Cl$^-$⊂o-H$_2$-ur-C4]PPh$_4^+$*** *and* ***o-H$_2$-ur-C4***

[Cl$^-$⊂*o*-H$_2$-ur-C4]PPh$_4^+$

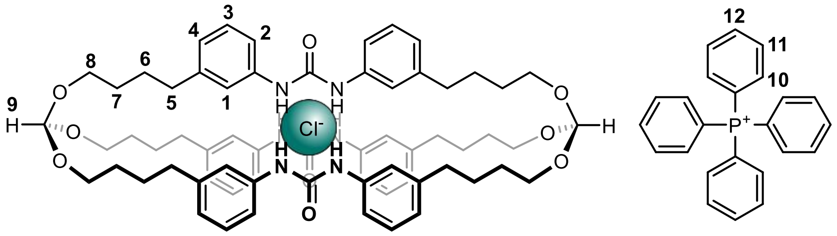

Yield: 9.6 mg (6.6 µmol, 44%)

^{1}H NMR (400 MHz, DMSO-*d$_6$*): δ = 8.73 - 8.50 (m, 6 H, **N*H***), 8.01 – 7.91 (m, 4 H, **12**), 7.87 – 7.68 (m, 16 H, **10/11**), 7.50 - 7.25 (m, 6 H, **C*H$_{arom.}$***), 7.25 – 6.98 (m, 12 H, **C*H$_{arom.}$***), 6.84 – 6.57 (m, 6 H, **C*H$_{arom.}$***), 5.19 – 4.92 (m, 2 H, **9**), 3.59 – 3.23 (m, 12 H, **8**), 2.49 – 2.37 (m, 12 H, **5**), 1.70 – 1.31 (m, 24 H, **6/7**) ppm.

^{13}C NMR {^{1}H} (151 MHz, DMSO-*d$_6$*): δ = 152.4, 142.7, 139.7, 135.4, 134.5, 130.4, 128.5, 121.8, 118.1, 118.0, 117.3, 115.5, 112.5, 63.2, 35.0, 28.6, 27.5 ppm.

ESI-MS: m/z = 1123.57347 [M-PPh$_4$]$^-$ (calculated: m/z = 1123.56753).

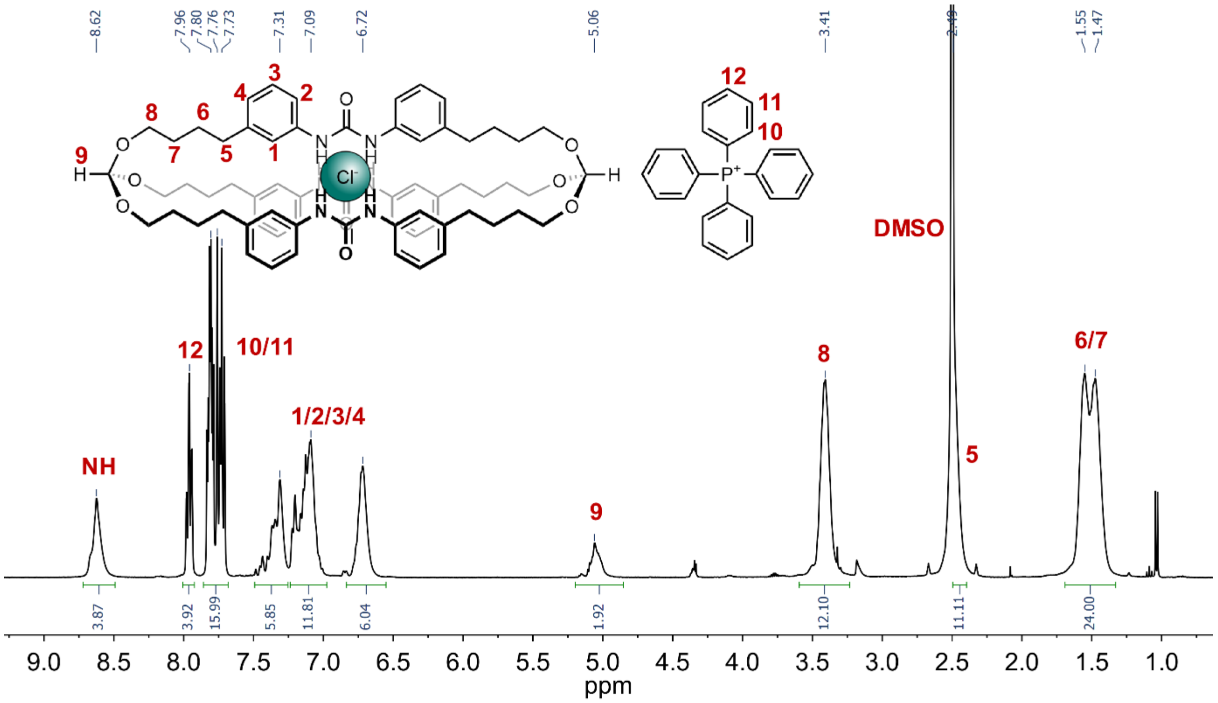

Figure S18: ^{1}H NMR (DMSO-d$_6$, 400 MHz, 293 K) spectrum of **[Cl$^-$⊂*o*-H$_2$-ur-C4]PPh$_4^+$**.

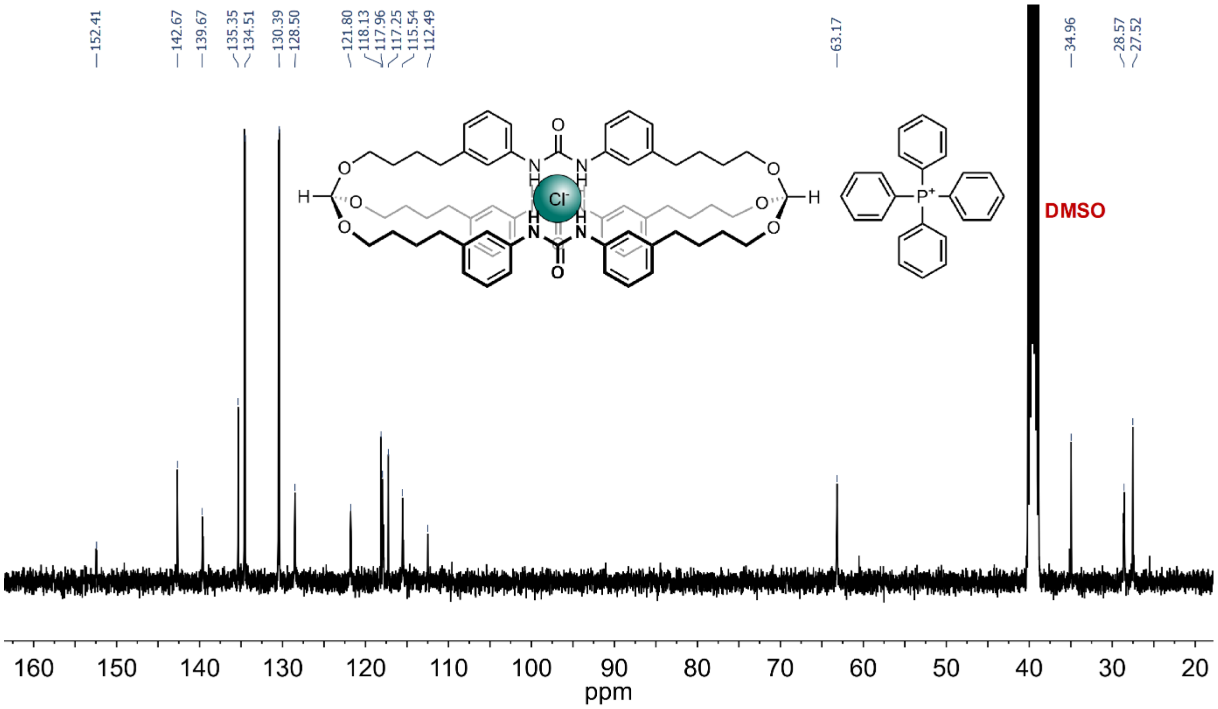

Figure S19: ^{13}C NMR (DMSO-d$_6$, 151 MHz, 293 K) spectrum of **[Cl$^-$⊂*o*-H$_2$-ur-C4]PPh$_4$$^+$**.

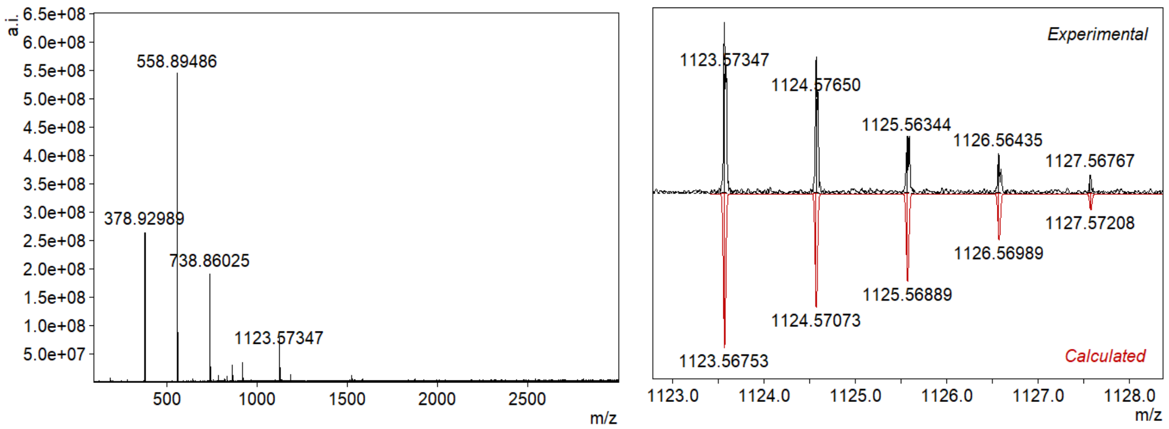

Figure S20: left: high resolution ESI-MS spectrum of **[Cl$^-$⊂*o*-H$_2$-ur-C4]PPh$_4$$^+$** (solvent: acetonitrile), peaks 378.92989, 558.89486, 738.86025 correspond to dimer, trimer and tetramer of PFBT acid, right: experimental (top) and simulated (bottom) isotopic pattern of product peak.

o-H_2-ur-C4

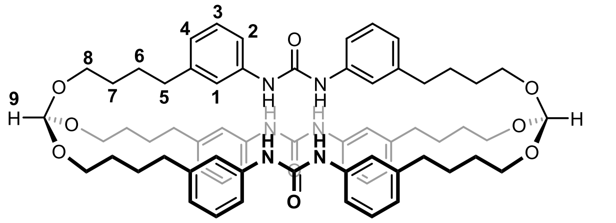

^{1}H NMR (600 MHz, DMSO-*d*$_6$): δ = 8.63 - 8.47 (m, 6 H, N*H*), 7.46 – 7.27 (m, 6 H, C*H*$_{arom.}$), 7.25 – 7.03 (m, 12 H, C*H*$_{arom.}$), 6.85 – 6.63 (m, 6 H, C*H*$_{arom.}$), 5.18 – 4.94 (m, 2 H, **9**), 3.59 – 3.31 (m, 12 H, **8**), 2.65 – 2.51 (m, 12 H, **5**), 1.74 – 1.34 (m, 24 H, **6/7**) ppm.

^{13}C NMR {^{1}H} (151 MHz, DMSO-*d*$_6$): δ = 152.5, 142.7, 139.6, 128.5, 121.8, 118.0, 115.6, 113.0, 63.2, 60.5, 50.7, 48.6, 35.0, 32.1, 28.6, 27.5 ppm.

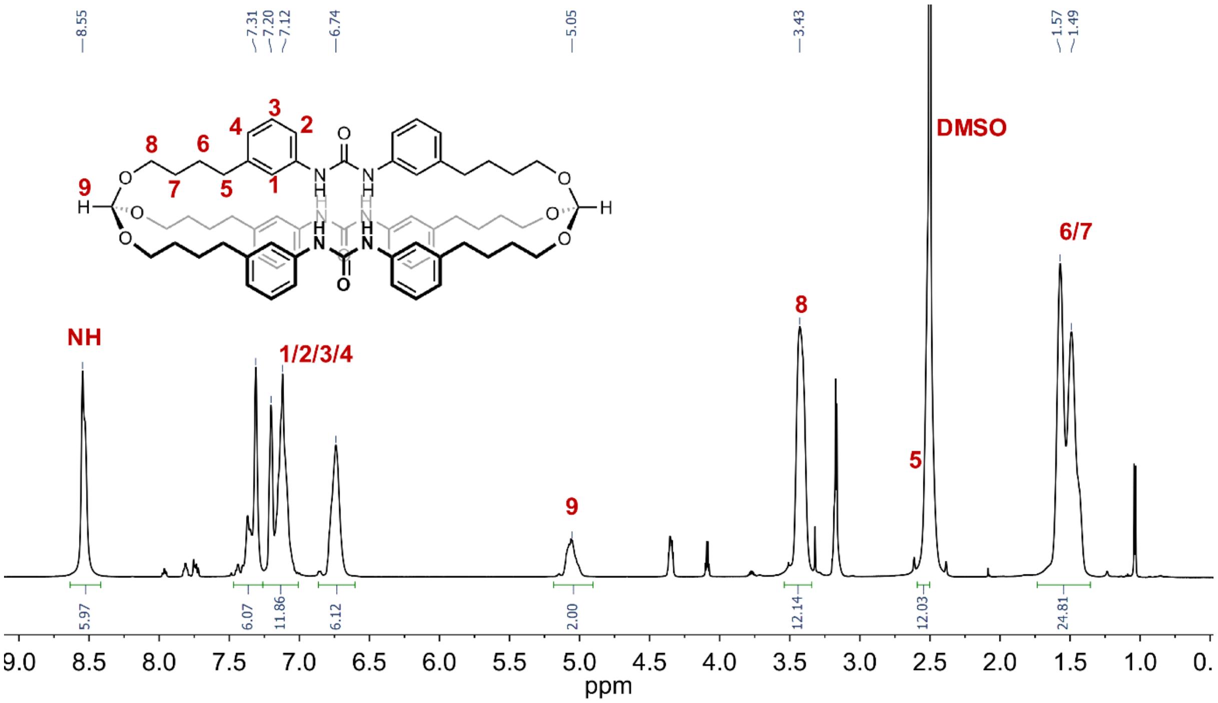

Figure S21: ^{1}H NMR (DMSO-d_6, 600 MHz, 293 K) spectrum of ***o*-H_2-ur-C4**.

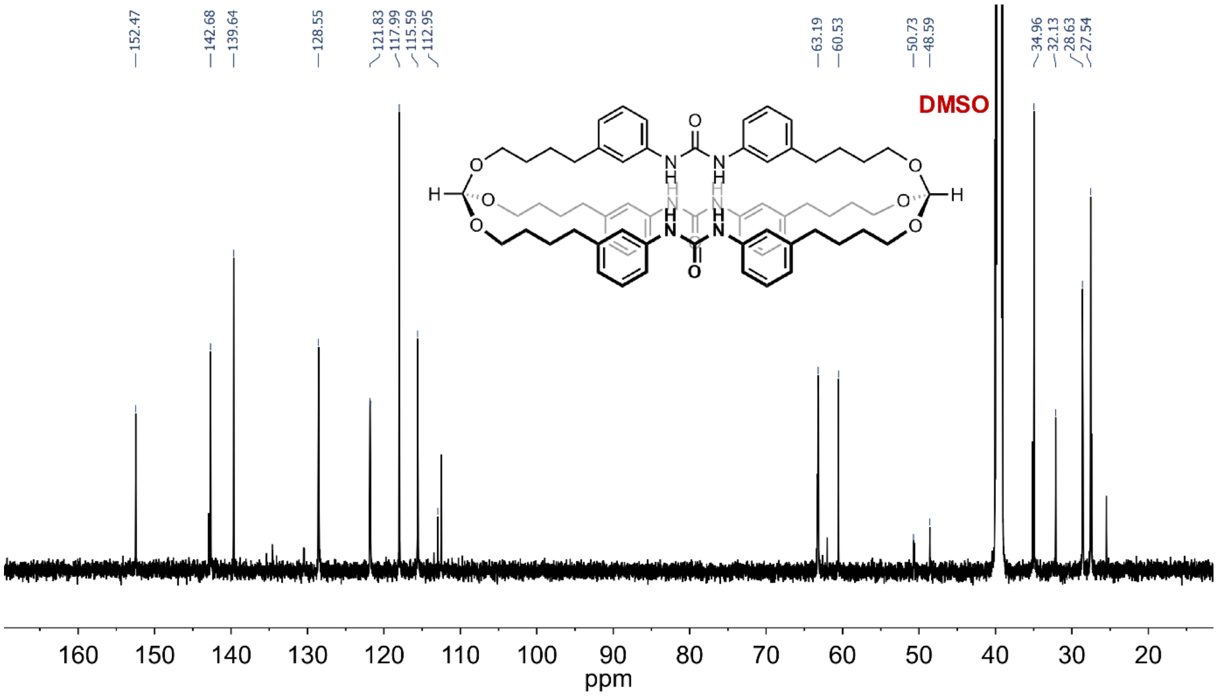

Figure S22: ^{13}C NMR (DMSO-d_6, 151 MHz, 293 K) spectrum of ***o*-H₂-ur-C4**.

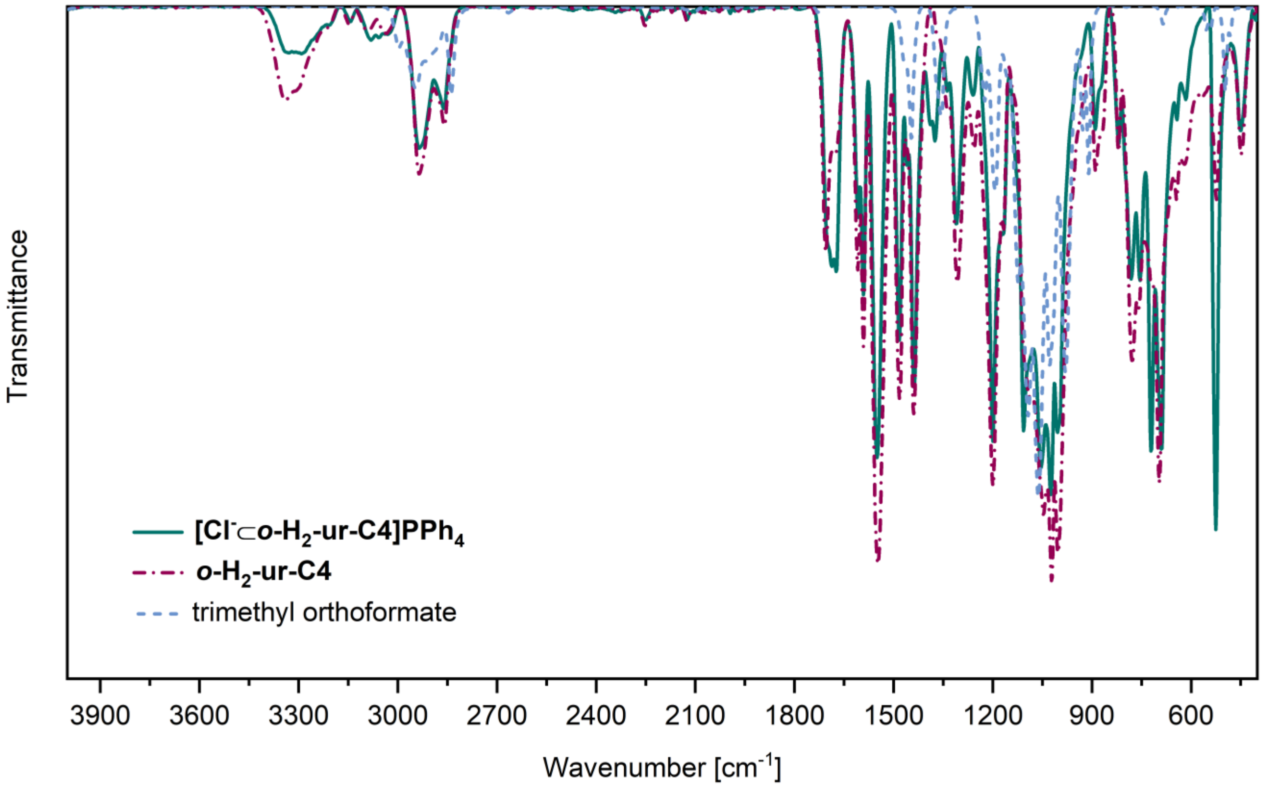

Figure S23: ATR-IR spectra of **[Cl⁻⊂*o*-H₂-ur-C4]PPh₄⁺**, ***o*-H₂-ur-C4** and trimethyl orthoformate.

4. Synthesis and Characterization of Ion-Pair-Templated Cryptates

Synthesis of [Cl^-⊂*o*-Me_2-ur-C2][Cs^+⊂*o*-Me_2-2.2.2]

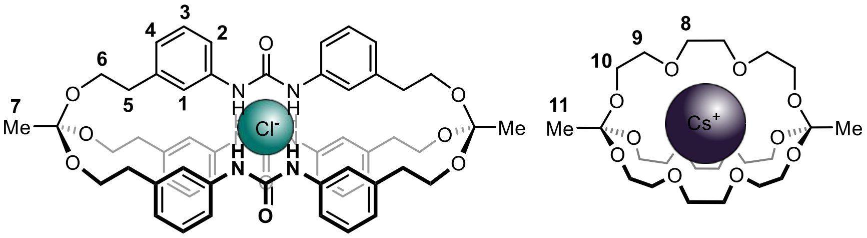

The reaction was performed under nitrogen atmosphere. Diol (**1**) (40.5 mg, 135 µmol, 3 equiv.) and CsCl (7.6 mg, 45 µmol, 1 equiv.) were suspended in 275 µL dry deuterated DMSO and 1.38 mL dry $CDCl_3$. Triethylene glycol (18.4 µL, 20.3 mg, 135 µmol, 3 equiv.), trimethyl orthoacetate (24.0 µL, 22.7 mg, 189 µmol, 4.2 equiv.) and 2,3,4,5,6-pentafluorobenzenethiol (6.0 µL, 9.0 mg, 45 µmol, 1 equiv.) were added. To facilitate the slow removal of emerging methanol during the reaction, 5 Å molecular sieves (around 700 mg) were admixed to the reaction vessel. The reaction progress was monitored by ^{1}H NMR spectroscopy. After disappearance of all signals corresponding to -*OMe* groups (18 h), the reaction mixture was quenched with TEA (125 µL, 90.9 mg, 900 µmol, 10 eq.) and filtered to remove residual molecular sieves. The product was precipitated by the addition of 10 mL dry diethyl ether. After washing with diethyl ether, the pure product was dried under high vacuum.

Yield: 43.0 mg (26.6 µmol, 59%)

^{1}H NMR (400 MHz, DMSO-*d*$_6$): δ = 8.84 – 8.58 (m, 6 H, **N*H***), 7.45 – 7.05 (m, 18 H, **C*H*$_{arom.}$**), 6.91 – 6.66 (m, 6 H, **C*H*$_{arom.}$**), 3.81 – 3.16 (m, 48 H, **6/8/9/10**), 2.81 – 2.57 (m, 12 H, **5**), 1.42 – 1.17 (m, 12 H, **7/11**) ppm.

^{13}C NMR {^{1}H} (101 MHz, DMSO-*d*$_6$): δ = 152.6, 139.7, 139.6, 128.5, 122.4, 118.5, 115.9, 113.9, 69.8, 69.5, 62.5, 61.0, 35.8, 20.1, 20.0 ppm.

ESI-MS: m/z = 983.41276 [M-**[Cs^+⊂*o*-Me_2-2.2.2]**]$^-$ (calculated: m/z = 983.41104), 631.17418 [M-**[Cl^-⊂*o*-Me_2-ur-C2]**]$^+$ (calculated: m/z = 631.17307).

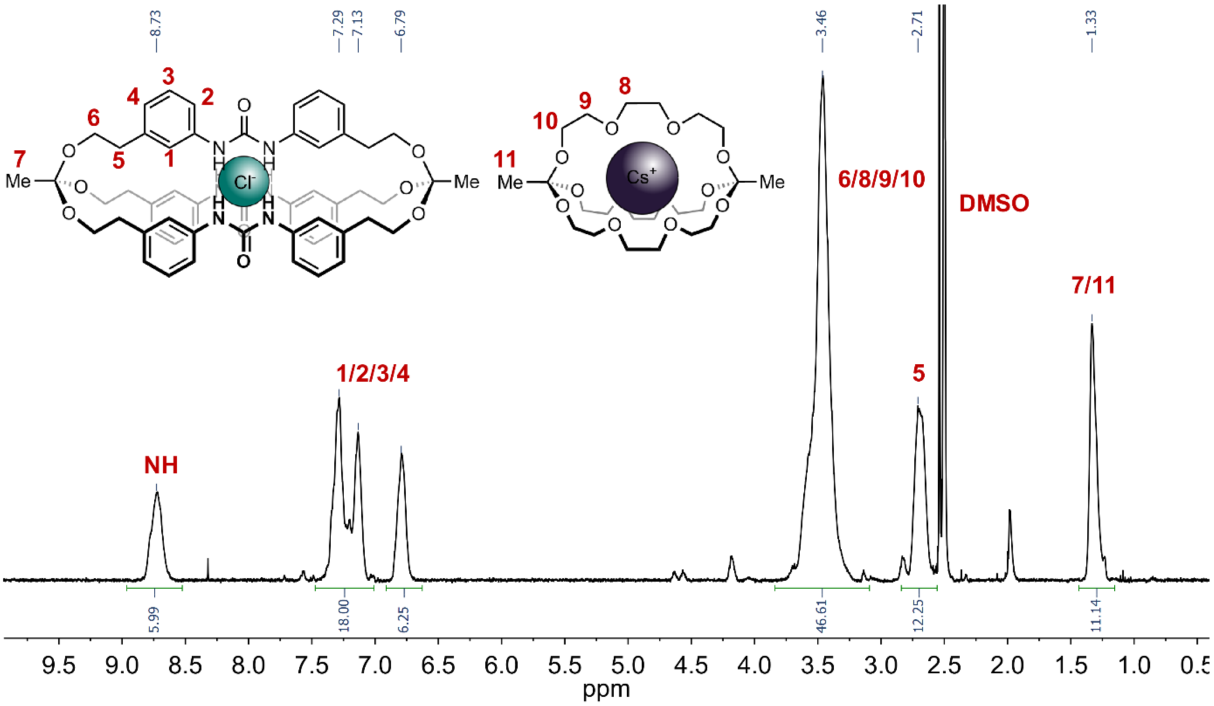

Figure S24: ^{1}H NMR (DMSO-d$_6$, 400 MHz, 293 K) spectrum of **[Cl$^-$⊂*o*-Me$_2$-ur-C2][Cs$^+$⊂*o*-Me$_2$-2.2.2]**.

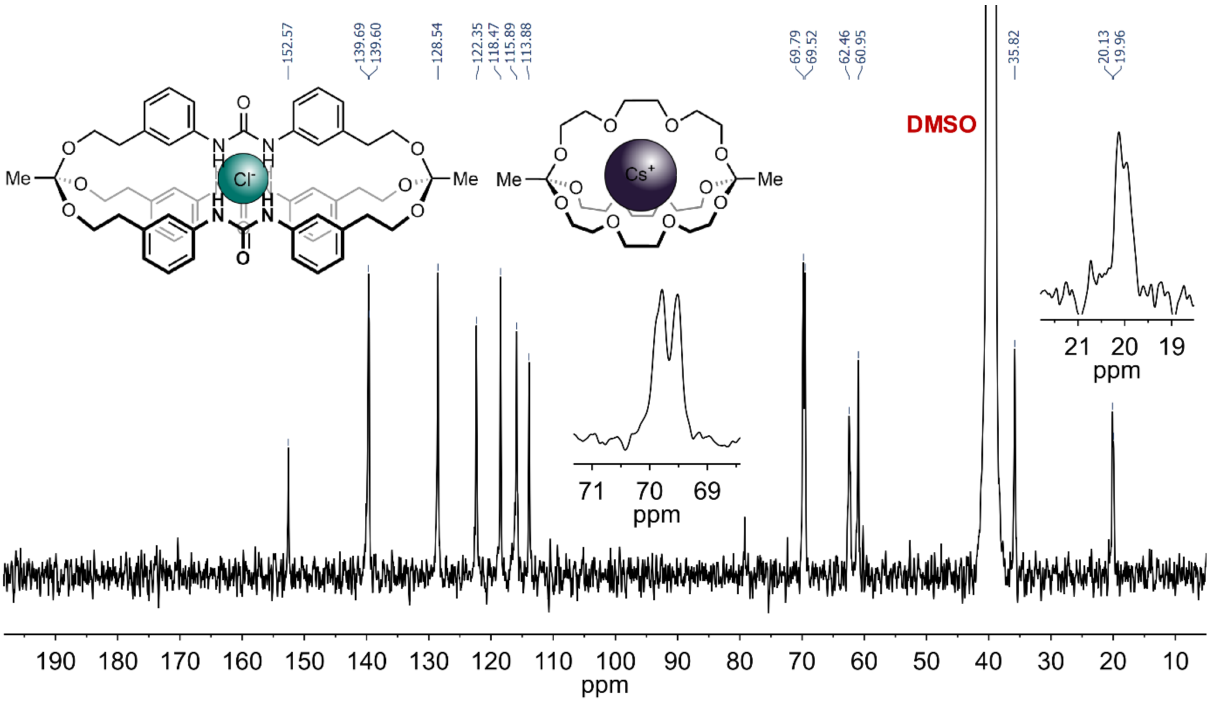

Figure S25: ^{13}C NMR (DMSO-d$_6$, 101 MHz, 293 K) spectrum of **[Cl$^-$⊂*o*-Me$_2$-ur-C2][Cs$^+$⊂*o*-Me$_2$-2.2.2]**.

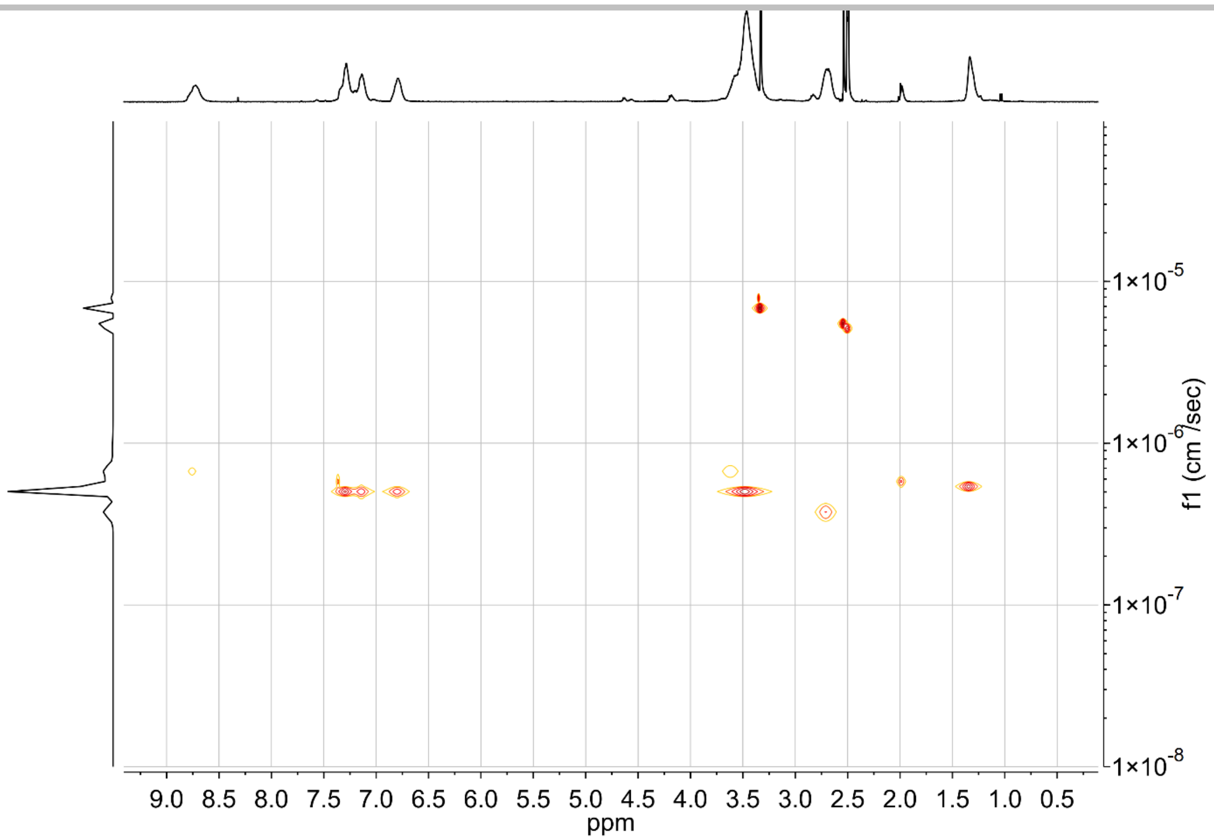

Figure S26: DOSY-NMR (DMSO-d$_6$, 400 MHz, 293 K) spectrum of **[Cl$^-$⊂*o*-Me$_2$-ur-C2][Cs$^+$⊂*o*-Me$_2$-2.2.2]**.

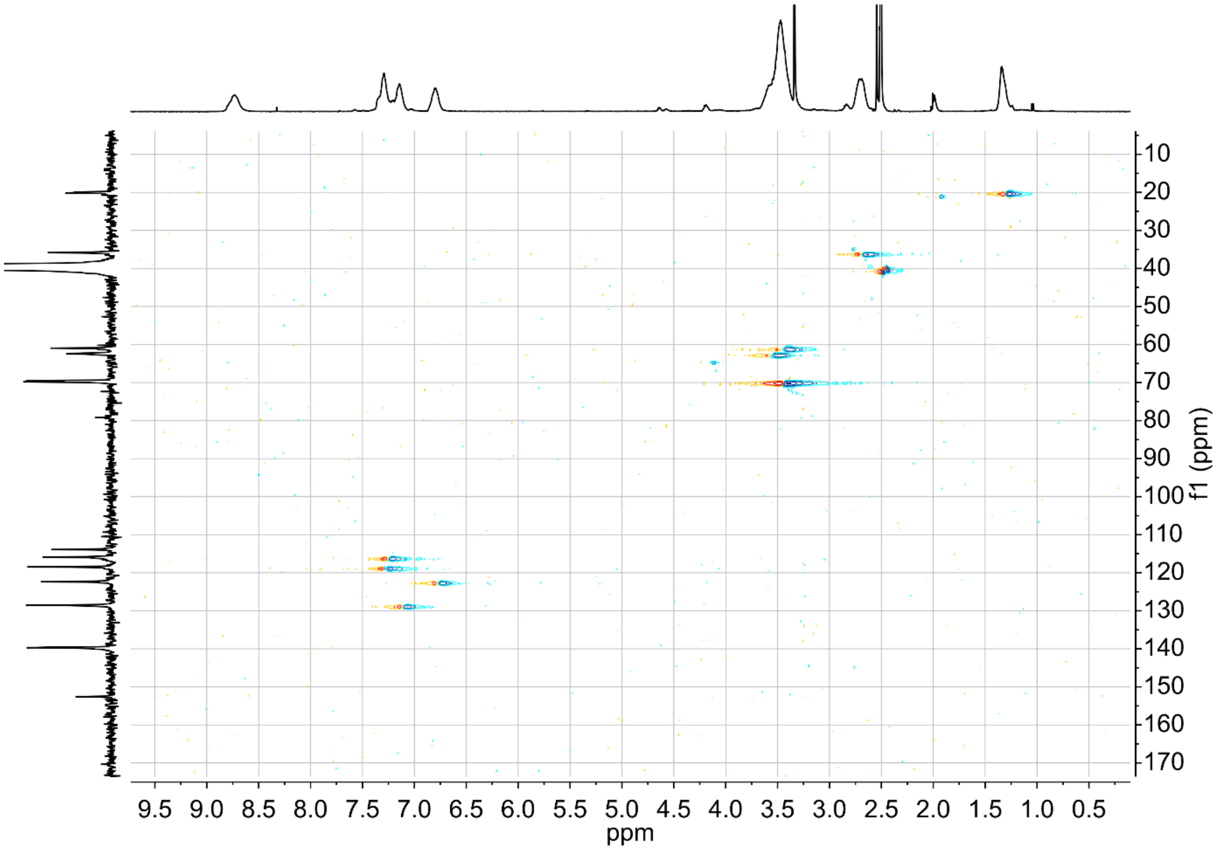

Figure S27: HSQC NMR (DMSO-d$_6$, 400 MHz, 293 K) spectrum of **[Cl$^-$⊂*o*-Me$_2$-ur-C2][Cs$^+$⊂*o*-Me$_2$-2.2.2]**.

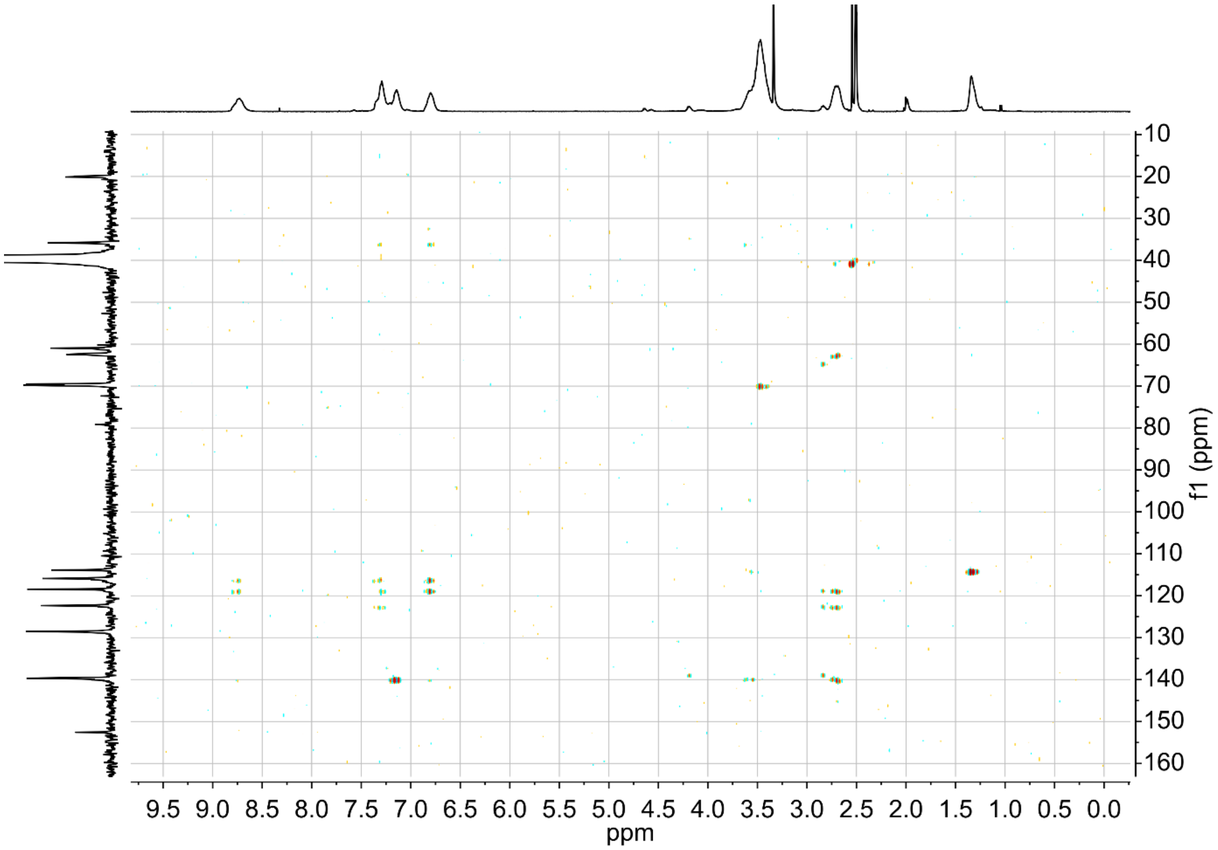

Figure S28: HMBC NMR (DMSO-d$_6$, 400 MHz, 293 K) spectrum of **[Cl$^-$⊂*o*-Me$_2$-ur-C2][Cs$^+$⊂*o*-Me$_2$-2.2.2]**.

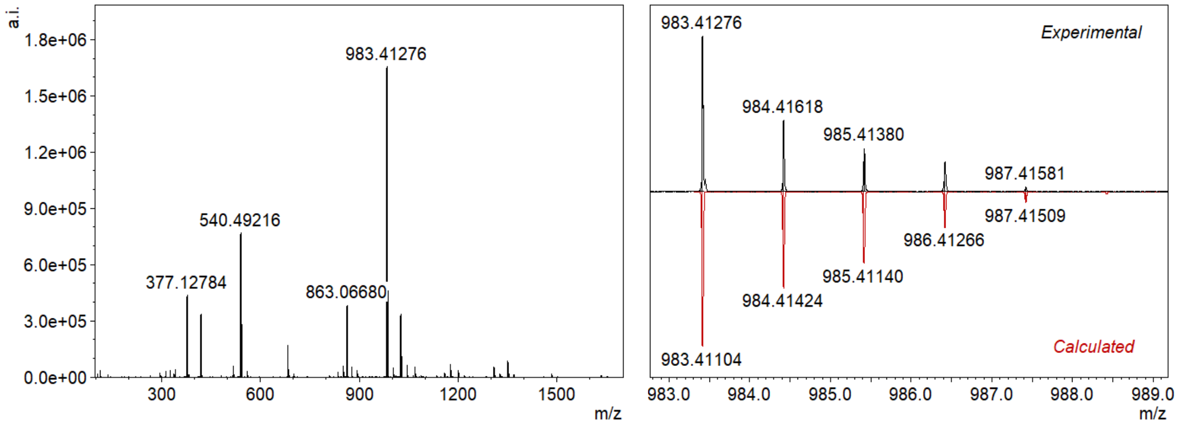

Figure S29: left: high resolution ESI-MS spectrum in negative mode of **[Cl$^-$⊂*o*-Me$_2$-ur-C2][Cs$^+$⊂*o*-Me$_2$-2.2.2]** (solvent: acetonitrile), right: experimental (top) and simulated (bottom) isotopic pattern of product peak.

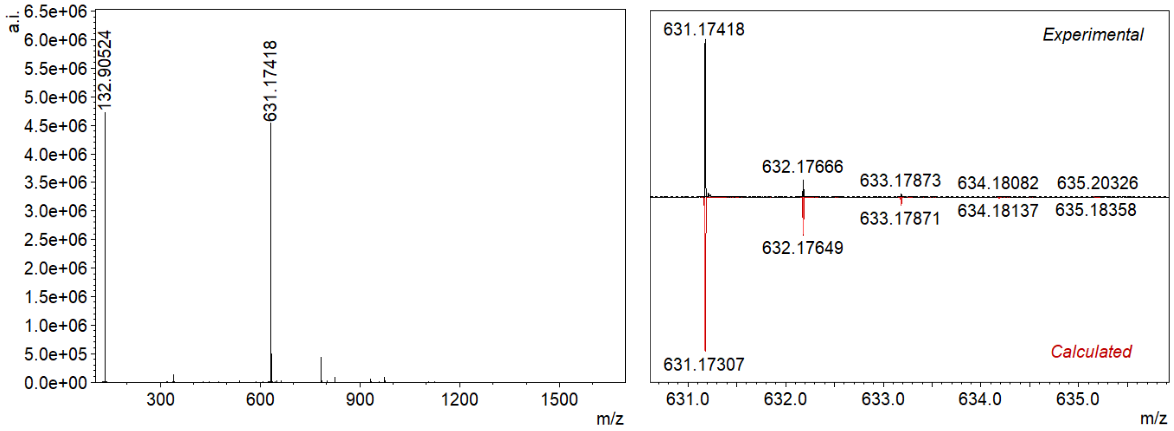

Figure S30: left: high resolution ESI-MS spectrum in positive mode of **[Cl$^-$⊂*o*-Me$_2$-ur-C2][Cs$^+$⊂*o*-Me$_2$-2.2.2]** (solvent: acetonitrile), right: experimental (top) and simulated (bottom) isotopic pattern of product peak.

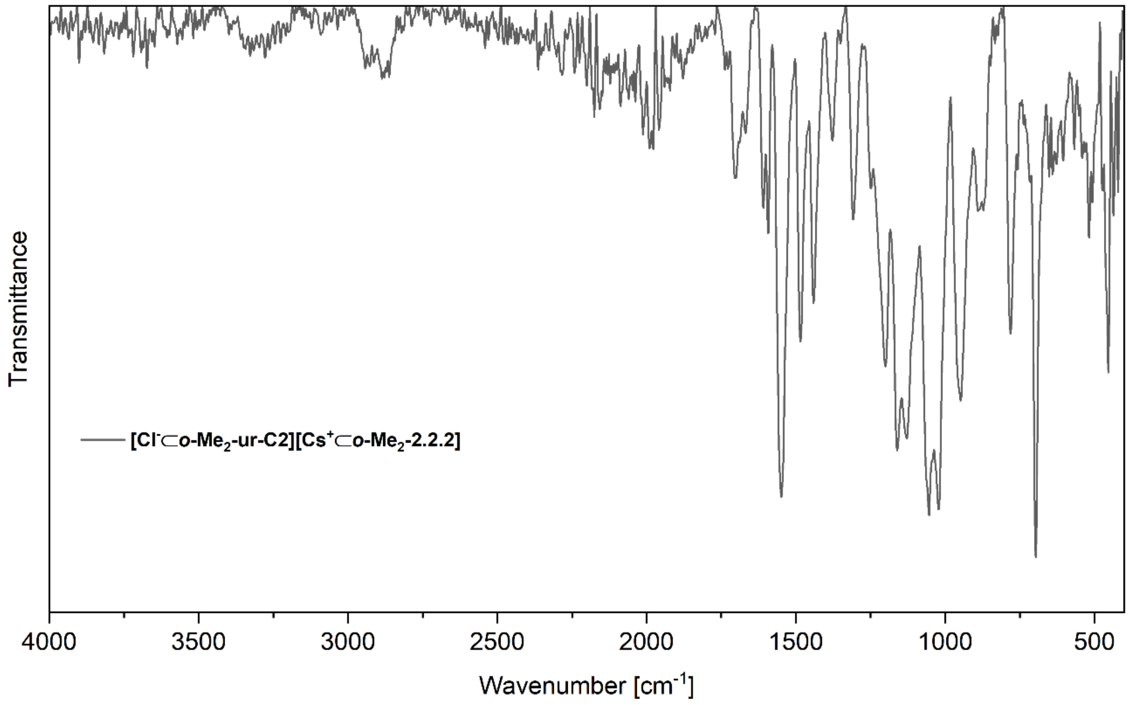

Figure S31: ATR-IR spectrum of **[Cl$^-$⊂*o*-Me$_2$-ur-C2][Cs$^+$⊂*o*-Me$_2$-2.2.2]**.

Synthesis of *o*-Me$_2$-ur-C2 *o*-Me$_2$-2.2.2 (<u>CsCl removal</u>)

To a solution of about 6 mg of **[Cl$^-$⊂*o*-Me$_2$-ur-C2][Cs$^+$⊂*o*-Me$_2$-2.2.2]** in 600 µL DMSO, 500 µL D_2O was added. The resulting precipitate was isolated by centrifugation. 1 mL D_2O was added and the suspension was ultrasonicated and subsequently centrifuged. This step was repeated 2 times. The product was dried in vacuum. A ^{133}Cs NMR spectrum was recorded and some residual Cs^+ could be detected. Further washing with D_2O did not lead to complete removal of the Cs^+ signal.

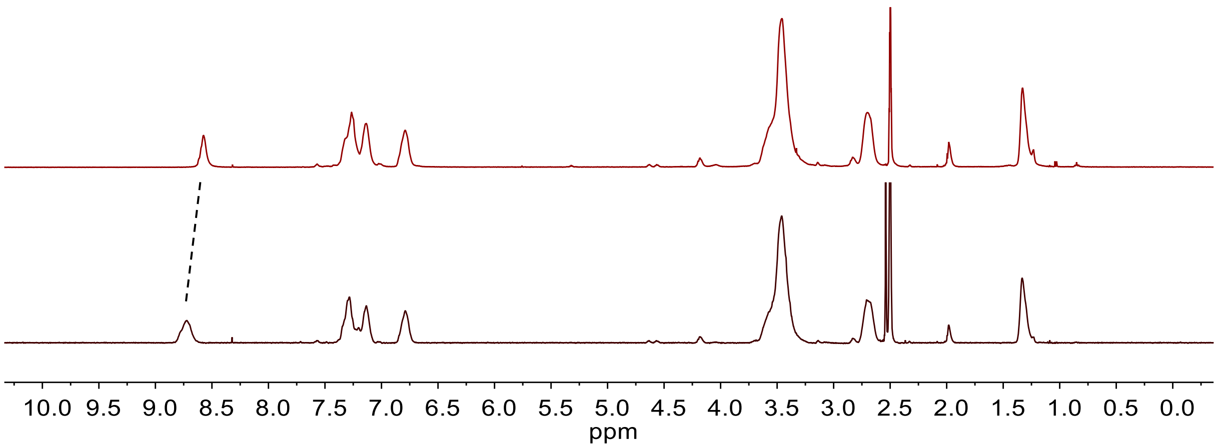

Figure S32: Upper ^{1}H NMR (DMSO-d$_6$) spectrum: precipitate after washing with D_2O, lower ^{1}H NMR (DMSO-d$_6$) spectrum: before washing with D_2O, dotted line indicating the shift of the signal corresponding to NH proton.

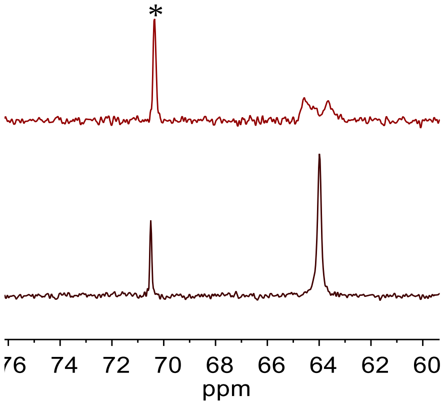

Figure S33: Upper ^{133}Cs NMR (DMSO-d$_6$) spectrum: precipitate after washing with D_2O, lower ^{133}Cs NMR (DMSO-d$_6$) spectrum: before washing with D_2O, * pristine CsCl in DMSO-d$_6$ as external standard (glass insert).

5. Miscellaneous Experiments

5.1 Evidence for template effect I: Attempting synthesis of ***o*-Me2-ur-C3** without PPh_4Cl

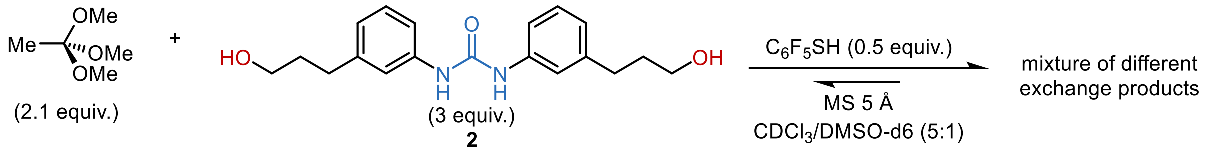

General procedure (A) was followed**.** Without addition of TPPCl. Molecular sieves were renewed after 2.5 days.

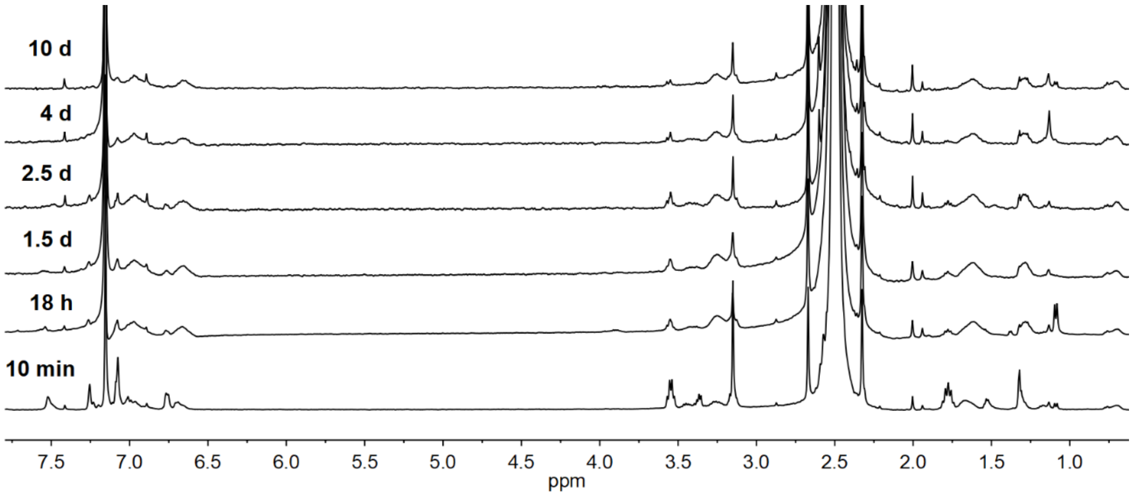

Figure S34: ^1H NMR ($CDCl_3$, 400 MHz, 293 K) spectra of reaction control after 1 min to 10 days showing incomplete consumption of trimethyl orthoacetate (indicated by -OMe signals at 3.2 ppm).

5.2 Evidence for template effect II: Unsuccessful synthesis of [Cl$^-$⊂*o*-Me$_2$-ur-C2][Cs$^+$⊂*o*-Me$_2$-2.2.2] in pure DMSO

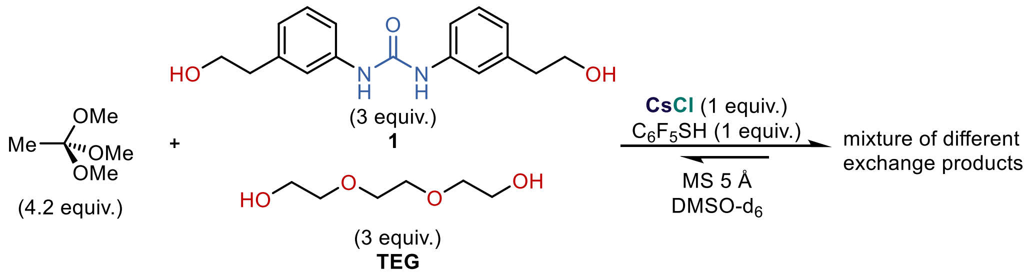

General procedure (A) was followed**.** Pure DMSO-d_6 was used as solvent.

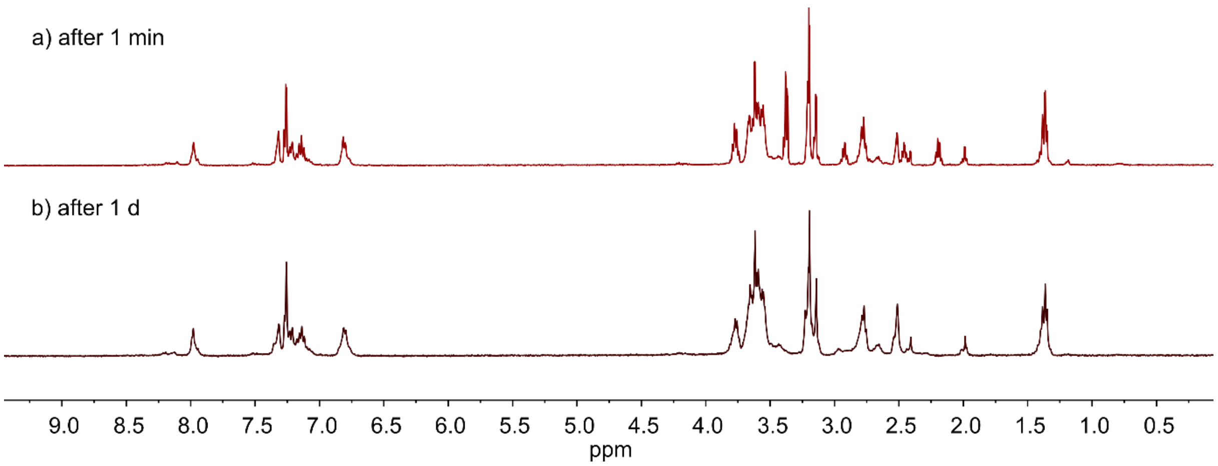

Figure S35: a)+b) ^{1}H NMR (CDCl$_3$, 400 MHz, 293 K) spectra of reaction control after 1 min and 1 day showing decrease of -OMe signals at 3.3 ppm.

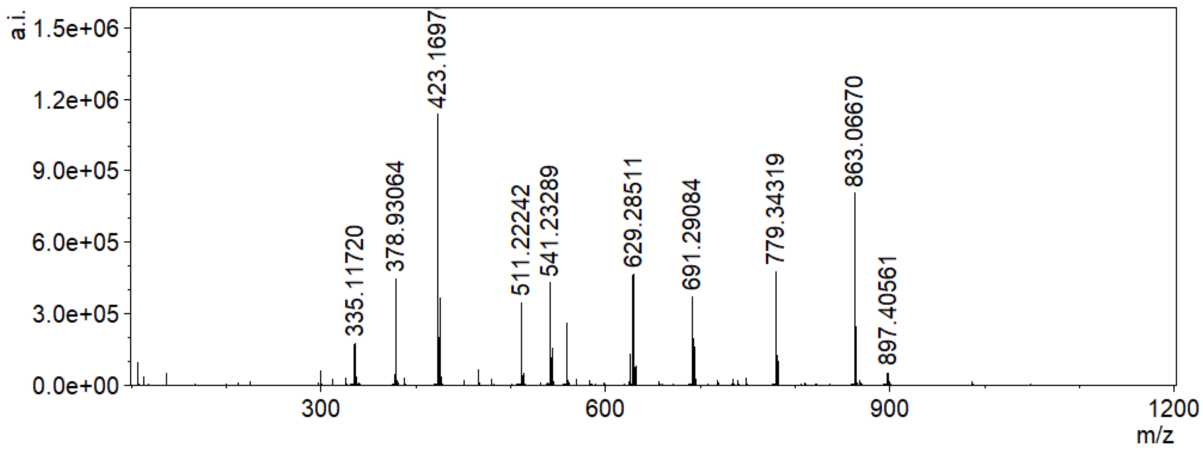

Figure S36: high resolution ESI-MS spectrum in negative mode for reaction control after 1 day. No product peak observable. ([M-**[Cs$^+$⊂o-Me2-2.2.2]**]$^-$ calculated: m/z = 983.41104).

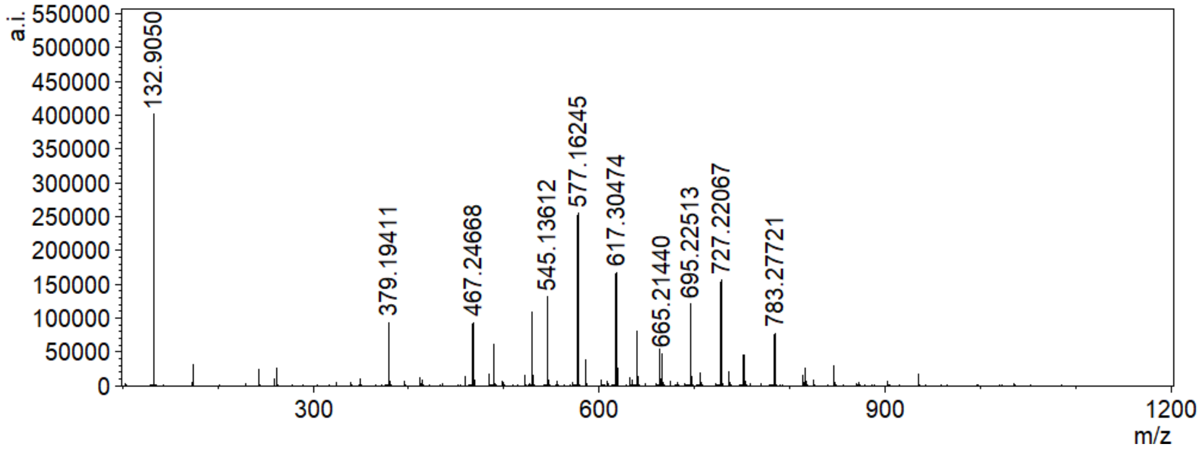

Figure S37: high resolution ESI-MS spectrum in positive mode for reaction control after 1 day. No product peak observable. ([M-**[Cl$^-$⊂*o*-Me$_2$-ur-C2]**]$^+$ (calculated: m/z = 631.17307).

5.3 Comparison of ATR-IR spectra of **[Cl⁻⊂*o*-Me2-ur-C2][Cs⁺⊂*o*-Me2-2.2.2]**, **[Cl⁻⊂*o*-Me2-ur-C2]PPh3⁺** and **[Cs⁺⊂*o*-Me2-2.2.2]BArF⁻**

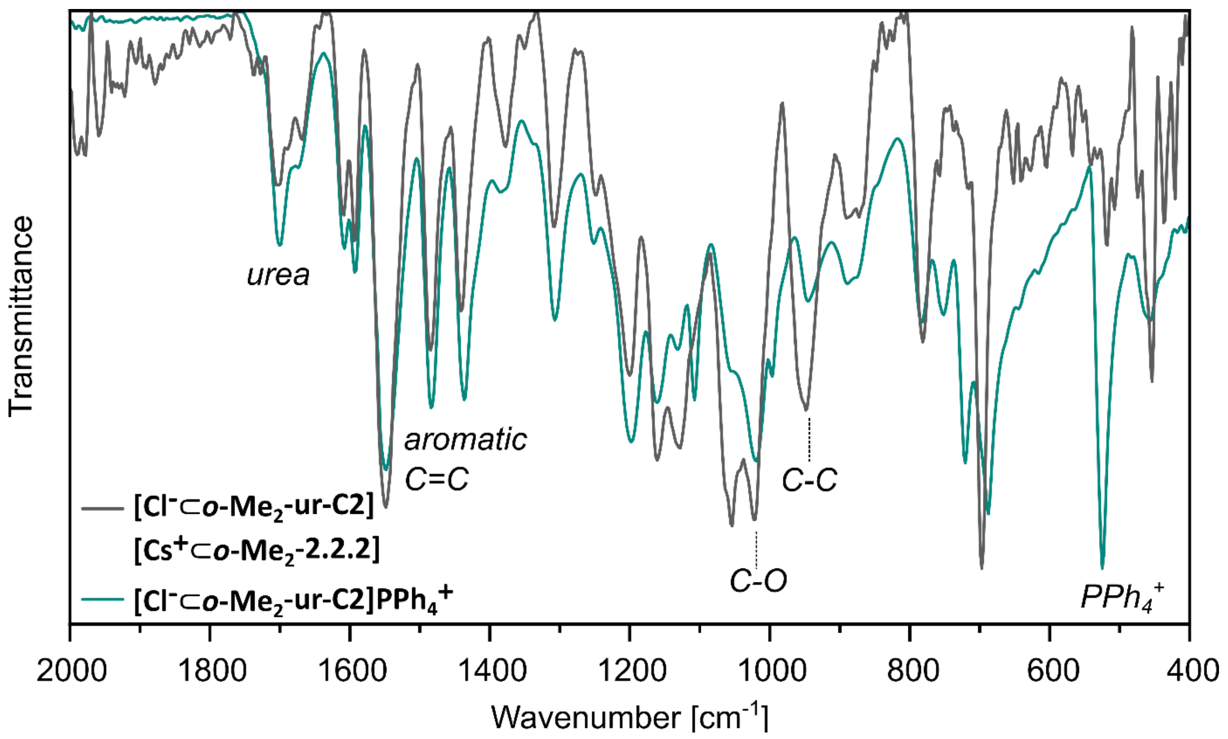

Figure S38: Comparison of ATR-IR spectra of **[Cl⁻⊂*o*-Me2-ur-C2][Cs⁺⊂*o*-Me2-2.2.2]** and **[Cl⁻⊂*o*-Me2-ur-C2]PPh3⁺**.

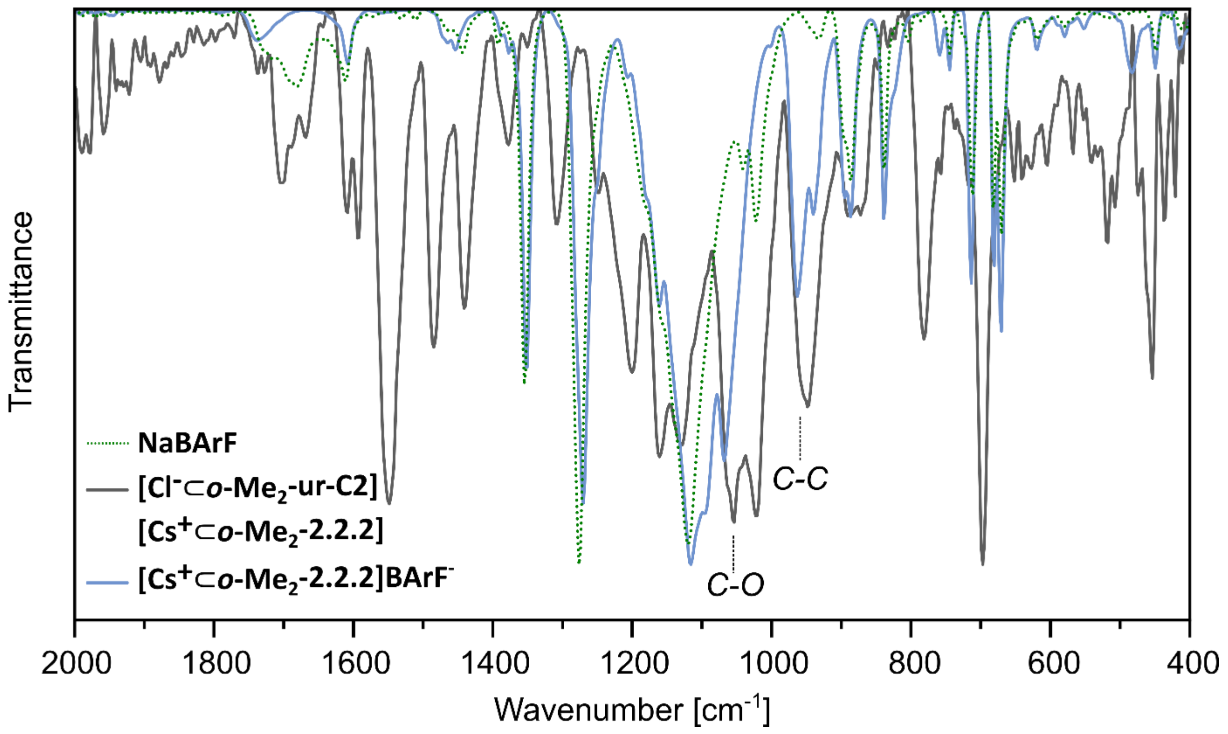

Figure S39: Comparison of ATR-IR spectra of **[Cl⁻⊂*o*-Me2-ur-C2][Cs⁺⊂*o*-Me2-2.2.2]**, **[Cs⁺⊂*o*-Me2-2.2.2]BArF⁻** and **NaBArF**.

5.4 ^{1}H NMR and ^{133}Cs NMR studies of binding of CsCl to **[Cl$^-$⊂*o*-Me$_2$-ur-C2][Cs$^+$⊂*o*-Me$_2$-2.2.2]**

A stock solution of **[Cl$^-$⊂*o*-Me$_2$-ur-C2][Cs$^+$⊂*o*-Me$_2$-2.2.2]** in DMSO-d$_6$ was prepared and the precise concentration of the solution was determined by ^{1}H NMR spectroscopy using 1,3,5-trimethoxybenzene as internal standard. A stock solution of CsCl in Methanol was prepared and exact amounts of the solution for each data point were transferred into HPLC vials. The solvent was removed under reduced pressure and 650 µL of a cryptand stock solution were added. To ensure complete dissolving of CsCl, the vials were heated to 80 °C and subsequently vortexed. 600 µL of prepared solutions were transferred to NMR tubes and ^{1}H NMR and ^{133}Cs NMR spectra were recorded.

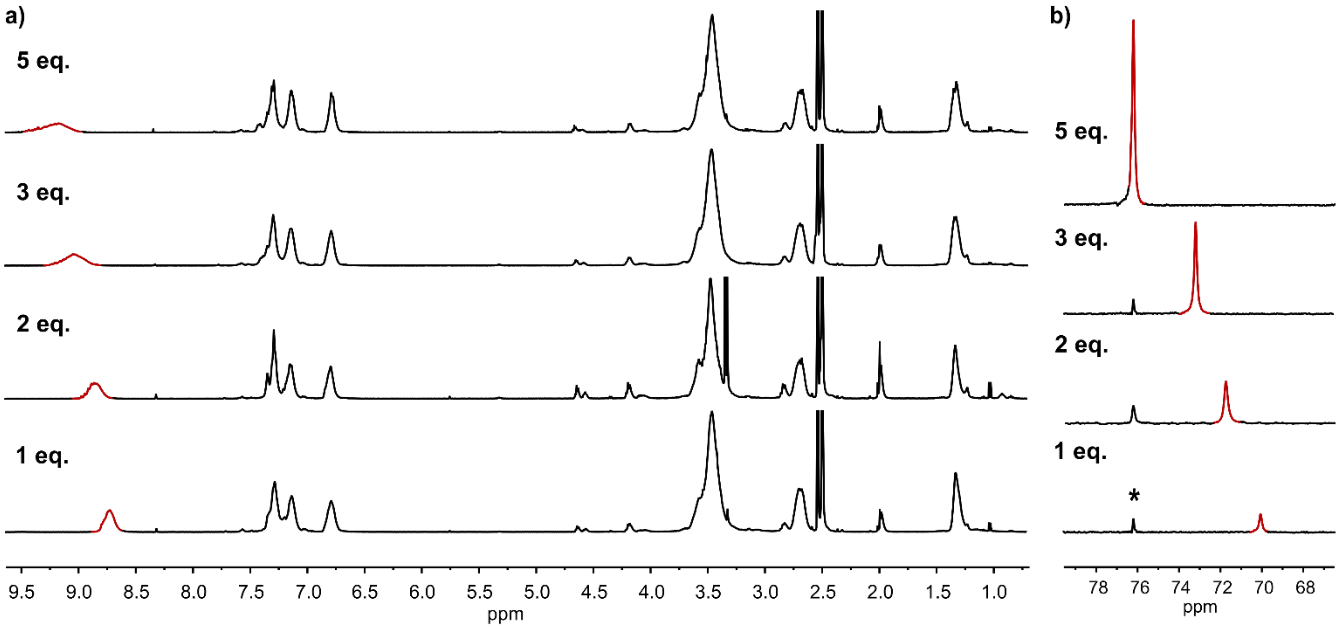

Figure S40: a) ^{1}H NMR (400 MHz, 298 K, DMSO-d$_6$) and b) ^{133}Cs-NMR (52 MHz, 298 K, DMSO-d$_6$), (* CsCl in DMSO-d$_6$ as external standard (glass insert)) stack plots for a titration of **[Cl$^-$⊂*o*-Me$_2$-ur-C2][Cs$^+$⊂*o*-Me$_2$-2.2.2]** (8.0 mM) with CsCl.

5.5 Competition Experiment: Synthesis of **[Cl$^-$⊂*o*-Me$_2$-ur-C2][Cs$^+$⊂*o*-Me$_2$-2.2.2]** with under-stoichiometric amounts of trimethyl orthoacetate

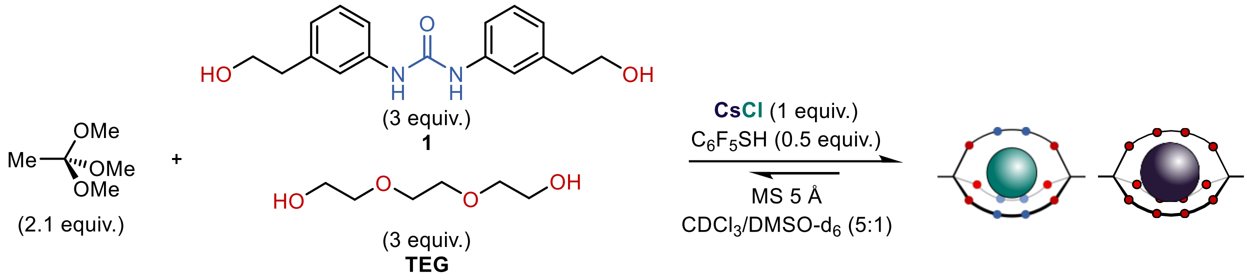

Synthesis procedure for **[Cl$^-$⊂*o*-Me$_2$-ur-C2][Cs$^+$⊂*o*-Me$_2$-2.2.2]** was followed, but only 2.1 equivalents of trimethyl orthoacetate were used. The reaction progress was monitored by ^{1}H NMR spectroscopy. After one day, disappearance of all signals corresponding to -*OMe* groups indicated completion of reaction. After workup (precipitation using diethyl ether), ^{1}H NMR, ^{13}C NMR and ESI-MS spectra were recorded. The precipitate is not the clean product, due to co-precipitation of both residual diols (these have to be present in this type of a competition experiment). Due to overlapping (broad) signals in the ^{1}H NMR spectrum (Figure S41), the ratio between both cryptates was determined using quantitative ^{13}C NMR spectroscopy using suppressed NOE coupling. The integration of such ^{13}C NMR spectra can be used for a rough estimation of compound ratios. A mixture of both cryptates in the ratio of 2:1 **[Cl$^-$⊂*o*-Me$_2$-ur-C2]/[Cs$^+$⊂*o*-Me$_2$-2.2.2]** was observed (in the isolated reference compound, as expected, a 1:1 ratio was observed).

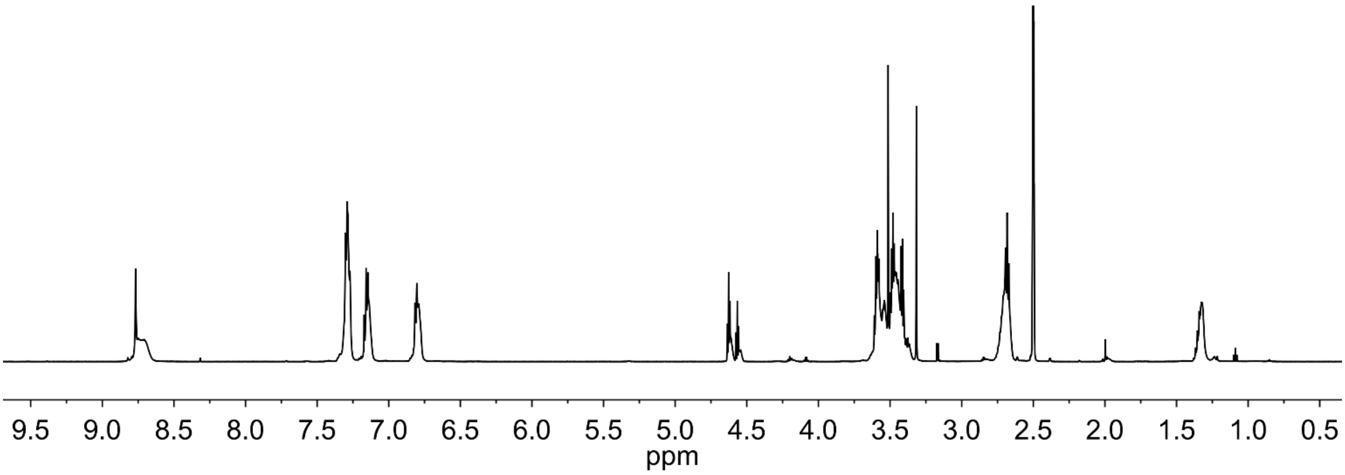

Figure S41: ^{1}H-NMR (DMSO-d_6, 600 MHz, 293 K) spectrum of precipitate. Spectrum shows a mixture of **[Cl$^-$⊂*o*-Me$_2$-ur-C2][Cs$^+$⊂*o*-Me$_2$-2.2.2]**, diol **1** and **TEG**.

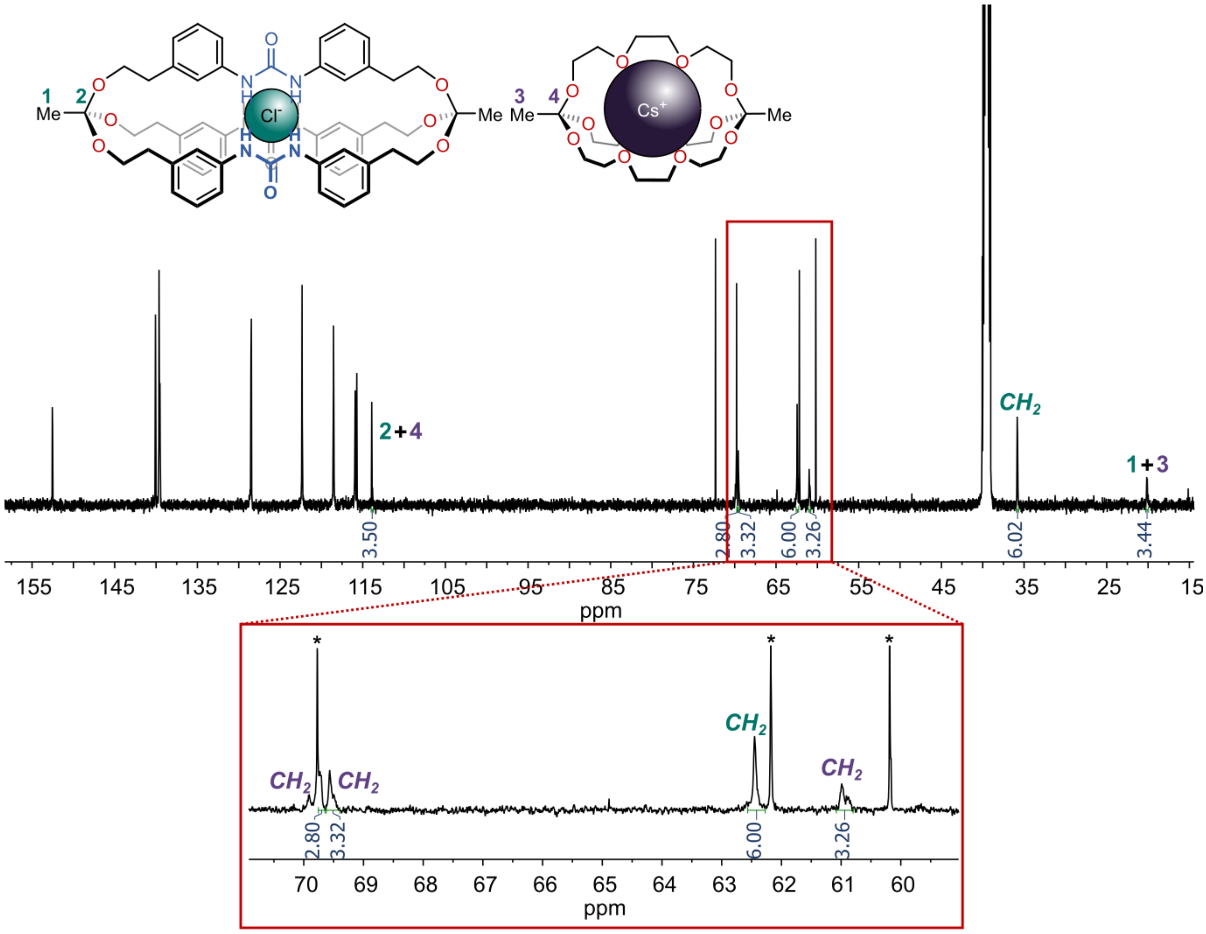

Figure S42 ^{13}C NMR (DMSO-d$_6$, 150 MHz, 293 K) spectra (with suppressed NOE coupling) of precipitation after reaction with 2.1 equiv. trimethyl orthoacetate. Peak assignment according to HSQC (Figure S27) and HMBC (Figure S28) spectra. Ratio of cryptates determined by integration of signals corresponding to CH_2 groups of **[Cl$^-$⊂*o*-Me$_2$-ur-C2]** (62.5 ppm) and **[Cs$^+$⊂*o*-Me$_2$-2.2.2]** (61.0 ppm): 6:3.3. *Sharp signals correspond to residual diols. Reference spectrum of pure **[Cl$^-$⊂*o*-Me$_2$-ur-C2][Cs$^+$⊂*o*-Me$_2$-2.2.2]** shows 1:1 ratio.

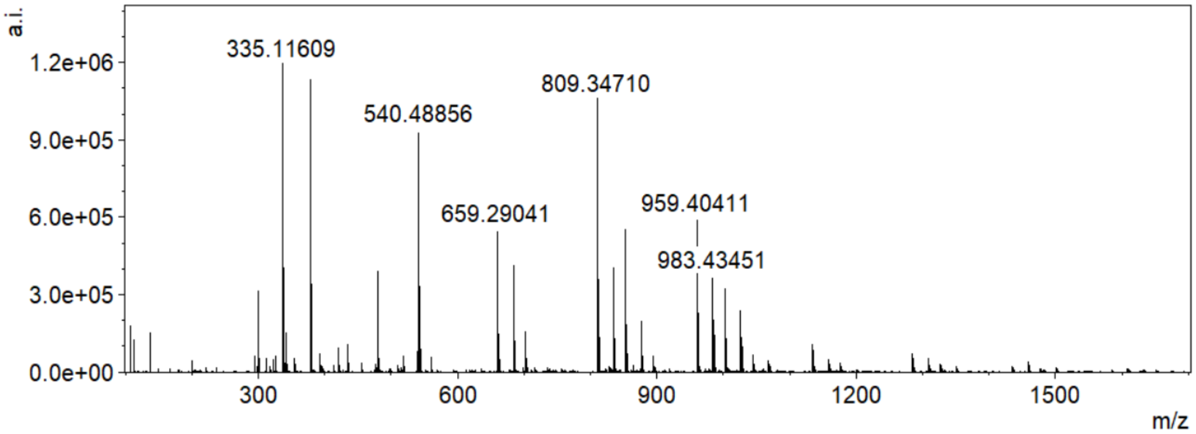

Figure S43: high resolution ESI-MS spectrum in <u>negative mode</u> of the precipitate. Product peak ([M-**[Cs$^+$⊂*o*-Me$_2$-2.2.2]**]$^-$) 983.43451 m/z calculated: m/z = 983.41104).

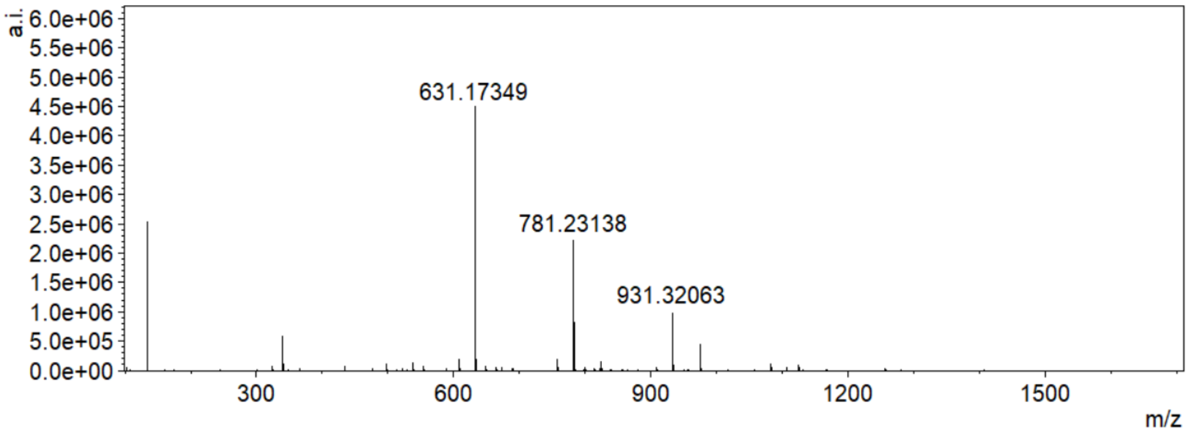

Figure S44: high resolution ESI-MS spectrum in positive mode of the precipitate. Product peak ([M-**[Cl$^-$⊂*o*-Me$_2$-ur-C2]**]$^+$) 631.17349 m/z (calculated: m/z = 631.17307).

5.6 Using CsCl as a template: Synthesis of **[Cl⁻⊂*o*-Me$_2$-ur-C2]Cs$^+$**

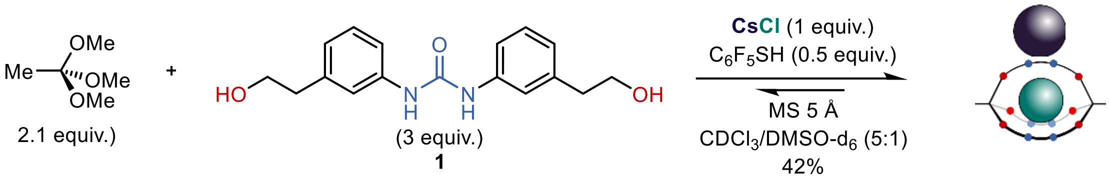

General procedure (A) was followed**.** TPPCl was substituted by CsCl. The reaction progress was monitored by ^{1}H NMR spectroscopy. After one day, disappearance of all signals corresponding to -*OMe* groups indicated completion of reaction. After workup (precipitation using diethyl ether), the clean product **[Cl⁻⊂*o*-Me$_2$-ur-C2]Cs$^+$** was isolated in 42% yields.

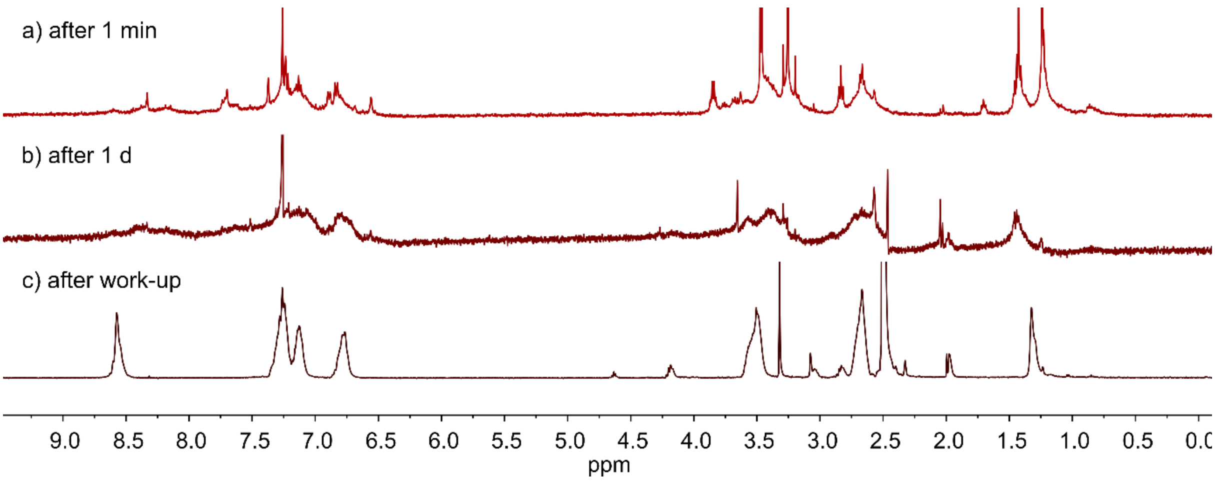

Figure S45: a)+b) ^{1}H-NMR ($CDCl_3$, 400 MHz, 293 K) spectra of reaction control after 1 min and 1 day showing decrease of -OMe signals at 3.3 ppm, c) ^{1}H-NMR (DMSO-d$_6$, 400 MHz, 293 K) spectrum of **[Cl⁻⊂*o*-Me$_2$-ur-C2]Cs$^+$**.

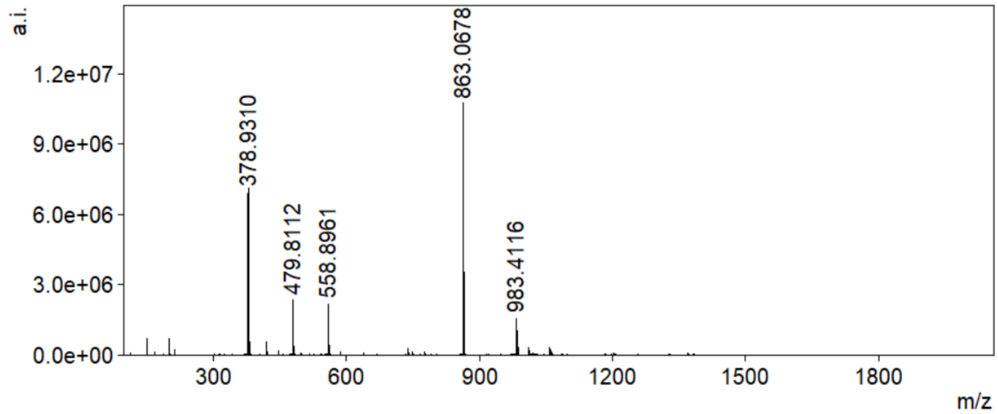

Figure S46: high resolution ESI-MS spectrum in negative mode of **[Cl⁻⊂*o*-Me$_2$-ur-C2]Cs$^+$**. Product peak [M-Cs]$^+$: 983.4116 m/z (calculated: m/z = 983.41104). Residual peaks originate from solvent or mass spectrometer.

5.7 Using CsCl as a template: Unsuccessful synthesis of **[Cs⁺⊂*o*-Me$_2$-2.2.2]Cl$^-$**

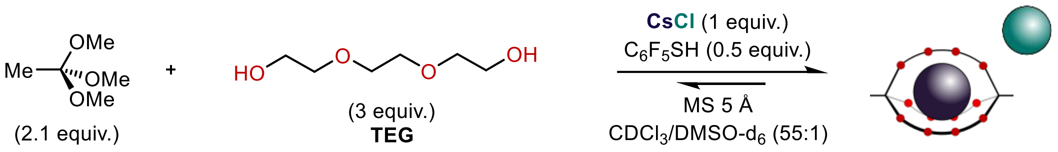

General procedure (A) was followed. Triethylene glycol was used as diol and TPPCl was substituted by CsCl. To support cation binding to the glycol chains, the amount of chloroform was increased (55:1 $CDCl_3$/DMSO-d_6). Due to the low solubility of CsCl in chloroform, DMSO could not completely be omitted. Reaction control by ^{1}H NMR spectroscopy and HRMS provides evidence of cryptate formation, but the isolation of the cryptate was unsuccessful. Hence, only traces of cryptate were formed during the reaction.

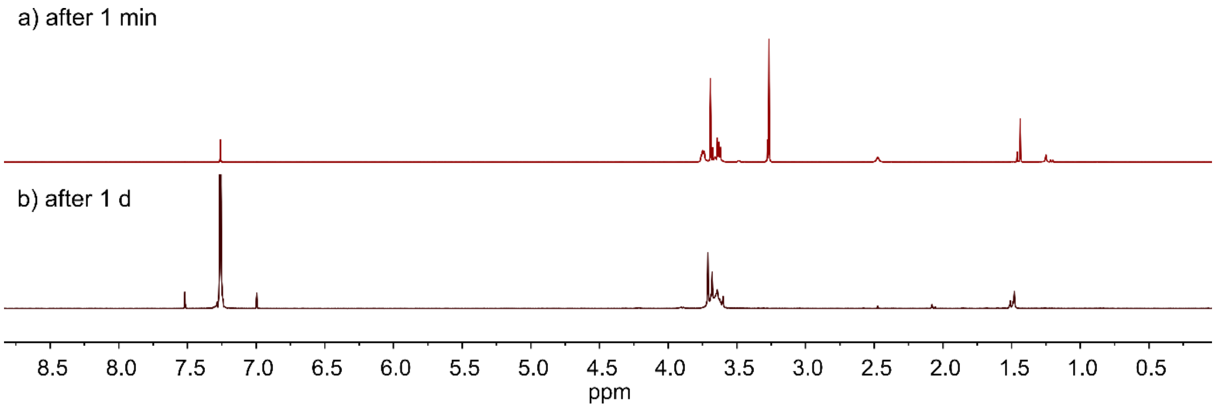

Figure S47: a)+b) ^{1}H-NMR ($CDCl_3$, 400 MHz, 293 K) spectra of reaction control after 1 min and 1 day showing decrease of -OMe signals at 3.3 ppm.

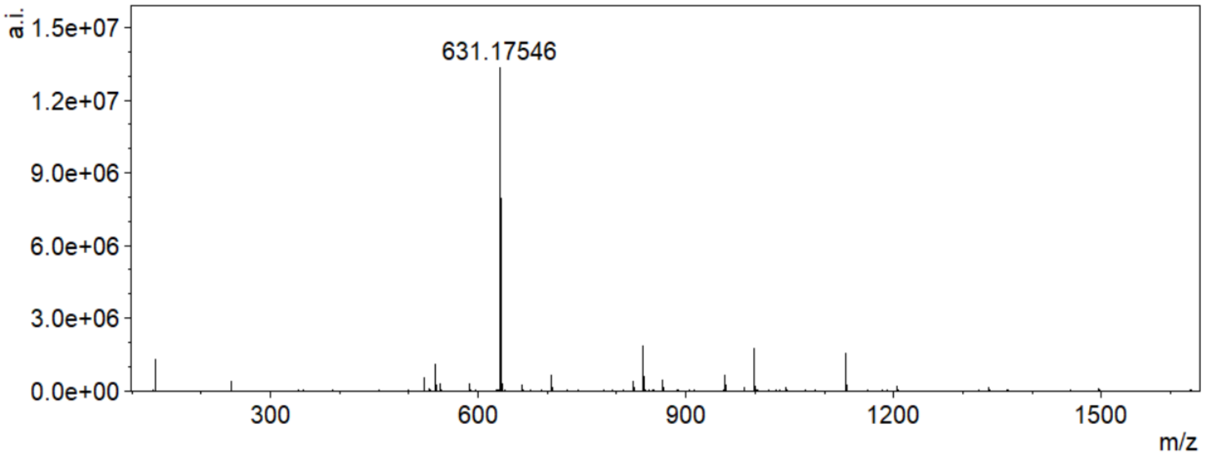

Figure S48: high resolution ESI-MS spectrum in positive mode for reaction control after 1 day. Product peak [M-Cl]$^+$: 631.17546 m/z (calculated: m/z = 631.17307).

6. Computational Chemistry

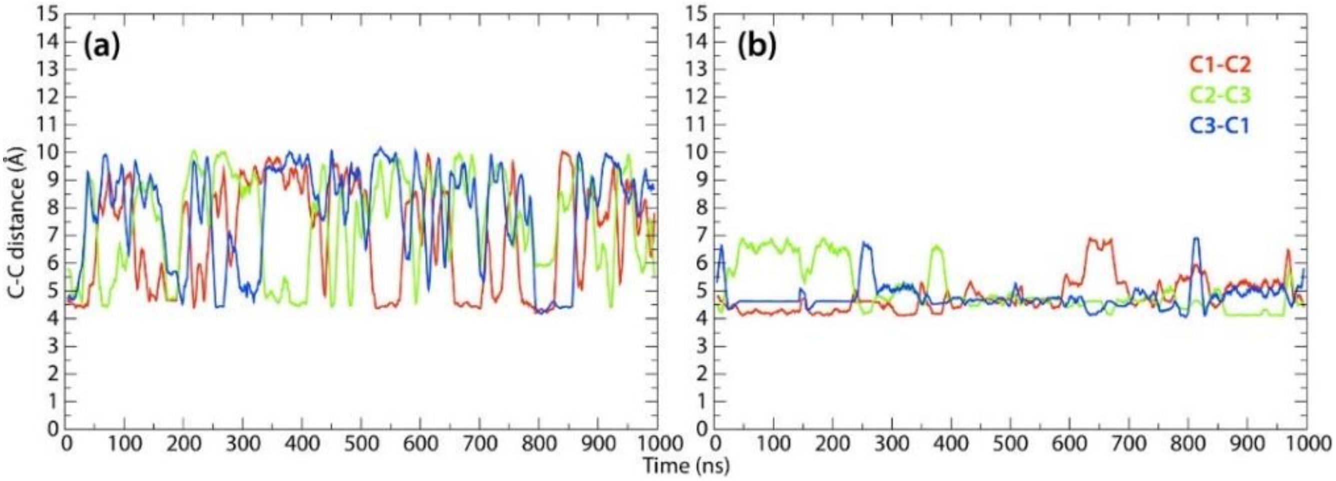

Figure S49: Distance between each carbon atom from urea carbonyl groups of cryptand in pure (a) DMSO and (b) chloroform.

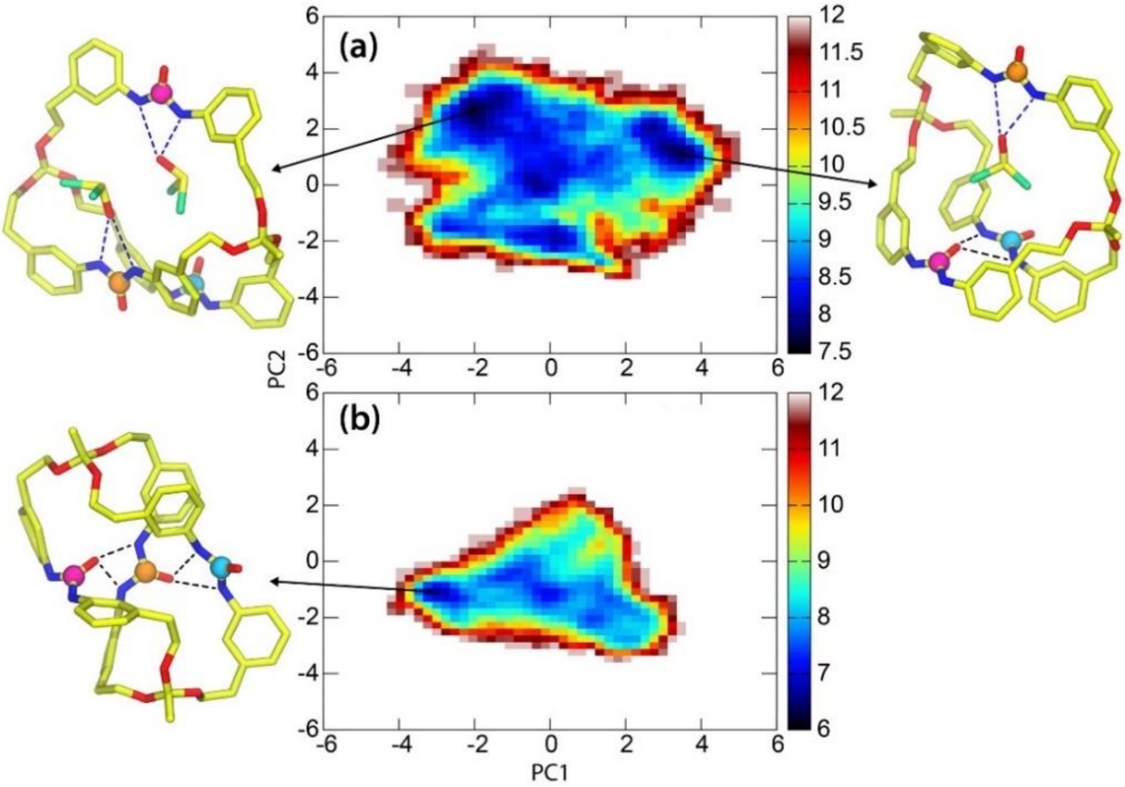

Figure S50: Free energy landscape (relative energy scale in kBT) along first two principal components constructed using ensemble of 20000 snapshots of cryptand in pure (a) DMSO and (b) chloroform at 300 K. Representative structures including hydrogen bonds (shown as dashed lines).

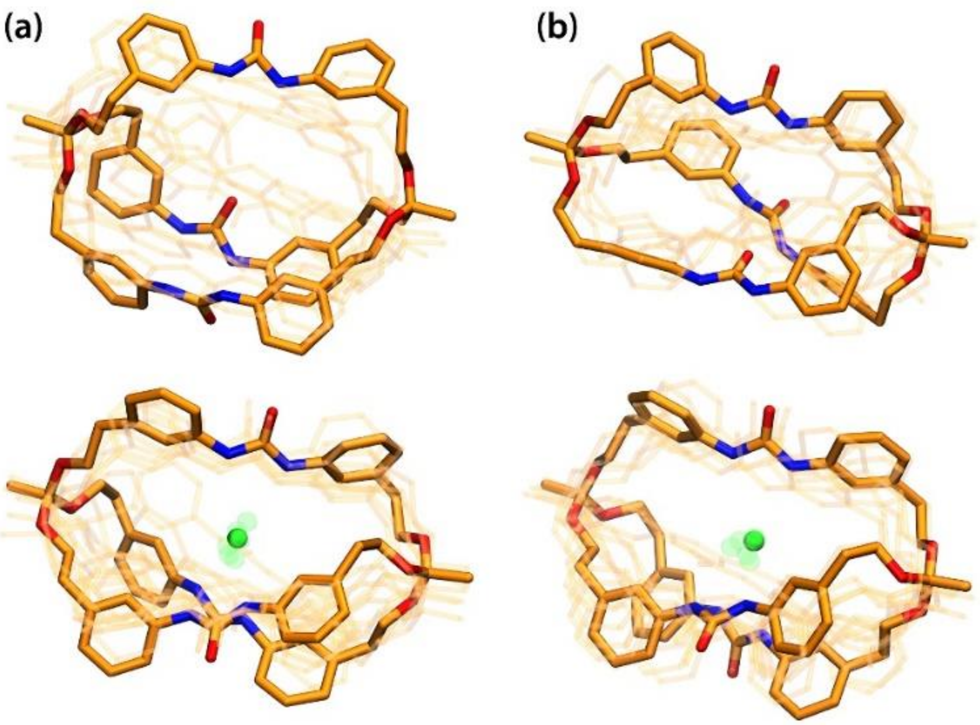

Figure S51: Flexibility of cryptand illustrated by superimposed and smoothed snapshots from μs MD simulations of isolated cryptand (top) and complex with Cl^- (bottom) in pure (a) DMSO and (b) chloroform. Hydrogen atoms are omitted for clarity.

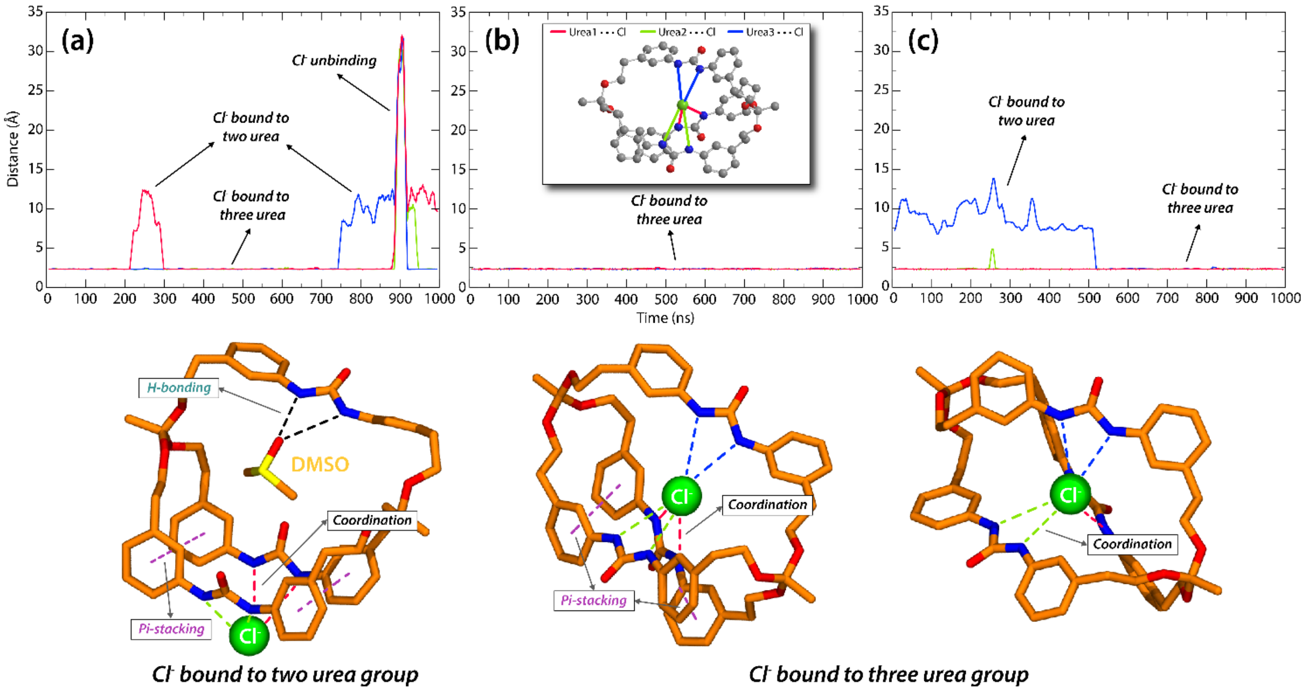

Figure S52: Distance between hydrogen atoms of urea groups and Cl^- calculated from μs simulation of complex in pure (a) DMSO and (b) chloroform and (c) 5:1 (chloroform/DMSO) mixed solvent. Representative snapshots of complex including coordination and hydrogen bonds are shown below panels.

7. Host-Guest Titrations

7.1 General Procedure

General Procedure (E) for ^{1}H NMR titrations of cryptands with anions

Stock solutions of corresponding cryptand and salt in DMSO-d_6 or in $CDCl_3$/DMSO-d_6 (5:1) mixture were prepared and the precise concentration of the solutions was determined by ^{1}H NMR spectroscopy using 1,4-dinitrobenzene or 1,3,5-trimethoxybenzene as internal standard. 600 µL of cryptand stock solution were loaded into an NMR tube with a screw cap containing a septum. 3 µL TEA were added to avoid hydrolysis. During the titration, varying amounts of the salt stock solution and additional cryptand stock solution to keep the concentration of the cryptand constant were added through the septum using Hamilton syringes. The titration was monitored by ^{1}H NMR spectroscopy (400 MHz, 298 K). The data were fitted using Bindfit[10,11] (Fit method: Nelder-Mead, Binding model: 1:1). One representative salt (TPPCl) was titrated in triplicate to obtain a more complete picture of experimental error(s).

Table S1: Association constants (K_a) obtained for the titration of various cryptands with TPPCl, TPPBr, TPPI and $TMANO_3$ (150 mM) in DMSO-d_6 or $CDCl_3$/DMSO-d_6 (5:1) by fitting the titration data according to 1:1 binding model. Fit method: Nelder-Mead. $\boldsymbol{s}/\sqrt{\boldsymbol{n}}$ – standard deviation of the mean, where $\boldsymbol{s}$ - standard deviation, $\boldsymbol{n}$ - number of measurements; $\boldsymbol{t}_{(0.05,2)} \times \boldsymbol{s}/\sqrt{\boldsymbol{n}}$ – 95% confidence interval, where $\boldsymbol{t}_{(0.05,2)}$ – Student's t at 95% confidence level. *Fit error obtained using Bindfit.

	***K*a** [M^{-1}]	**Fit error*** [M^{-1}]	**Avg. *K*a** [M^{-1}]	**[Host]$_0$** [mM]	$\boldsymbol{s}/\sqrt{\boldsymbol{n}}$ [M^{-1}]	$\boldsymbol{t}_{(0.05,2)} \times \boldsymbol{s}/\sqrt{\boldsymbol{n}}$ [M^{-1}]
***o*-Me$_2$-ur-C2** (in DMSO-d_6)						
	41	2		4.7		
TPPCl	37.2	0.4	37	8.7	2.0	± 8.7
	34	1		6.0		
TPPBr	16	1		7.0		
TPPI	10	1		10.1		
$TMANO_3$	8.6	0.3		10.1		
***o*-Me$_2$-ur-C3** (in DMSO-d_6)						
TPPCl	27	1		11.0		
***o*-Me$_2$-ur-C4** (in DMSO-d_6)						
TPPCl	17	1		6.0		
***o*-H$_2$-ur-C4** (in DMSO-d_6)						
TPPCl	32.0	0.4		4.1		
***o*-Me$_2$-ur-C2** (in 5:1 $CDCl_3$/DMSO-d_6)						
TPPCl	286	23		5.7		

Table S2: Comparison of different binding models used to fit the representative titration data shown in Figure S54. Solvent: DMSO-d_6 containing up to 10% water. Fit method: Nelder-Mead. [***o*-Me₂-ur-C2**]$_0$: 4.7 mM; [TPPCl]$_0$: 150 mM.

	1:1 model	1:2 model		2:1 model	
	K_a [M^{-1}]	K_1 [M^{-1}]	K_2 [M^{-1}]	K_1 [M^{-1}]	K_2 [M^{-1}]
	41	57	-6.0	0.6	5815
Error	4%	4%	-16%	16%	26%
RMS	$9\cdot10^{-3}$	$6\cdot10^{-3}$		$7\cdot10^{-3}$	
Covariance	$2\cdot10^{-3}$	$9\cdot10^{-4}$		$1\cdot10^{-3}$	

Comment: Fitting the titration data according to 1:2 or 2:1 binding model does not improve the quality of fit. Even though RMS and covariance are slightly reduced, the 1:2 binding model does not give an physically plausible value for K_2. Moreover, the 2:1 model produces highly implausible binding constants and both models do not allow to obtain reproducible (with an acceptable standard deviation) binding constants for the three different titration experiments. We conclude that binding of Cl^- to ***o*-Me$_2$-ur-C2** can be best described by the 1:1 model and that this stoichiometry therefore dominates in solution.

Table S3: Links to raw data, calculated fits and statistical information for the titrations.

	"open data" hyperlinks
o*-Me$_2$-ur-C2** (in DMSO-d_6) ***TPPCl	http://app.supramolecular.org/bindfit/view/442e7e14-12ab-4beb-81fb-0c5b3837e0ef<br>http://app.supramolecular.org/bindfit/view/db903da5-d3a3-48a0-a019-6881ee2bcdd4<br>http://app.supramolecular.org/bindfit/view/6a2d242f-dd24-4e7b-aba2-9478b7553408
TPPBr	http://app.supramolecular.org/bindfit/view/92a96283-d75d-4825-a3aa-b5f04fddf551
TPPI	http://app.supramolecular.org/bindfit/view/17cd159d-b9e1-4f70-9912-c08b36a810c0
TMANO$_3$	http://app.supramolecular.org/bindfit/view/d2624ac2-b103-4e12-907a-1faab5d569f5
o*-Me$_2$-ur-C3** (in DMSO-d_6) ***TPPCl	http://app.supramolecular.org/bindfit/view/11942544-251f-4860-9040-da2b3940d5ed
o*-Me$_2$-ur-C4** (in DMSO-d_6) ***TPPCl	http://app.supramolecular.org/bindfit/view/876cf34a-1b6d-44e9-be35-2d478bfec3e9
o*-H$_2$-ur-C4** (in DMSO-d_6) ***TPPCl	http://app.supramolecular.org/bindfit/view/cc55d0aa-3967-43b6-b422-fdf3c5696538
o*-Me$_2$-ur-C2** (in 5:1 $CDCl_3$/DMSO-d_6) ***TPPCl	http://app.supramolecular.org/bindfit/view/7e8484b9-8fcc-483b-9a3c-1ba46a477010

7.2 NMR Titration Data, Isotherms and Residuals

***o*-Me₂-ur-C2** *and TPPCl in DMSO-d₆*

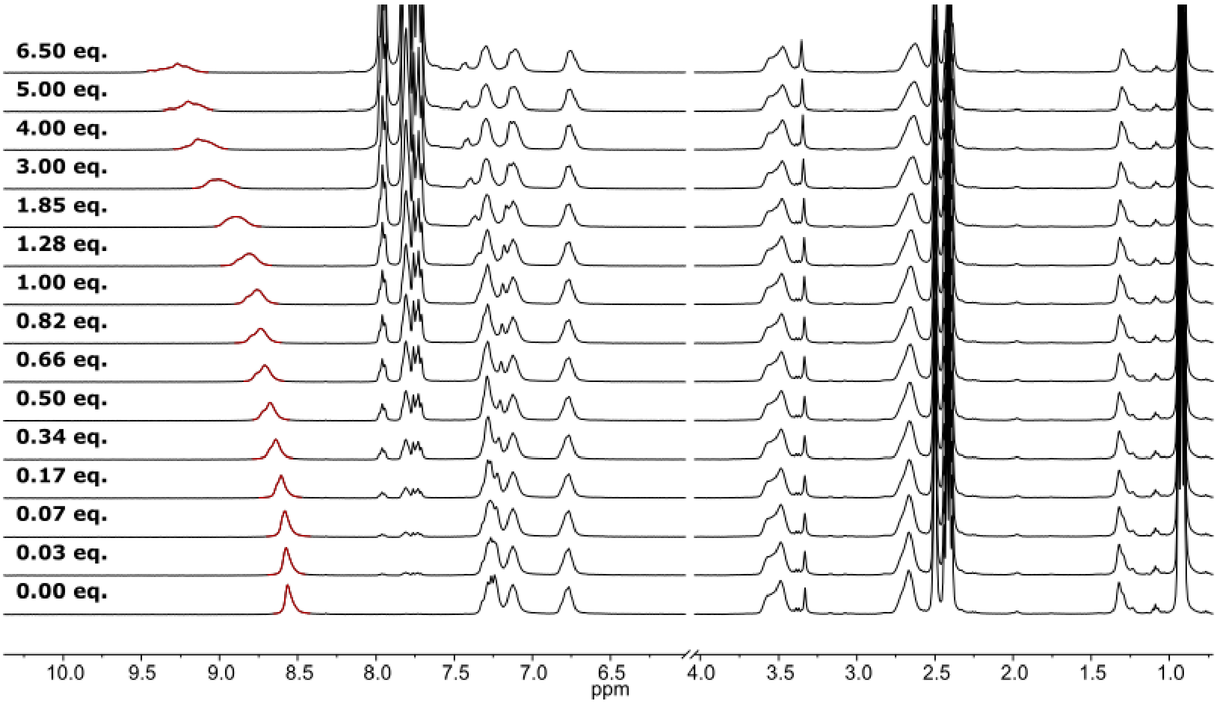

Figure S53: Representative ^{1}H NMR (400 MHz, 298 K, DMSO-d$_6$) stack plot for a titration of ***o*-Me$_2$-ur-C2** (8.7 mM) with TPPCl (150 mM).

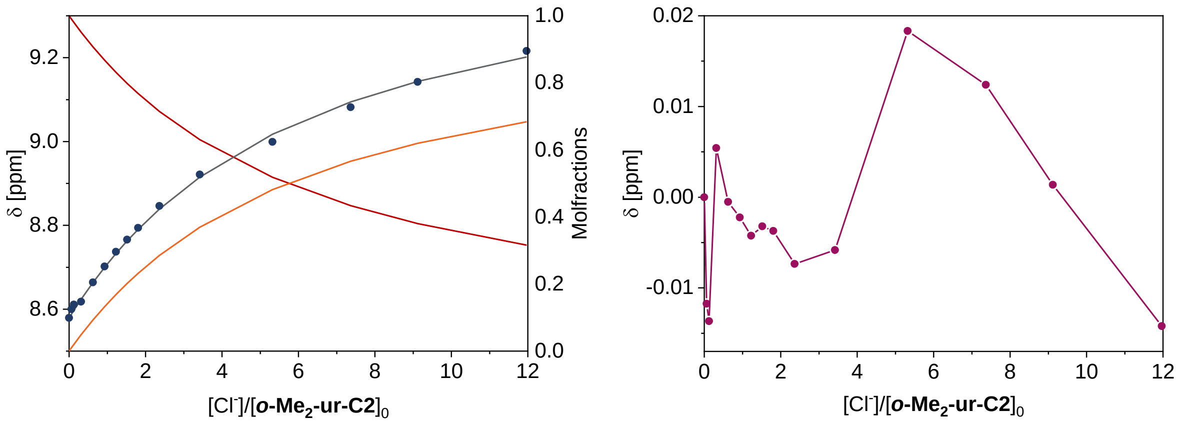

Figure S54: Left: Binding isotherm and mole fractions of species for titration of ***o*-Me$_2$-ur-C2** (4.7 mM) with TPPCl (150 mM) in DMSO-d$_6$ at 298 K. Blue dots: Experimental data points; Grey line: Fitting curve according to 1:1 binding model; red curve: mole fraction of host; orange curve: mole fraction of host-guest complex. Right: Residual plot.

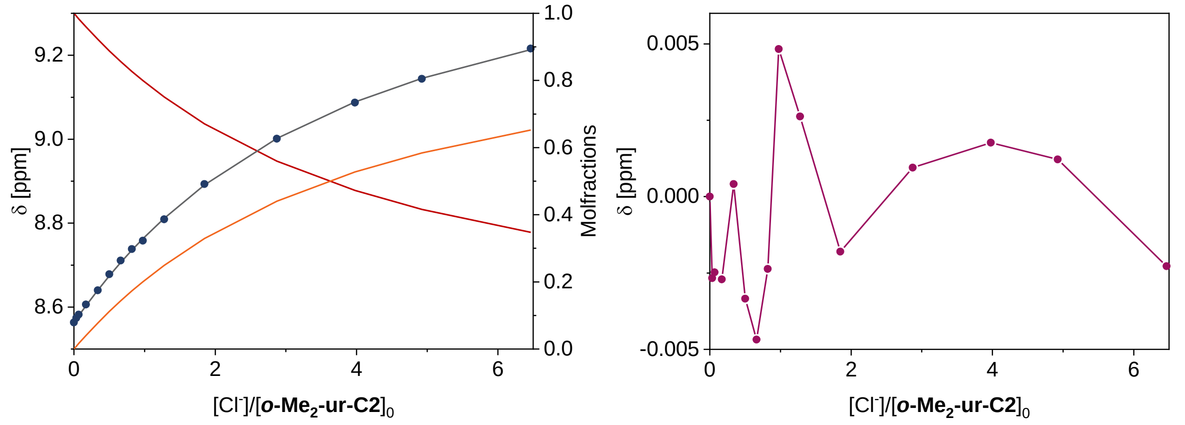

Figure S55: Left: Binding isotherm and mole fractions of species for titration of ***o*-Me₂-ur-C2** (8.7 mM) with TPPCl (150 mM) in DMSO-d_6 at 298 K. Blue dots: Experimental data points; Grey line: Fitting curve according to 1:1 binding model; red curve: mole fraction of host; orange curve: mole fraction of host-guest complex. Right: Residual plot.

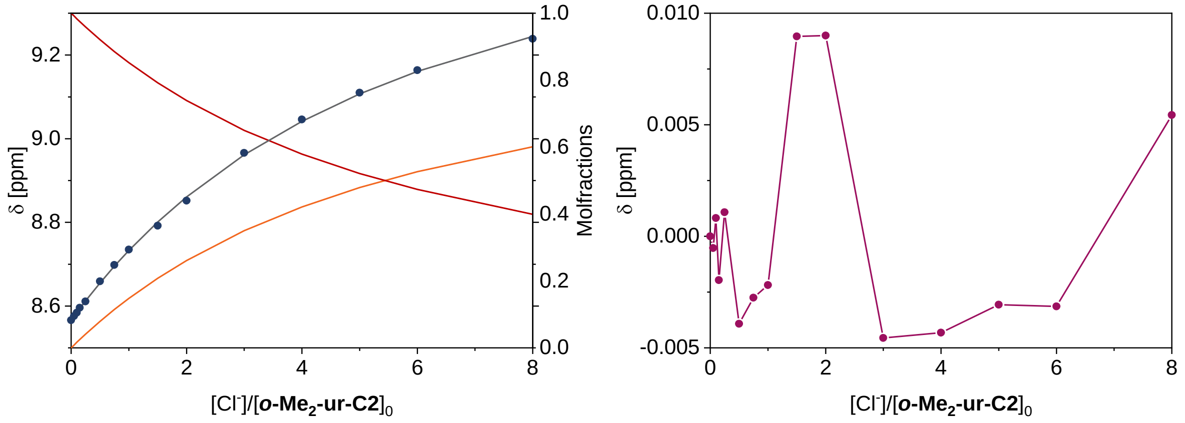

Figure S56: Left: Binding isotherm and mole fractions of species for titration of ***o*-Me₂-ur-C2** (6.0 mM) with TPPCl (150 mM) in DMSO-d_6 at 298 K. Blue dots: Experimental data points; Grey line: Fitting curve according to 1:1 binding model; red curve: mole fraction of host; orange curve: mole fraction of host-guest complex. Right: Residual plot.

***o*-Me$_2$-ur-C2** *and TPPBr in DMSO-d$_6$*

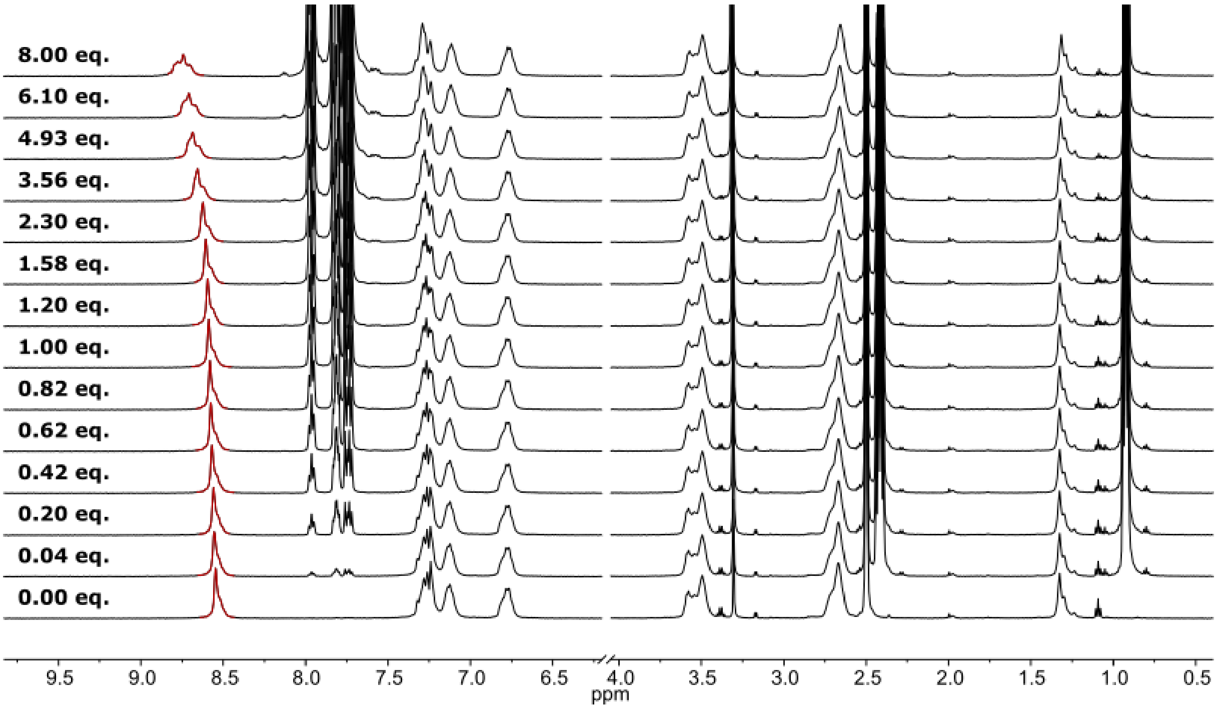

Figure S57: ^{1}H NMR (400 MHz, 298 K, DMSO-d$_6$) stack plot for the titration of ***o*-Me$_2$-ur-C2** (7.0 mM) with TPPBr (150 mM).

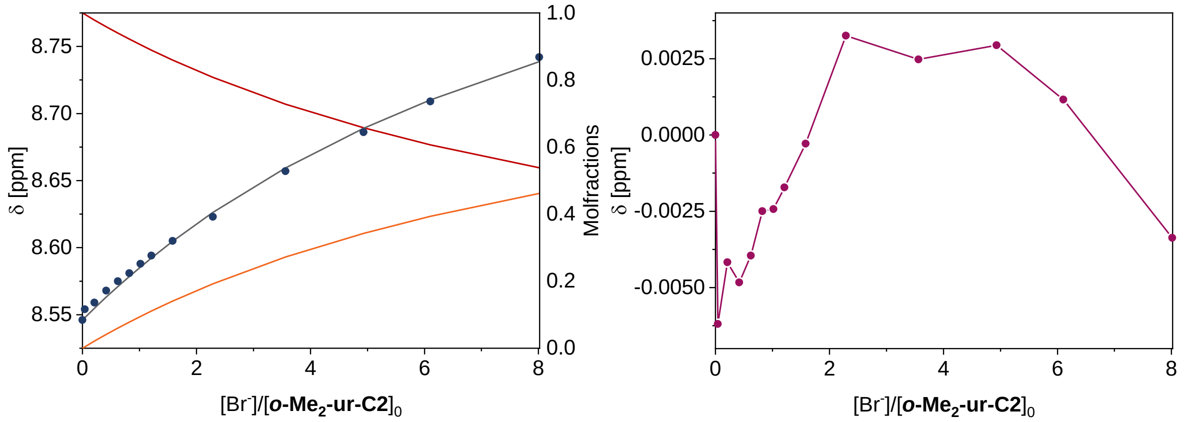

Figure S58: Left: Binding isotherm and mole fractions of species for titration of ***o*-Me$_2$-ur-C2** (7.0 mM) with TPPBr (150 mM) in DMSO-d$_6$ at 298 K. Blue dots: Experimental data points; Grey line: Fitting curve according to 1:1 binding model; red curve: mole fraction of host; orange curve: mole fraction of host-guest complex. Right: Residual plot. The residuals plot gives impression of a systematic deviation. Therefore, we performed fits for 2:1 and 1:2 binding model, but this showed no improved data.

***o*-Me$_2$-ur-C2** *and TPPI in DMSO-d$_6$*

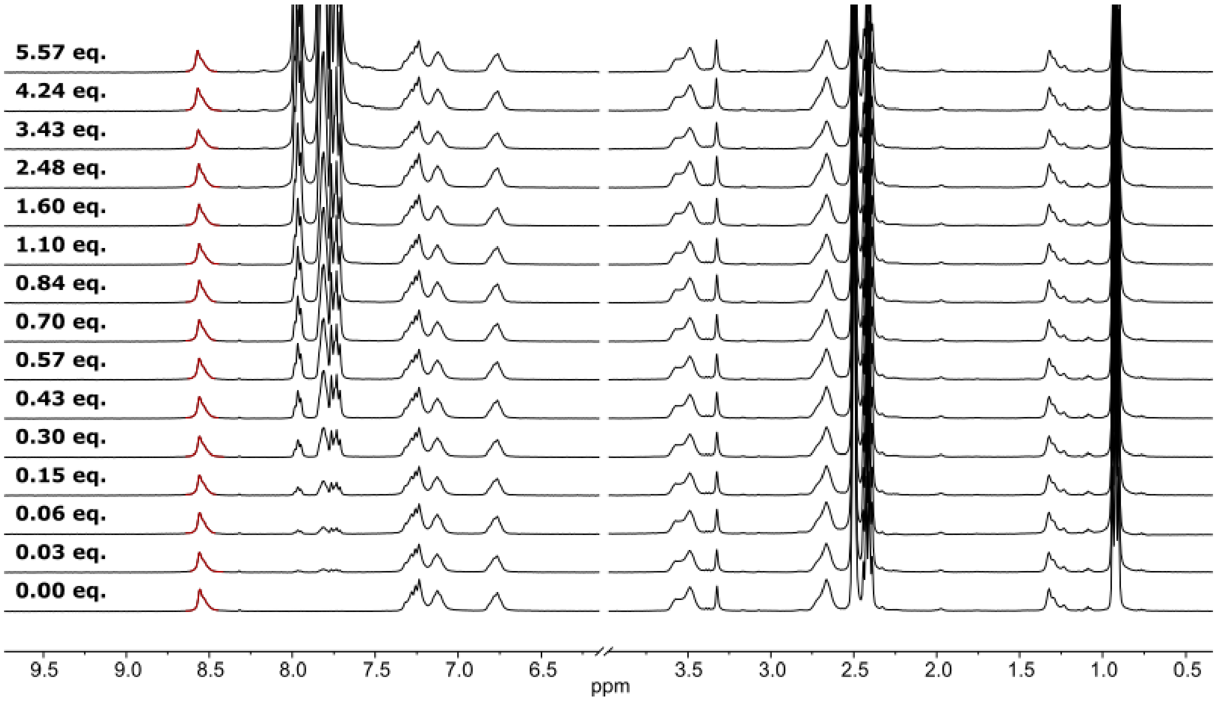

Figure S59: ^{1}H NMR (400 MHz, 298 K, DMSO-d$_6$) stack plot for the titration of ***o*-Me$_2$-ur-C2** (10.1 mM) with TPPI (150 mM).

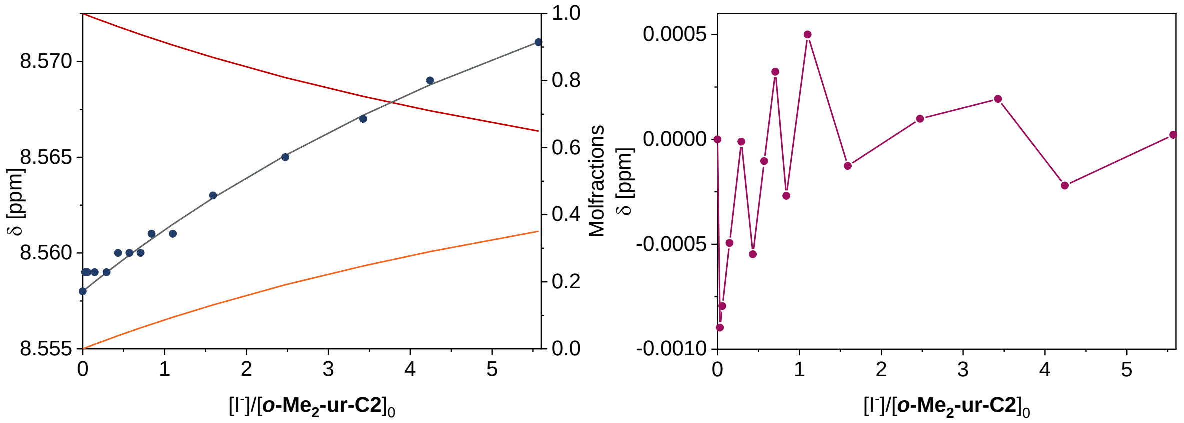

Figure S60: Left: Binding isotherm and mole fractions of species for titration of ***o*-Me$_2$-ur-C2** (10.1 mM) with TPPI (150 mM) in DMSO-d$_6$ at 298 K. Blue dots: Experimental data points; Grey line: Fitting curve according to 1:1 binding model; red curve: mole fraction of host; orange curve: mole fraction of host-guest complex. Right: Residual plot.

***o*-Me₂-ur-C2** *and TMANO₃ in DMSO-d₆*

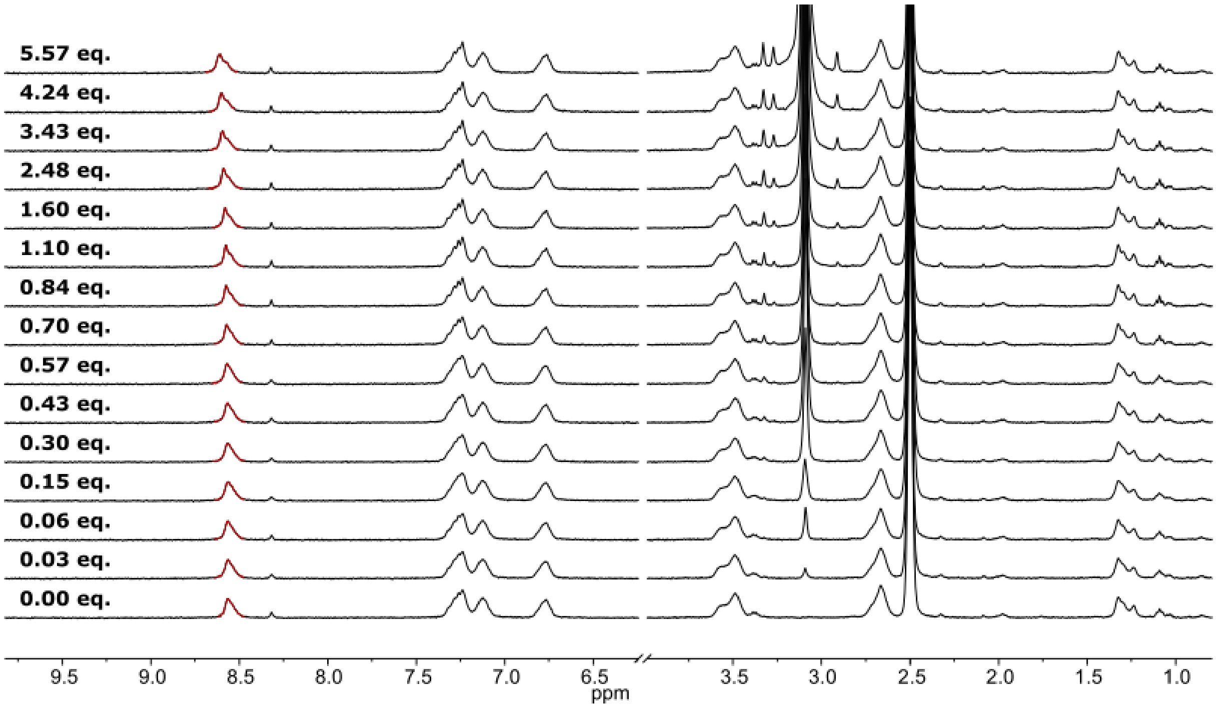

Figure S61: ^{1}H NMR (400 MHz, 298 K, DMSO-d_6) stack plot for the titration of ***o*-Me$_2$-ur-C2** (10.1 mM) with TMANO$_3$ (150 mM).

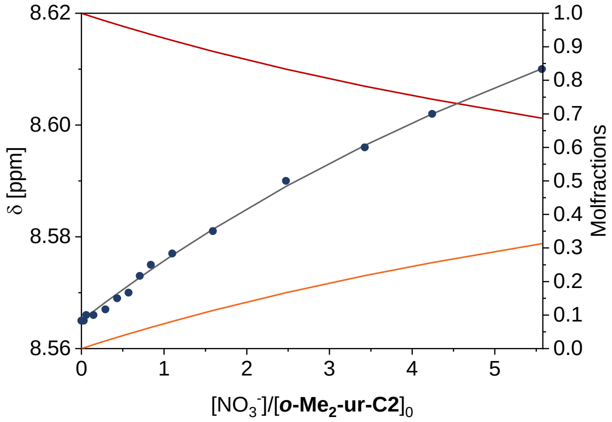

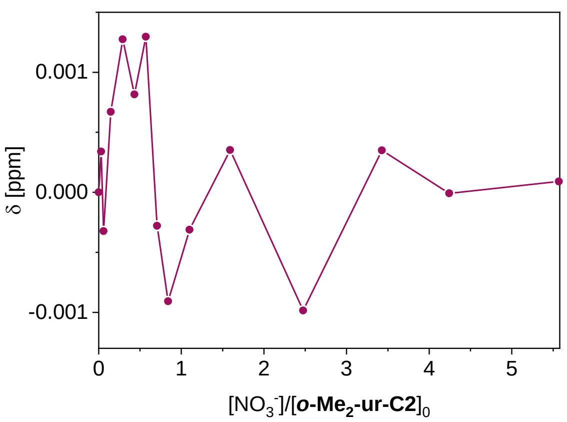

Figure S62: Left: Binding isotherm and mole fractions of species for titration of ***o*-Me$_2$-ur-C2** (10.1 mM) with TMANO$_3$ (150 mM) in DMSO-d_6 at 298 K. Blue dots: Experimental data points; Grey line: Fitting curve according to 1:1 binding model; red curve: mole fraction of host; orange curve: mole fraction of host-guest complex. Right: Residual plot.

***o*-Me$_2$-ur-C3** *and TPPCl in DMSO-d$_6$*

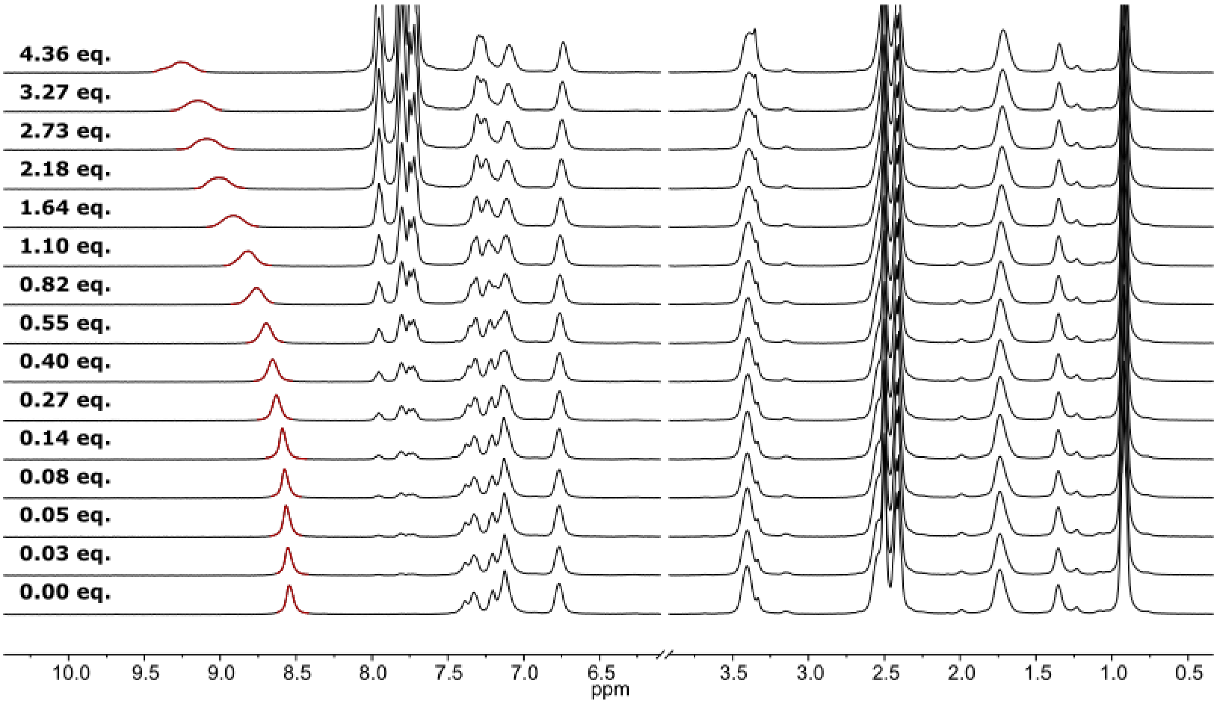

Figure S63: ^{1}H NMR (400 MHz, 298 K, DMSO-d$_6$) stack plot for the titration of ***o*-Me$_2$-ur-C3** (11.0 mM) with TPPCl (150 mM).

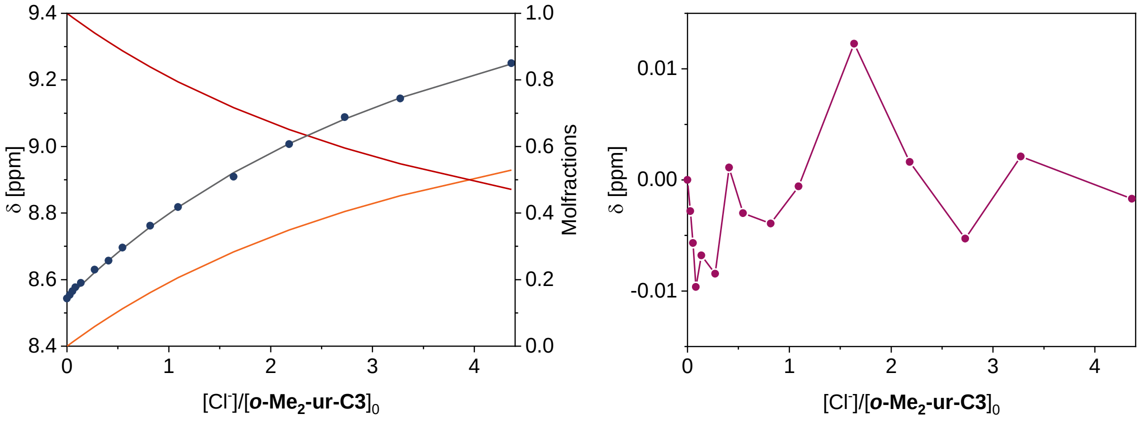

Figure S64: Left: Binding isotherm and mole fractions of species for titration of ***o*-Me$_2$-ur-C3** (11.0 mM) with TPPCl (150 mM) in DMSO-d$_6$ at 298 K. Blue dots: Experimental data points; Grey line: Fitting curve according to 1:1 binding model; red curve: mole fraction of host; orange curve: mole fraction of host-guest complex. Right: Residual plot.

***o*-Me$_2$-ur-C4** *and TPPCl in DMSO-d$_6$*

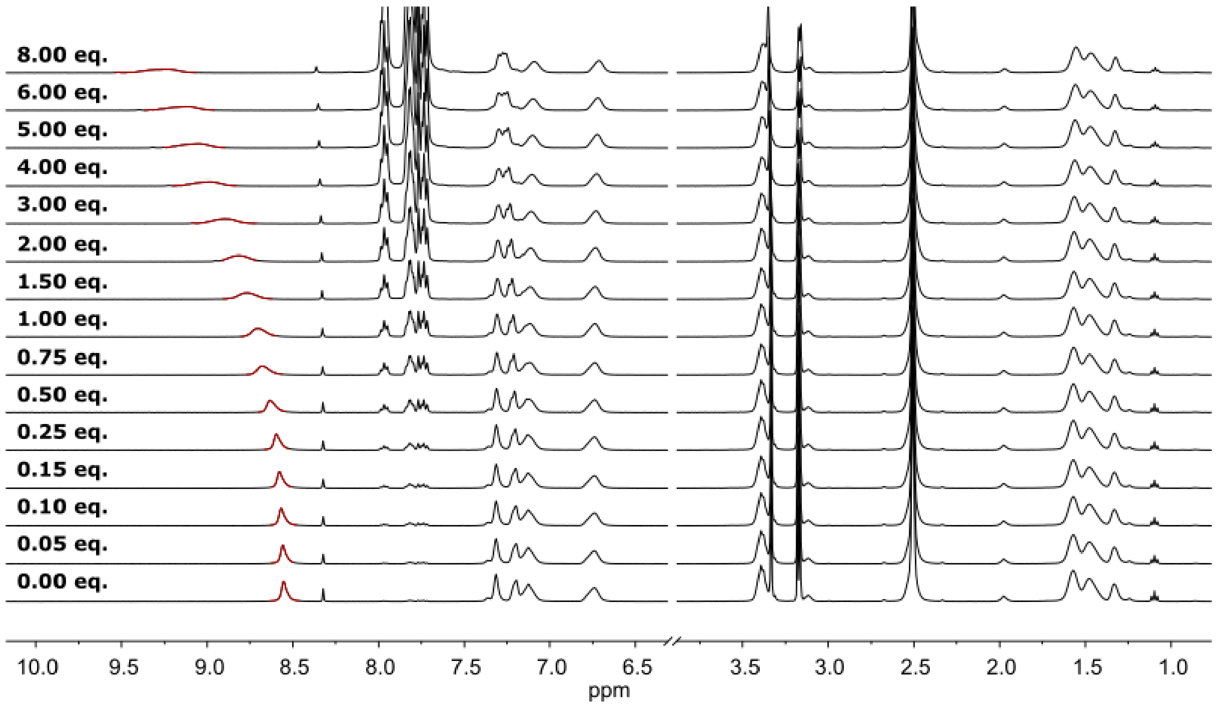

Figure S65: ^{1}H NMR (400 MHz, 298 K, DMSO-d$_6$) stack plot for the titration of ***o*-Me$_2$-ur-C4** (6.0 mM) with TPPCl (150 mM).

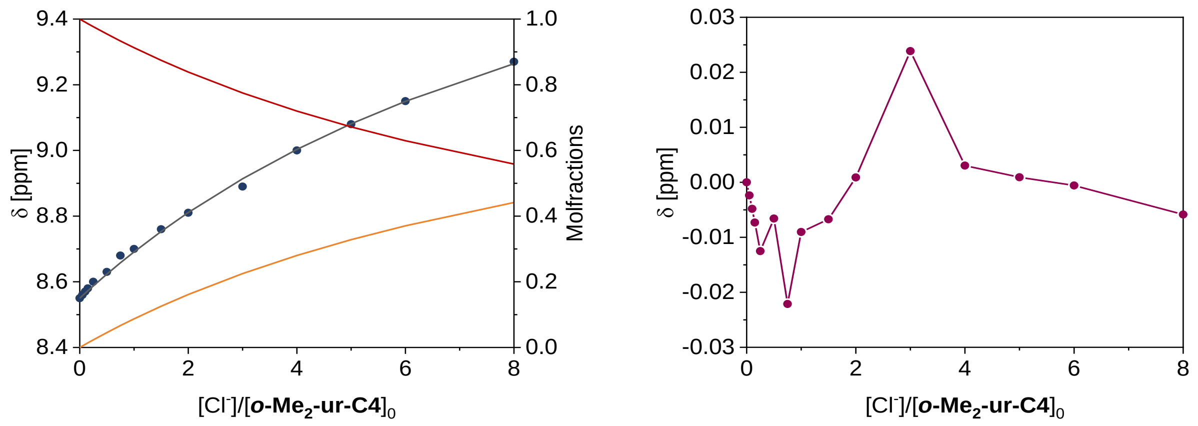

Figure S66: Left: Binding isotherm and mole fractions of species for titration of ***o*-Me$_2$-ur-C4** (6.0 mM) with TPPCl (150 mM) in DMSO-d$_6$ at 298 K. Blue dots: Experimental data points; Grey line: Fitting curve according to 1:1 binding model; red curve: mole fraction of host; orange curve: mole fraction of host-guest complex. Right: Residual plot.

***o*-H$_2$-ur-C4** *and TPPCl in DMSO-d$_6$*

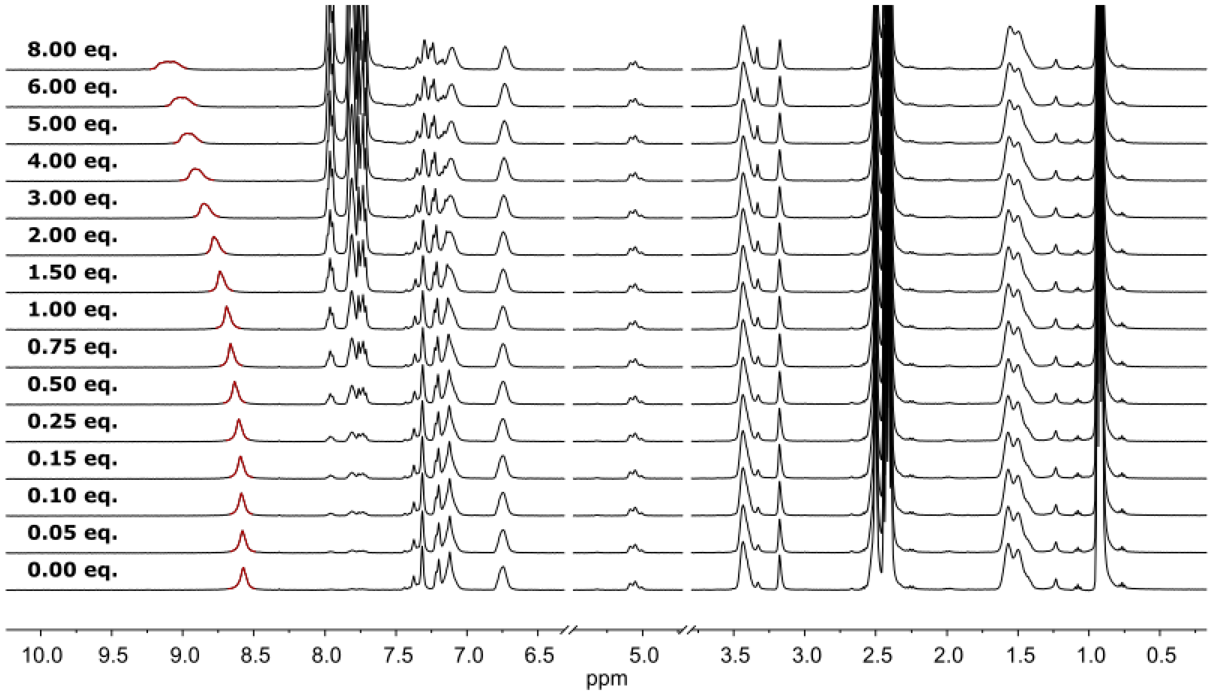

Figure S67: ^{1}H NMR (400 MHz, 298 K, DMSO-d$_6$) stack plot for the titration of ***o*-H$_2$-ur-C4** (4.1 mM) with TPPCl (150 mM).

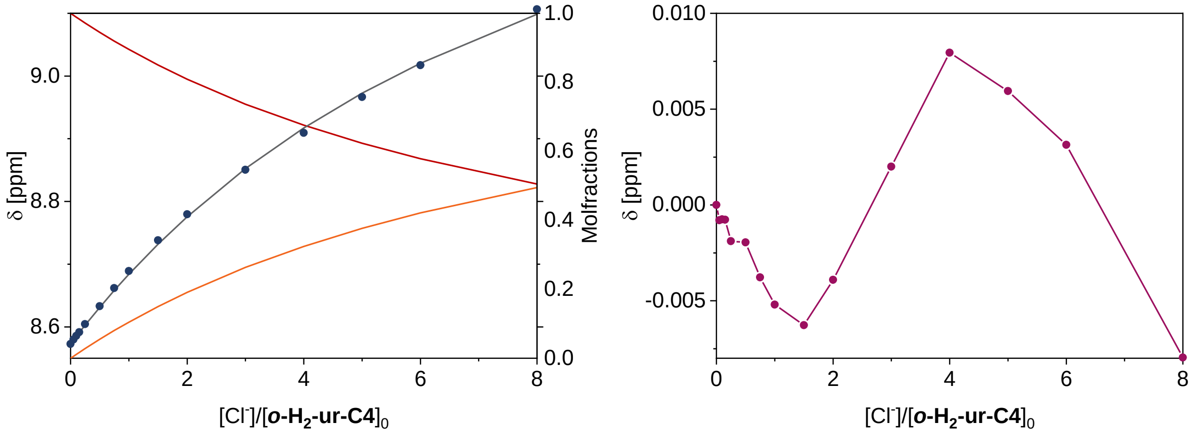

Figure S68: Left: Binding isotherm and mole fractions of species for titration of ***o*-H$_2$-ur-C4** (4.1 mM) with TPPCl (150 mM) in DMSO-d$_6$ at 298 K. Blue dots: Experimental data points; Grey line: Fitting curve according to 1:1 binding model; red curve: mole fraction of host; orange curve: mole fraction of host-guest complex. Right: Residual plot.

***o*-Me$_2$-ur-C2** *and TPPCl in CDCl$_3$/DMSO-d$_6$ (5:1)*

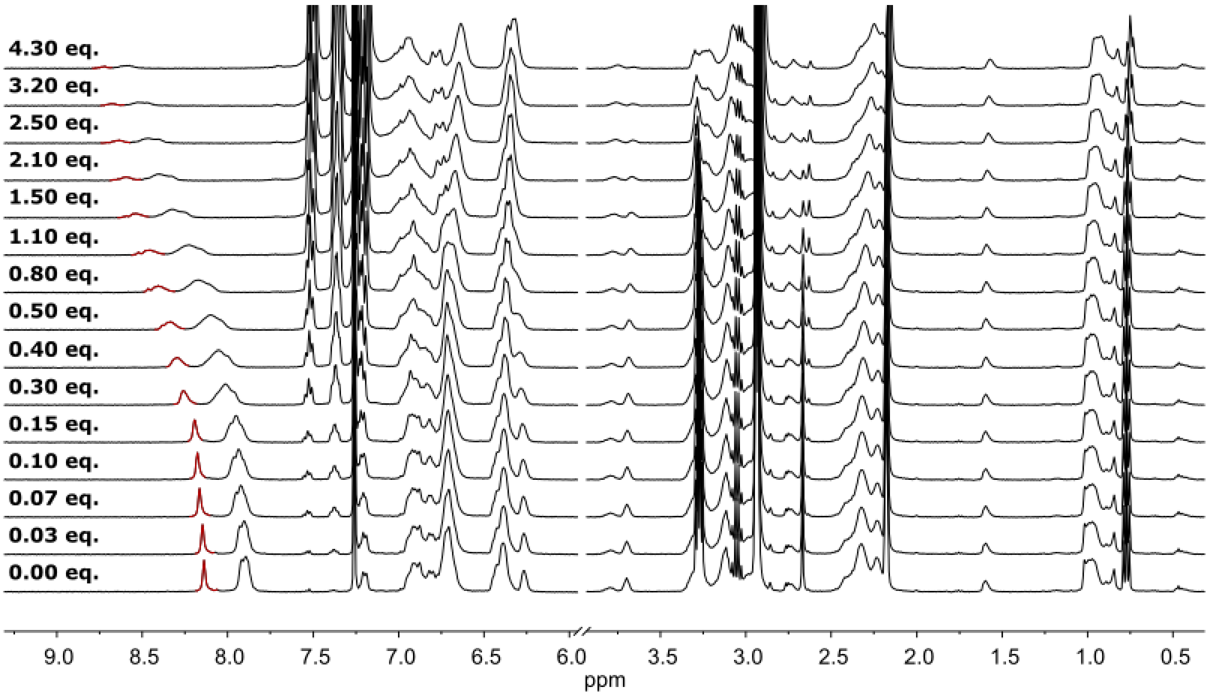

Figure S69: ^{1}H NMR (400 MHz, 298 K, CDCl$_3$/DMSO-d$_6$ (5:1)) stack plot for the titration of ***o*-Me$_2$-ur-C2** (5.7 mM) with TPPCl (150 mM). Left NH signal at 8.13 ppm was chosen for fit due to the more regular shape.

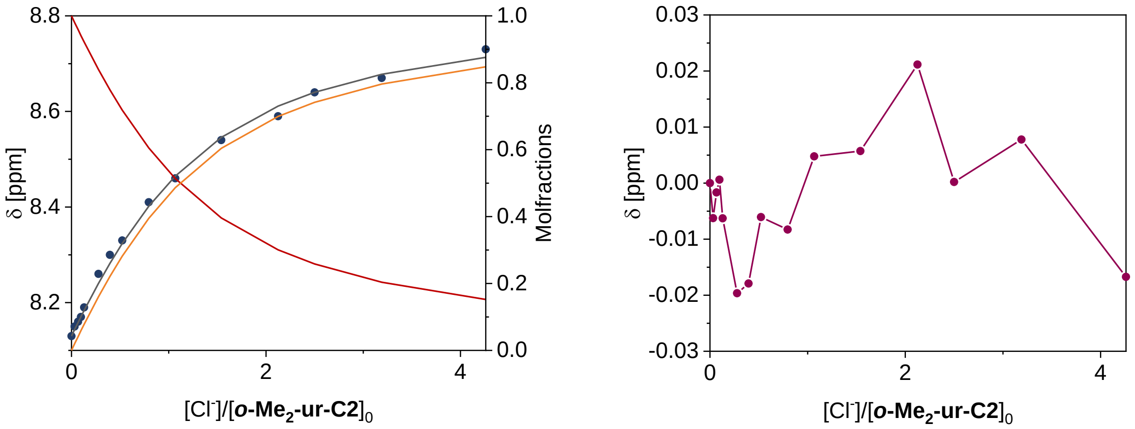

Figure S70: Left: Binding isotherm and mole fractions of species for titration of ***o*-Me$_2$-ur-C2** (5.7 mM) with TPPCl (150 mM) in CDCl$_3$/DMSO-d$_6$ (5:1) at 298 K. Blue dots: Experimental data points; Grey line: Fitting curve according to 1:1 binding model; red curve: mole fraction of host; orange curve: mole fraction of host-guest complex. Right: Residual plot.

8. High resolution ESI-MS/MS measurements

Collision energy is given as center-of-mass frame (E_{com}) in dependence on masses of collision gas (N_2) and precursor ion, and the measured laboratory collision energy (E_{lab}) according to Eqn. (1):

$$E_{com} = E_{lab} \cdot \frac{m_{N_2}}{m_{N_2} + m_{Precursor}} \qquad \text{Eqn. (1)}$$

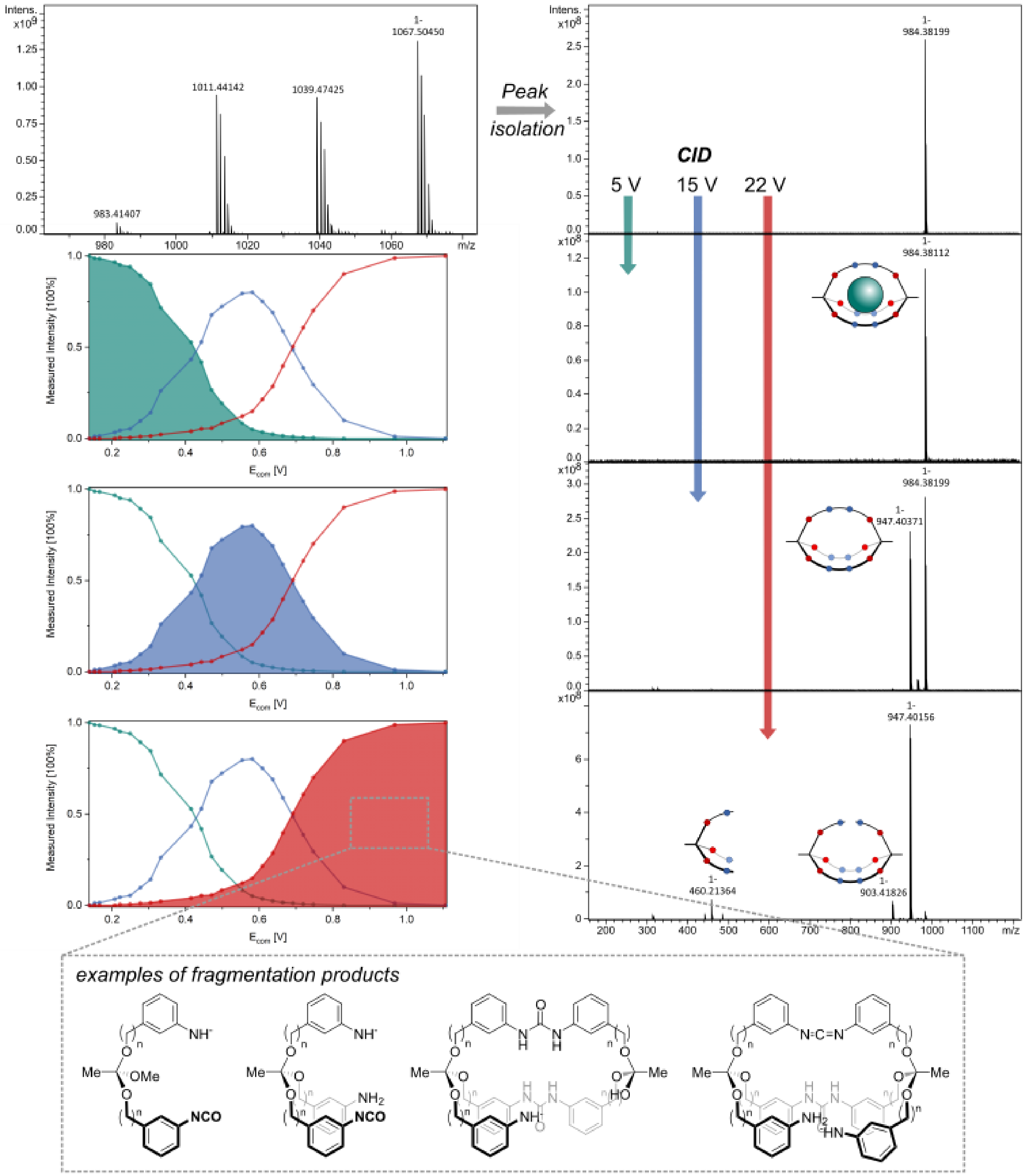

Figure S71: Representative HR-ESI-MS/MS spectra indicative for the approach. After isolation of the product peak (**[Cl⁻⊂*o*-Me$_2$-ur-C2]**), increasing the voltage leads to release of the Chloride ion (decrease of **[Cl⁻⊂*o*-Me$_2$-ur-C2]**, green curve, and formation of ***o*-Me$_2$-ur-C2**, blue curve) and subsequently, cryptand degradation (red curve, inlet: examples of degradation products).

Table S4: Ratio of diols used in experiment and corresponding ratio of cryptates detected by ESI-HRMS spectrometry.

Diol Ratio		***Measured Intensity of [Cl^{-}⊂o-Me_2-ur-X]PPh_4^{+} / %***					
**C1:C2**	**C2:C3**	**X = C1$_2$C2**	**C1C2$_2$**	**C2**	**C2$_2$C3**	**C2C3$_2$**	**C3**
	**1:2**			traces	30	29	41
	**1.5:1.5**			traces	37	46	16
	**2:1**			23	54	17	6
**1.5:1.5**		60	40	-			

9. NMR Spectra

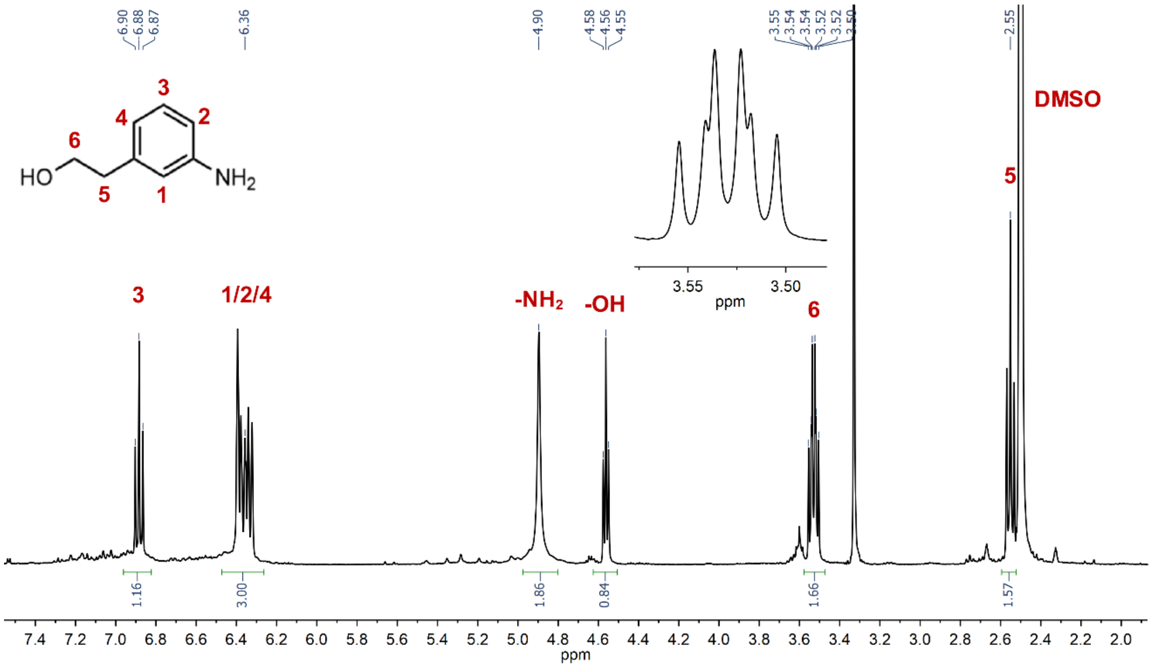

Figure S72: ^{1}H NMR (DMSO-d_6, 400 MHz, 293 K) spectrum of compound (**S2**).

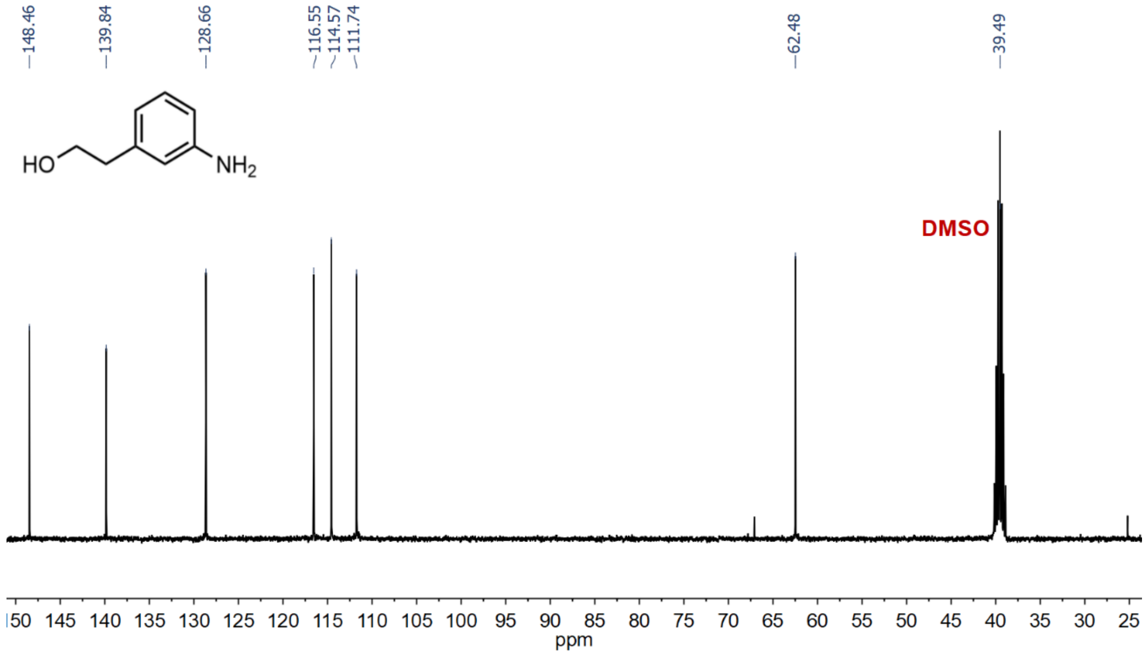

Figure S73: ^{13}C NMR (DMSO-d_6, 101 MHz, 293 K) spectrum of compound (**S2**).

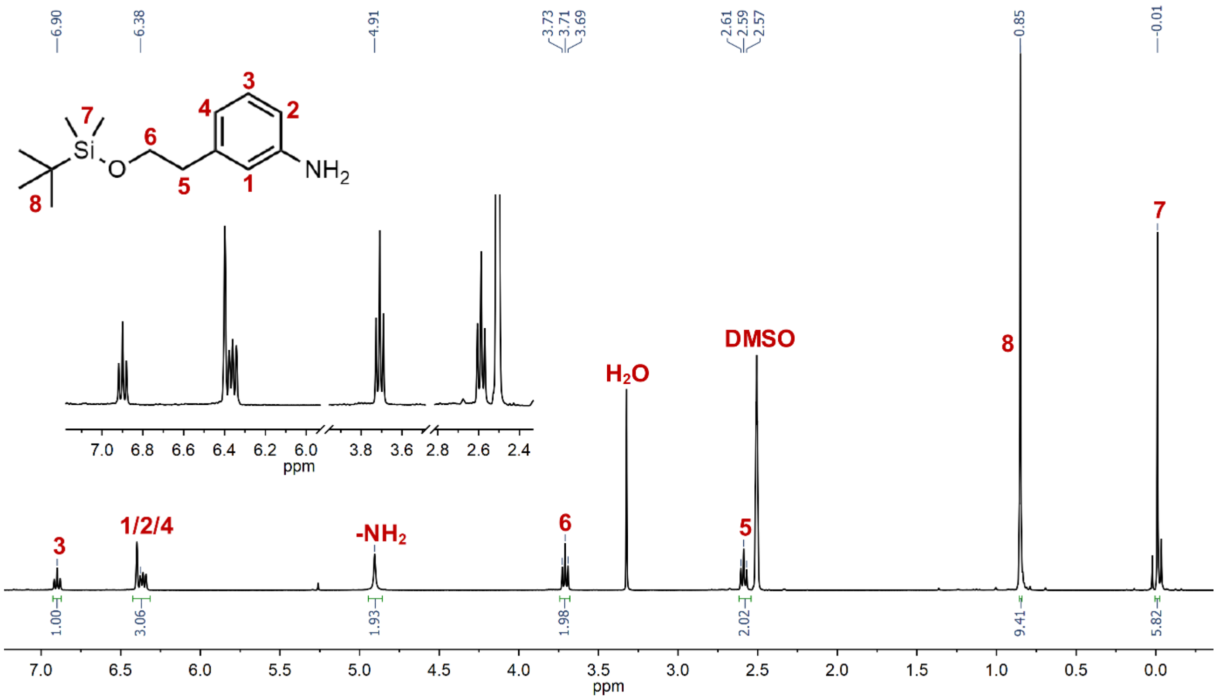

Figure S74: ^{1}H NMR (DMSO-d_6, 400 MHz, 293 K) spectrum of compound (**S3**).

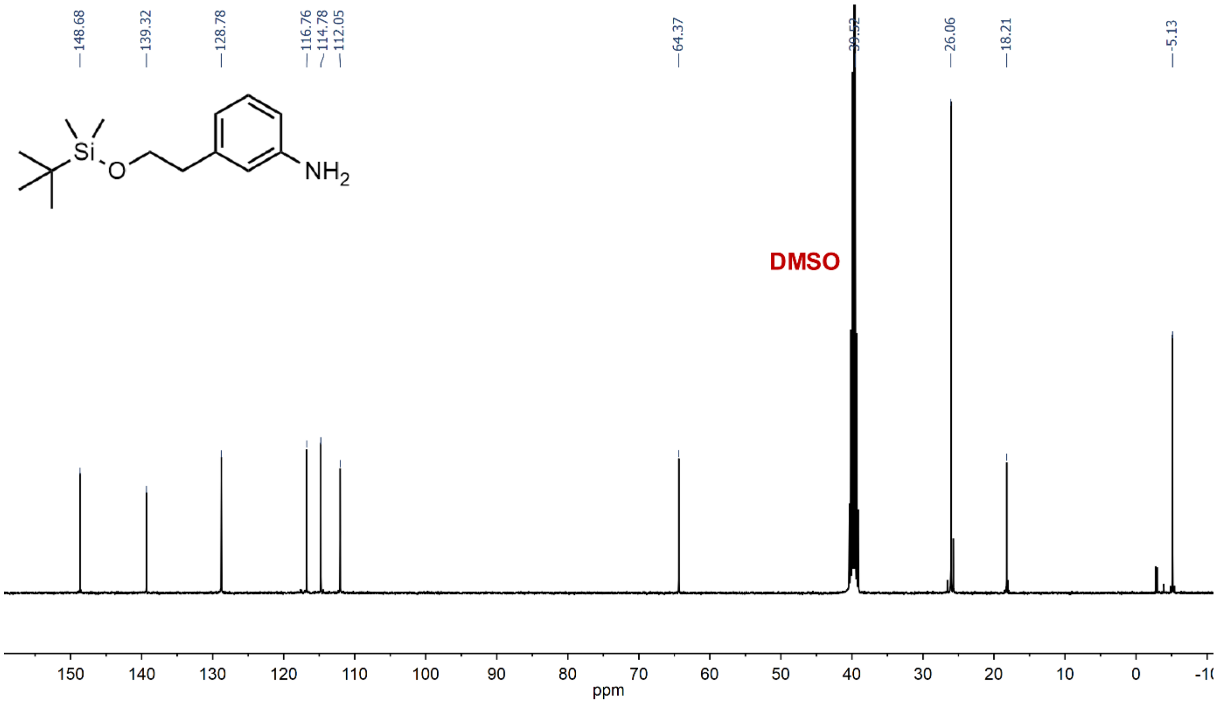

Figure S75: ^{13}C NMR (DMSO-d_6, 101 MHz, 293 K) spectrum of compound (**S3**).

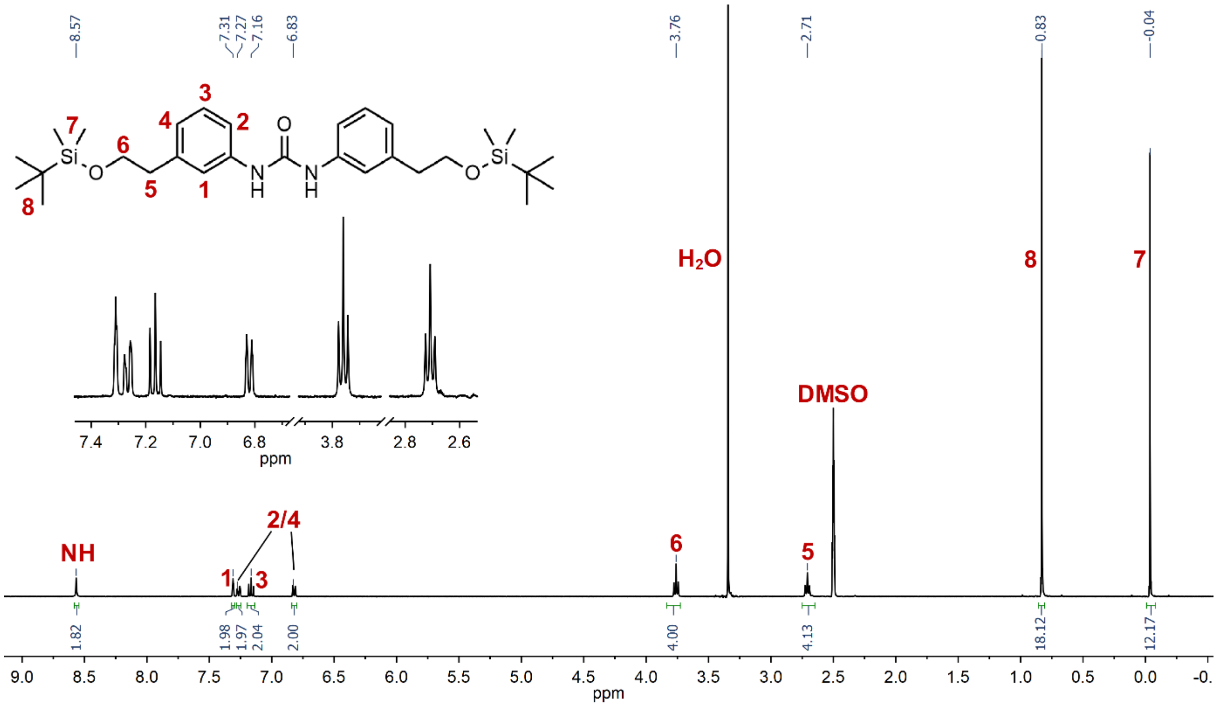

Figure S76: ^{1}H NMR (DMSO-d$_6$, 400 MHz, 293 K) spectrum of compound (**S4**).

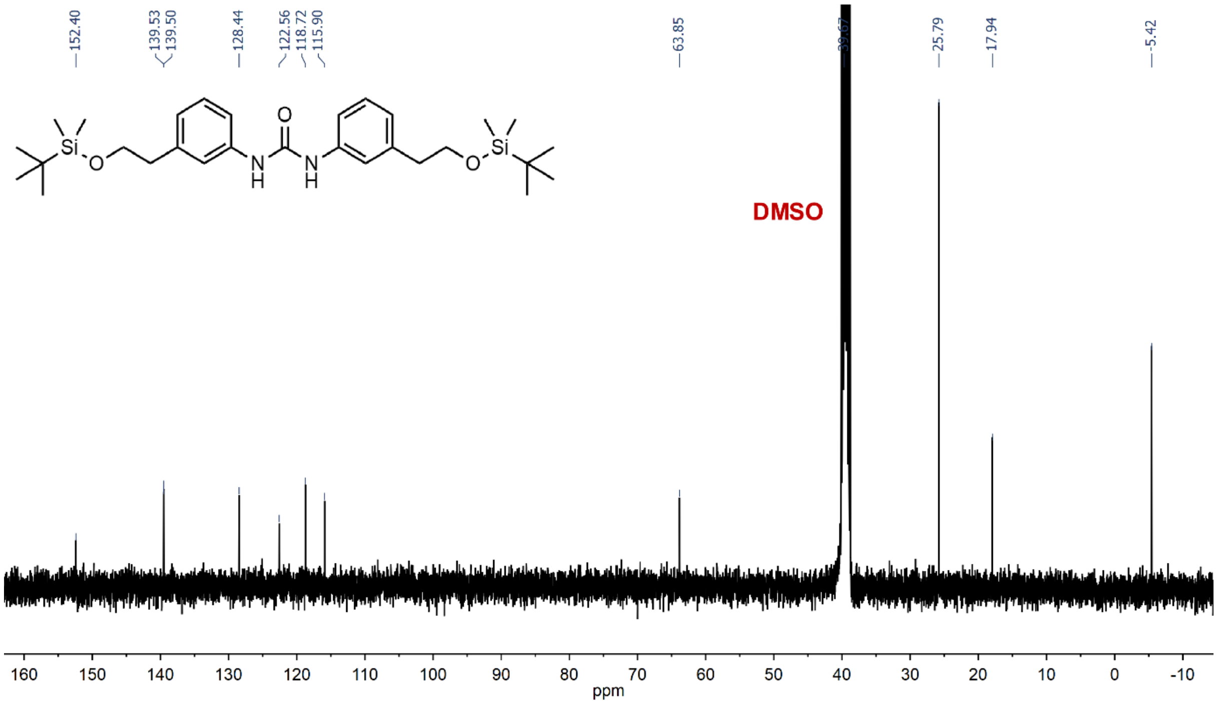

Figure S77: ^{13}C NMR (DMSO-d$_6$, 101 MHz, 293 K) spectrum of compound (**S4**).

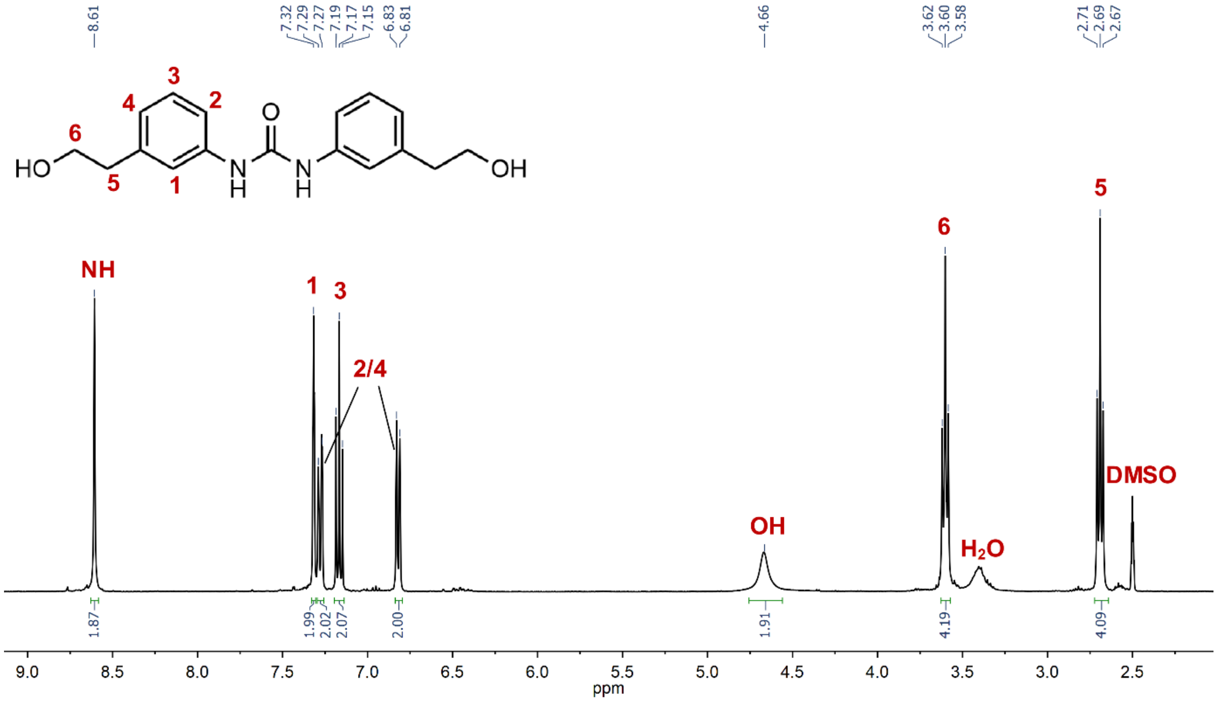

Figure S78: ^{1}H NMR (DMSO-d$_6$, 400 MHz, 293 K) spectrum of compound (**1**).

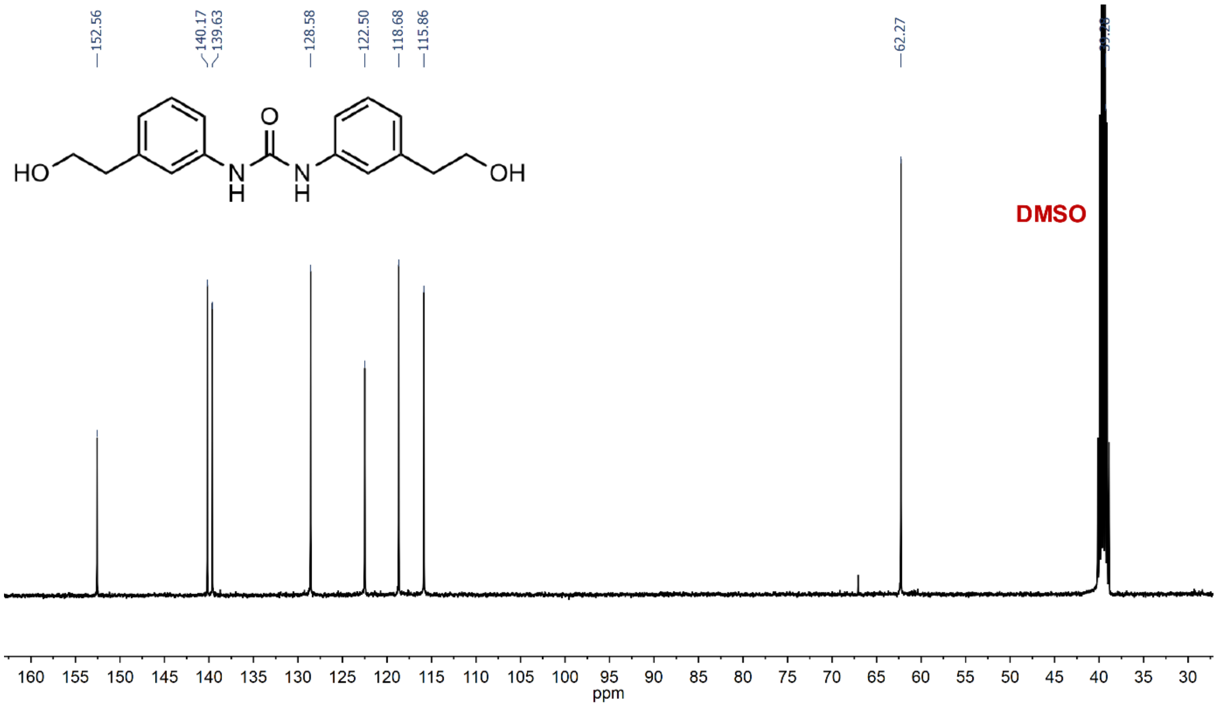

Figure S79: ^{13}C NMR (DMSO-d$_6$, 101 MHz, 293 K) spectrum of compound (**1**).

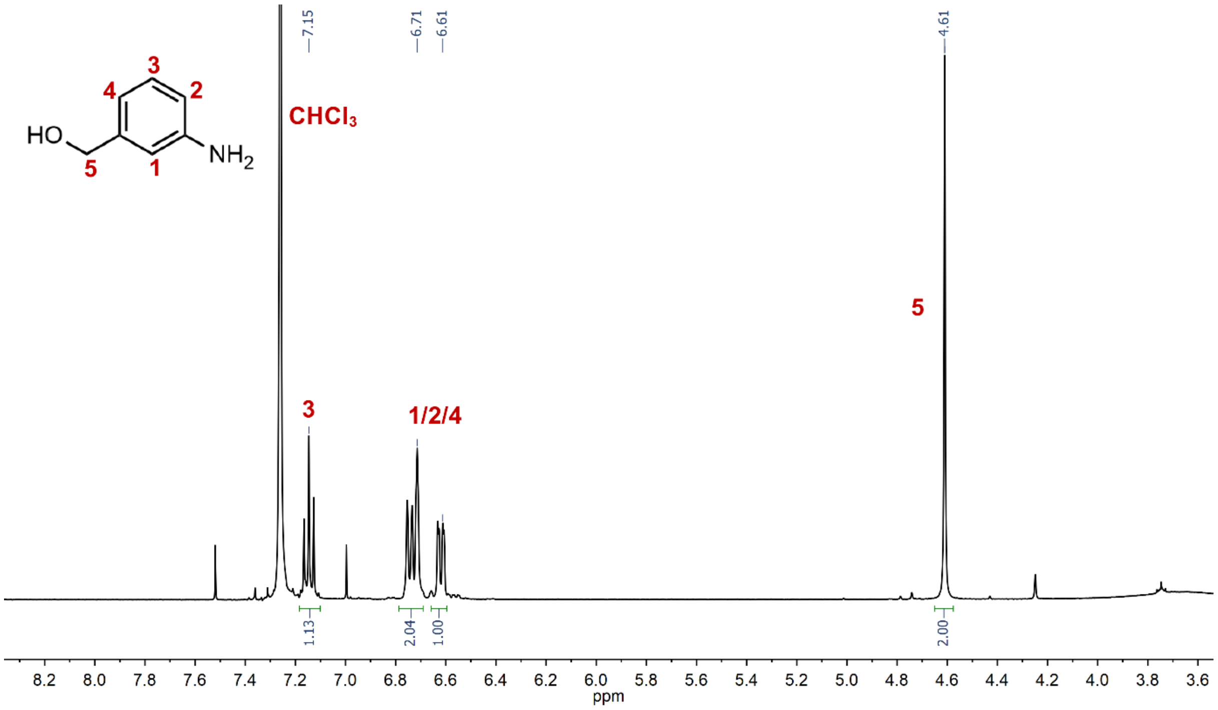

Figure S80: ^{1}H NMR ($CDCl_3$, 400 MHz, 293 K) spectrum of compound (**S6**).

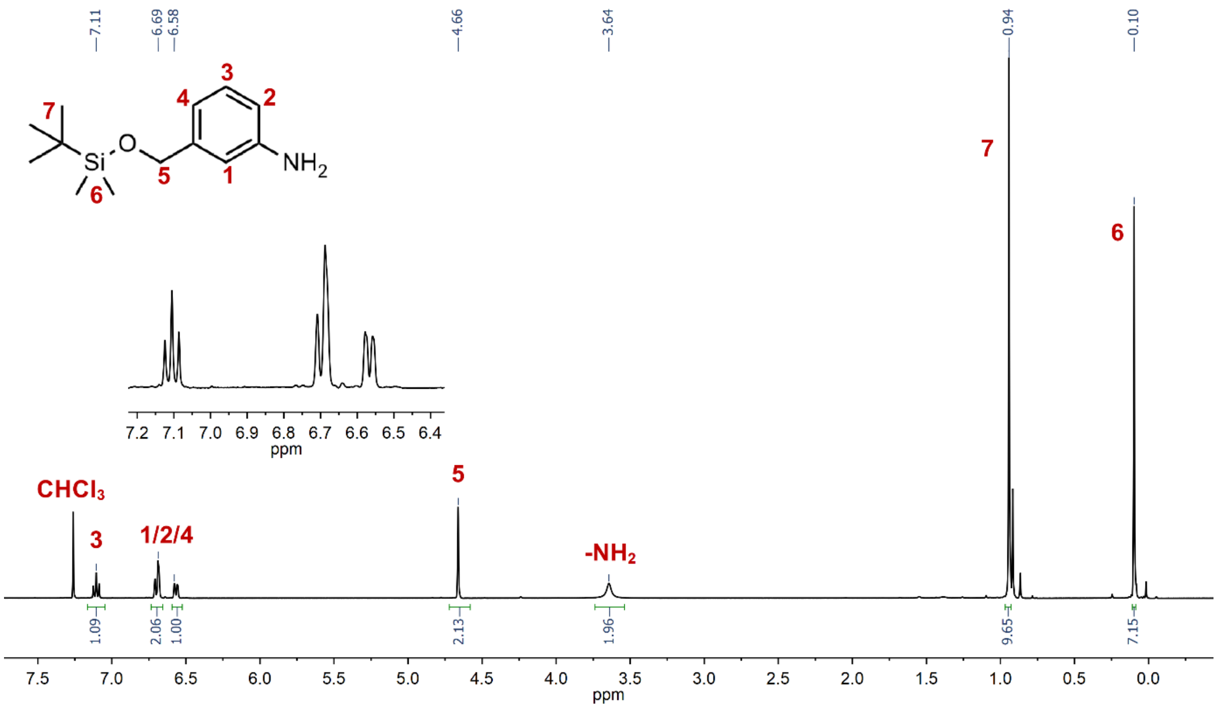

Figure S81: ^{1}H NMR ($CDCl_3$, 400 MHz, 293 K) spectrum of compound (**S7**).

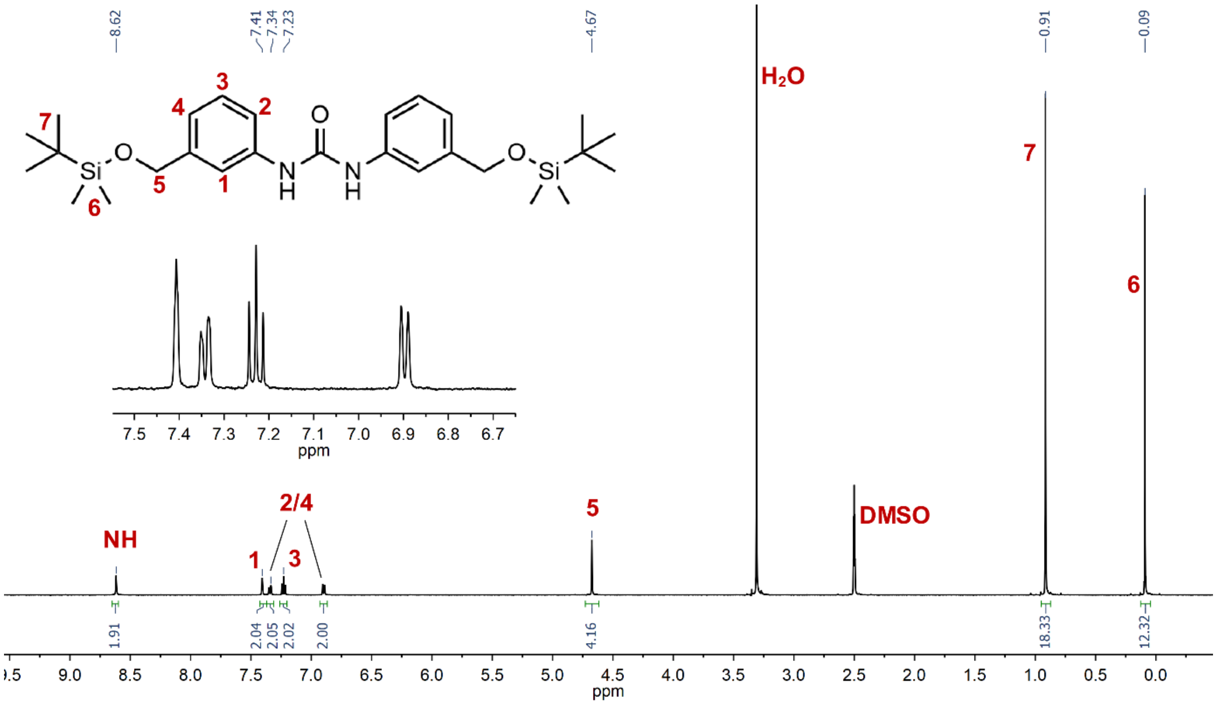

Figure S82: ^{1}H NMR (DMSO-d_6, 400 MHz, 293 K) spectrum of compound (**S8**).

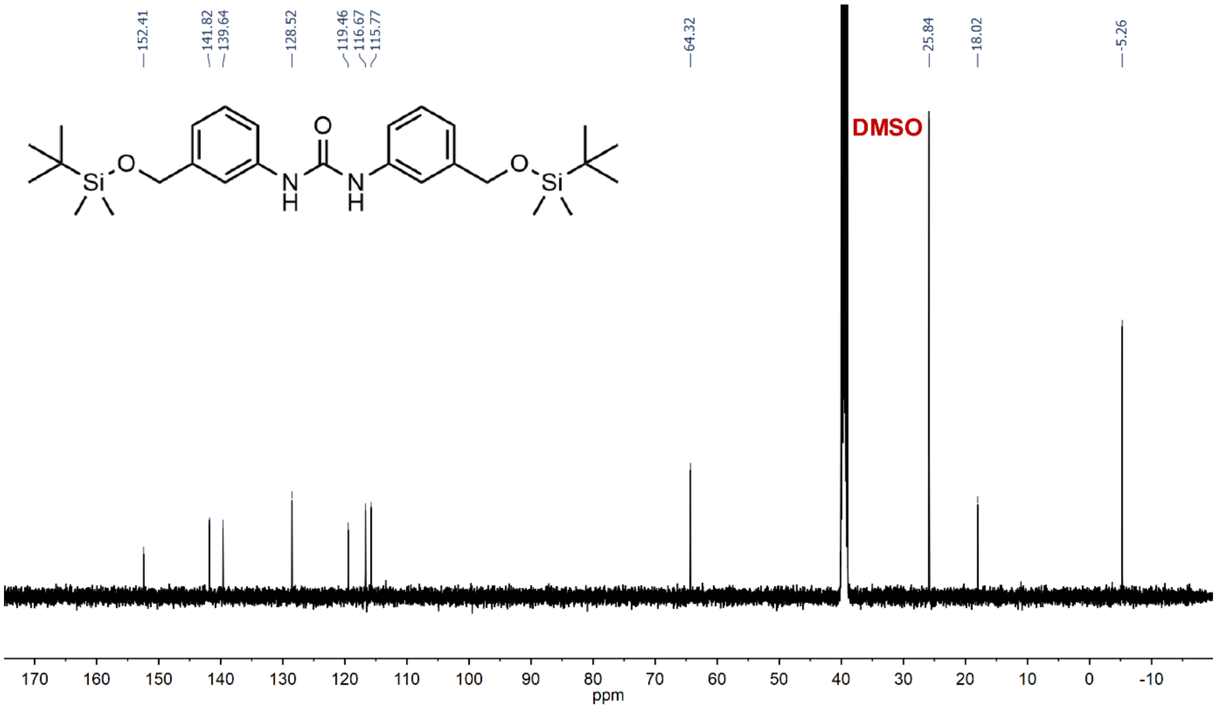

Figure S83: ^{13}C NMR (DMSO-d_6, 101 MHz, 293 K) spectrum of compound (**S8**).

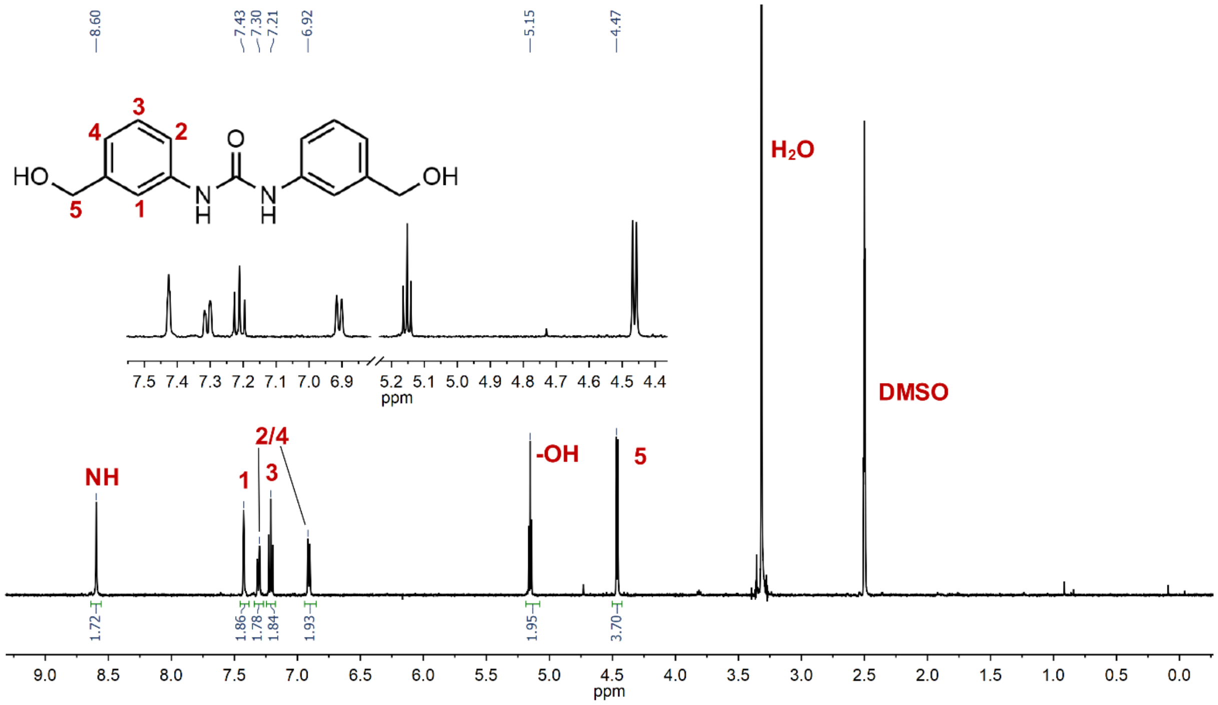

Figure S84: ^{1}H NMR (DMSO-d_6, 400 MHz, 293 K) spectrum of compound (**S9**).

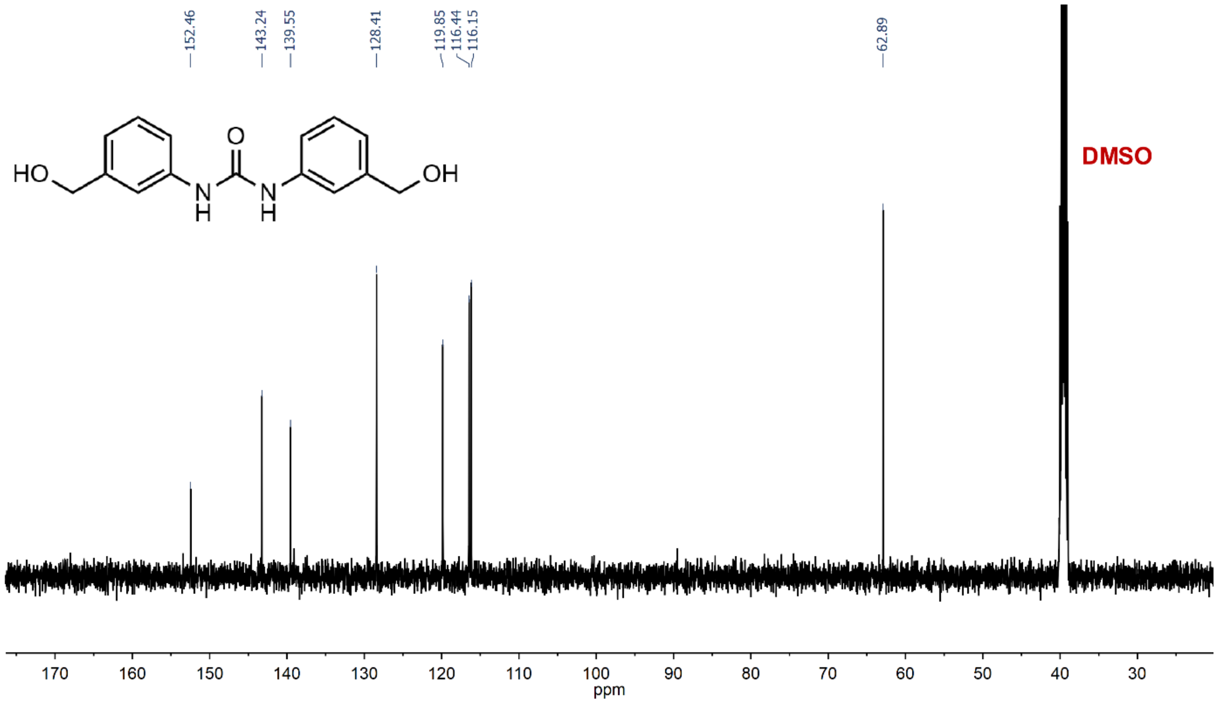

Figure S85: ^{13}C NMR (DMSO-d_6, 101 MHz, 293 K) spectrum of compound (**S9**).

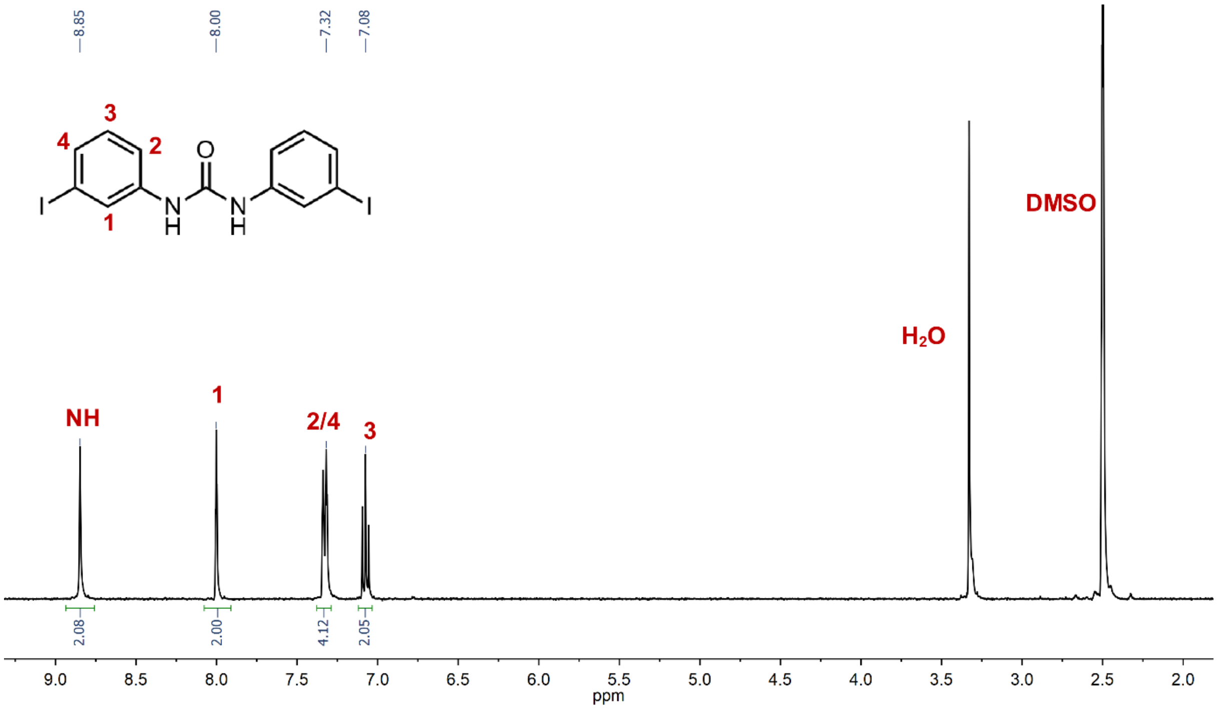

Figure S86: ^{1}H NMR (DMSO-d6, 400 MHz, 293 K) spectrum of compound (**S11**).

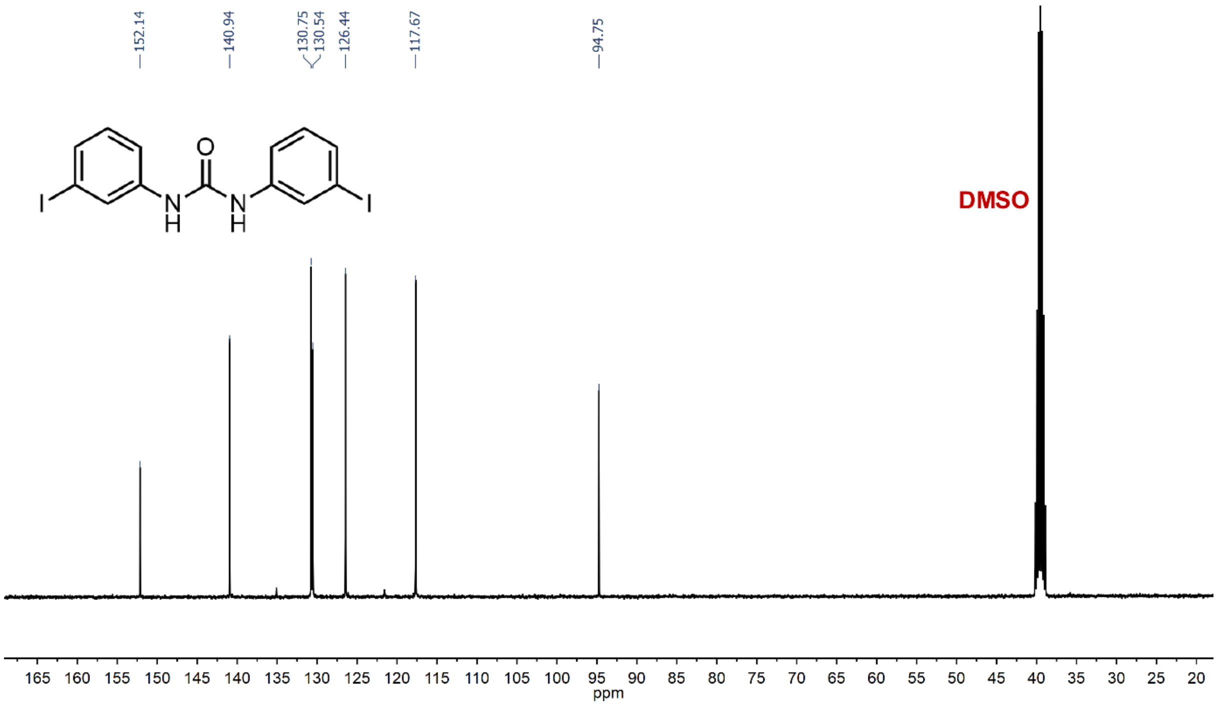

Figure S87: ^{13}C NMR (DMSO-d6, 101 MHz, 293 K) spectrum of compound (**S11**).

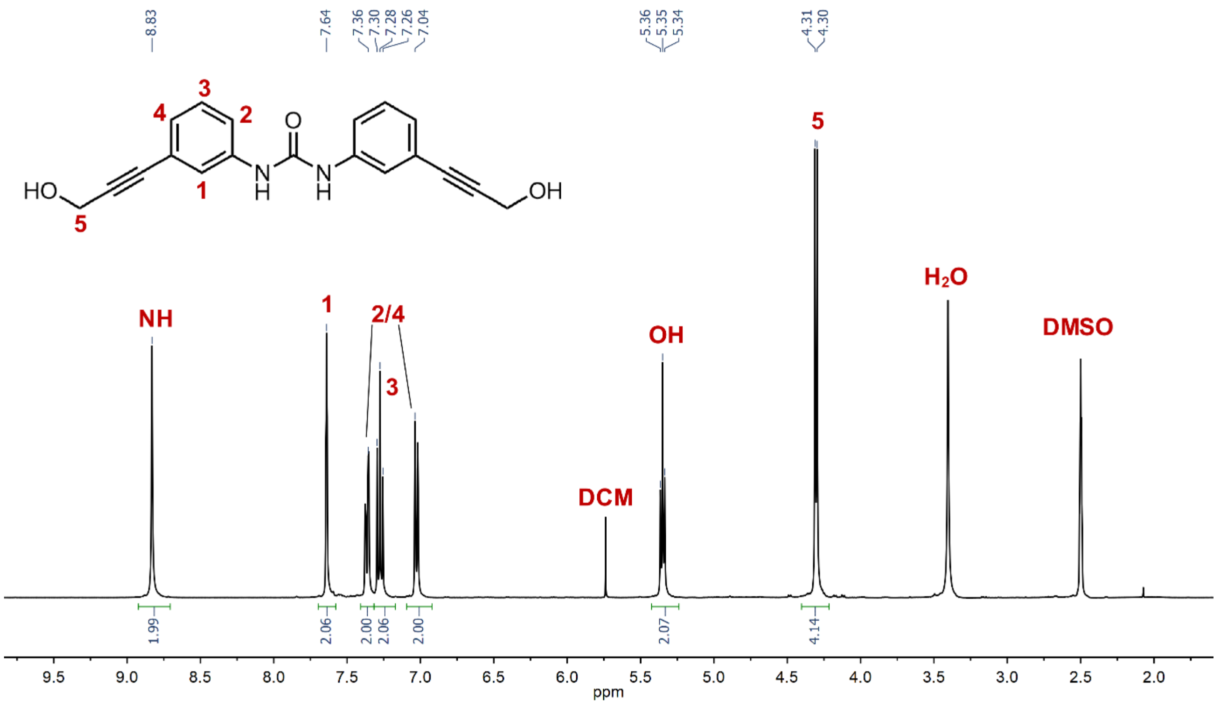

Figure S88: ^{1}H NMR (DMSO-d$_6$, 400 MHz, 293 K) spectrum of compound (**S12**).

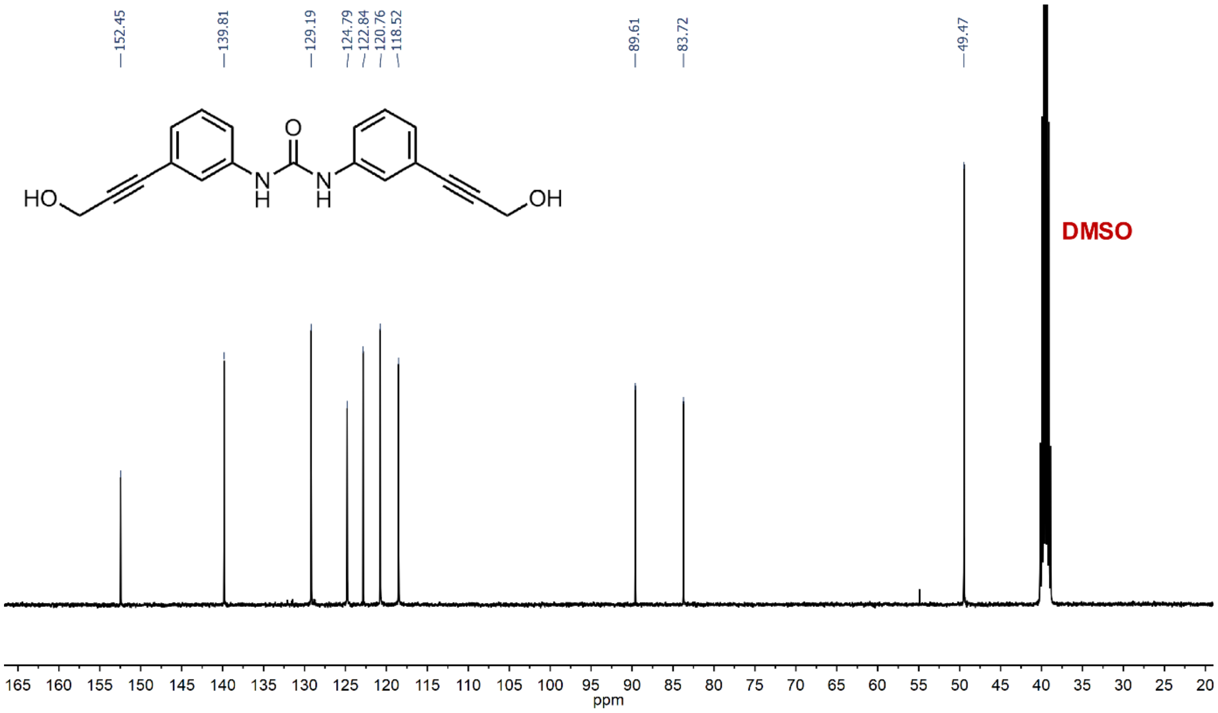

Figure S89: ^{13}C NMR (DMSO-d$_6$, 101 MHz, 293 K) spectrum of compound (**S12**).

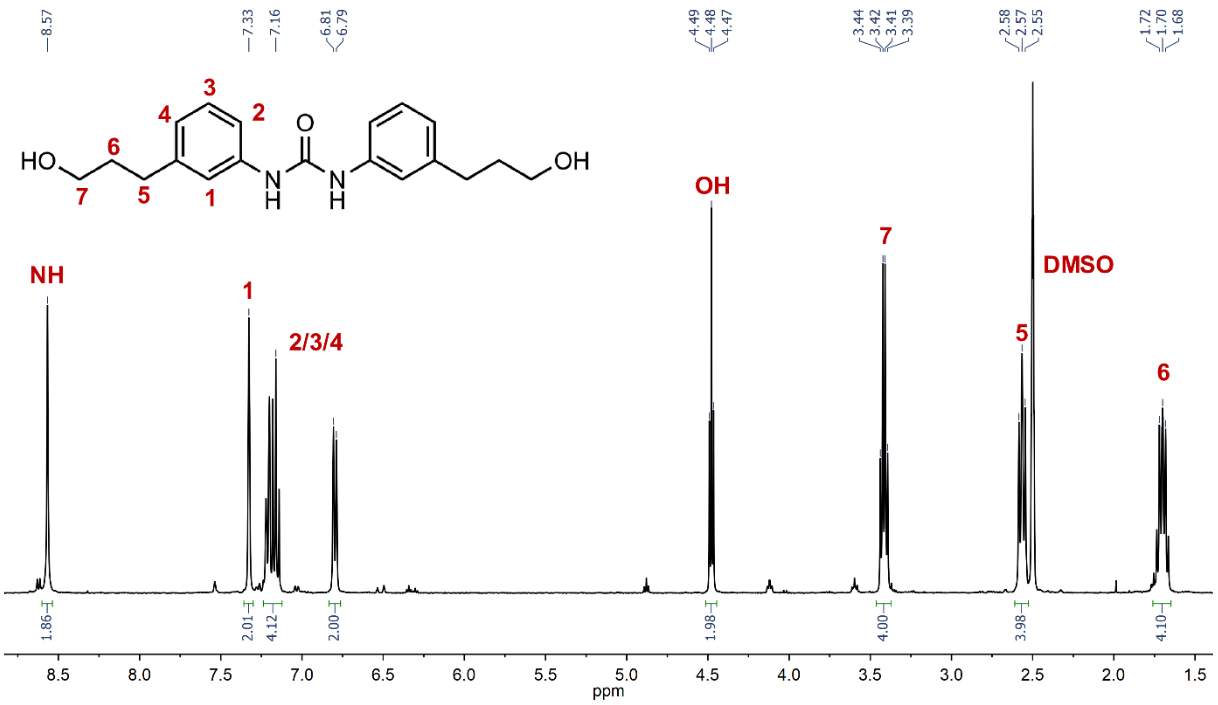

Figure S90: ^{1}H NMR (DMSO-d_6, 400 MHz, 293 K) spectrum of compound (**2**).

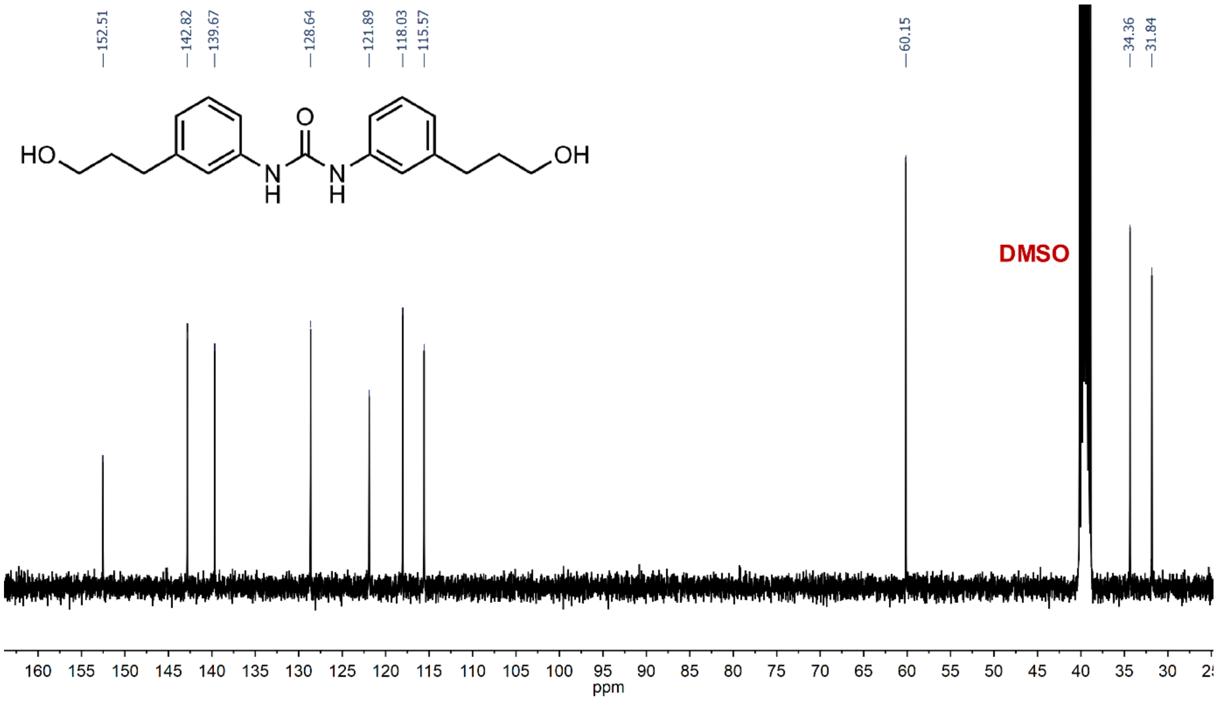

Figure S91: ^{13}C NMR (DMSO-d_6, 101 MHz, 293 K) spectrum of compound (**2**).

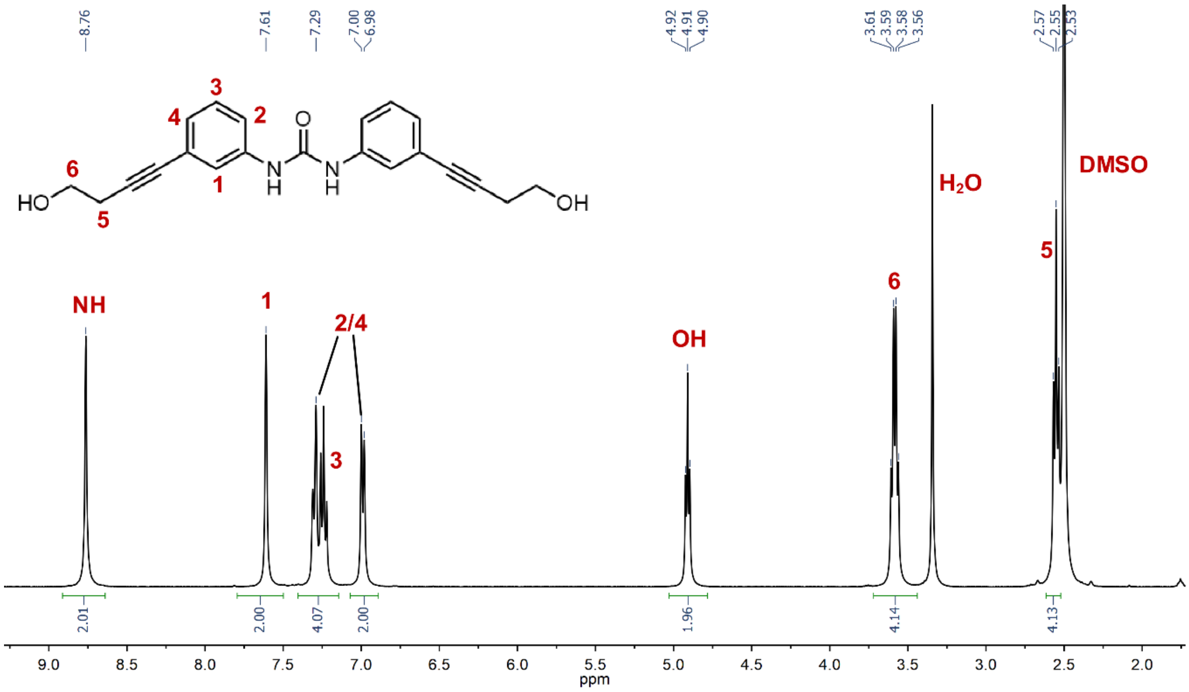

Figure S92: ^{1}H NMR (DMSO-d_6, 400 MHz, 293 K) spectrum of compound (**S13**).

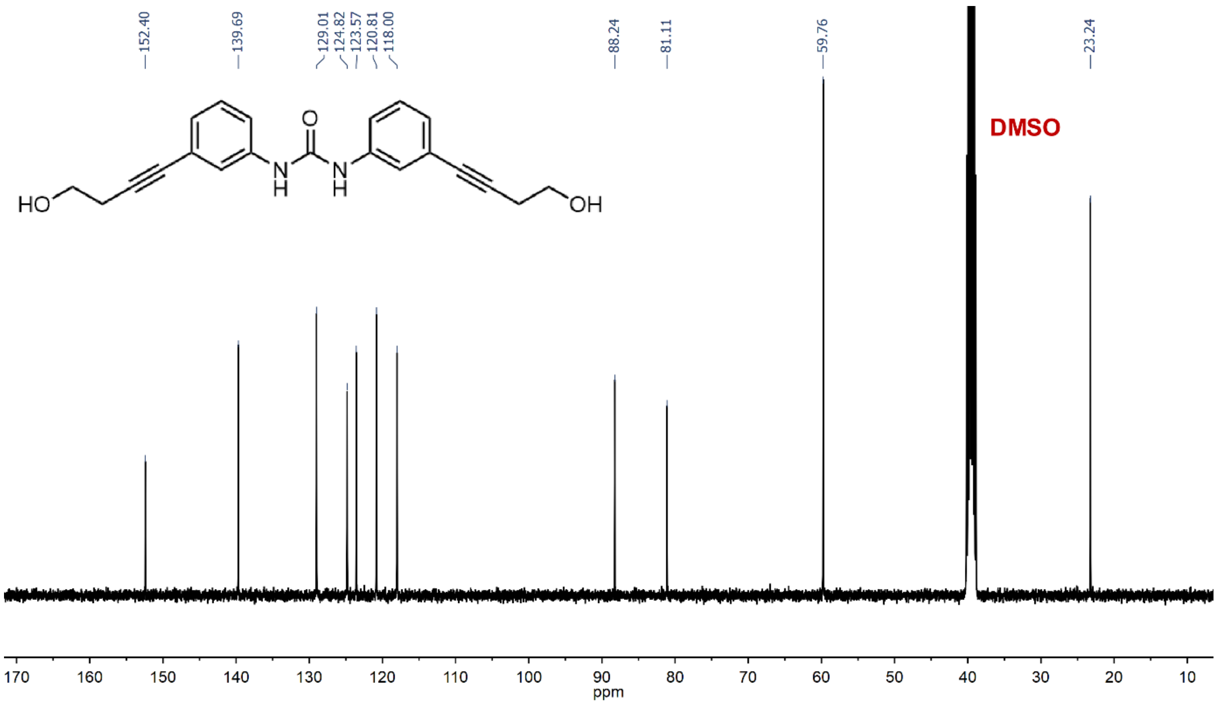

Figure S93: ^{13}C NMR (DMSO-d_6, 101 MHz, 293 K) spectrum of compound (**S13**).

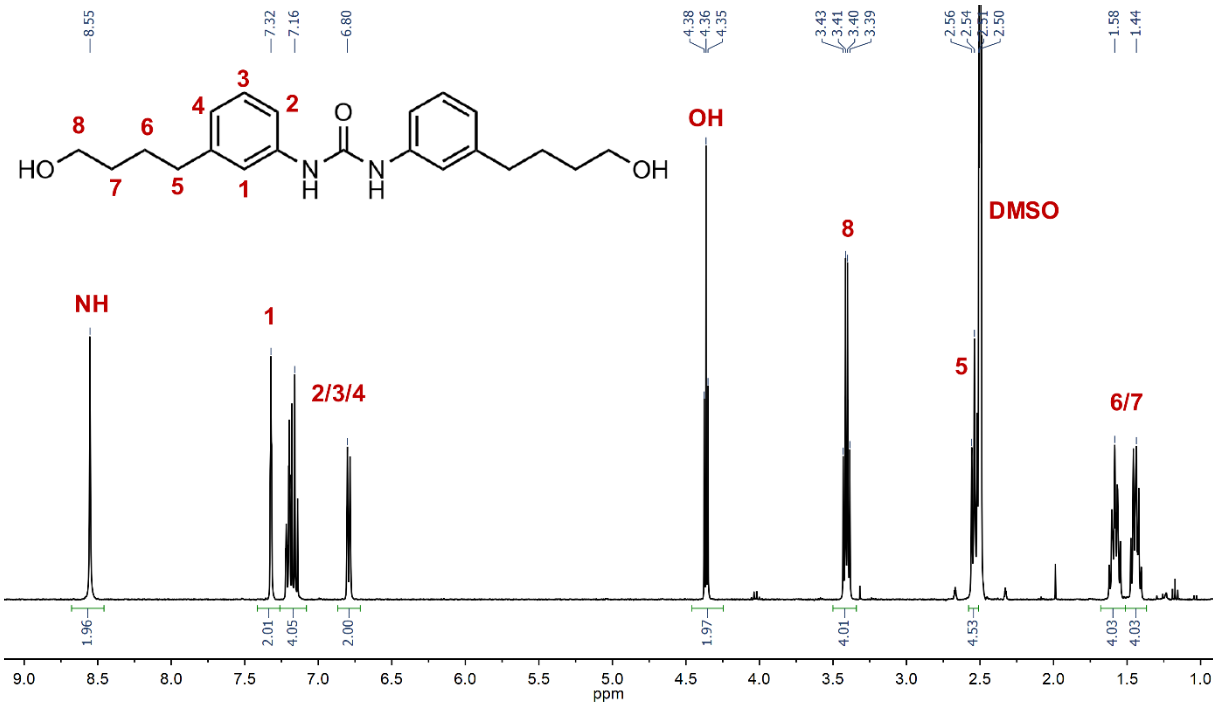

Figure S94: ^{1}H NMR (DMSO-d_6, 400 MHz, 293 K) spectrum of compound (**3**).

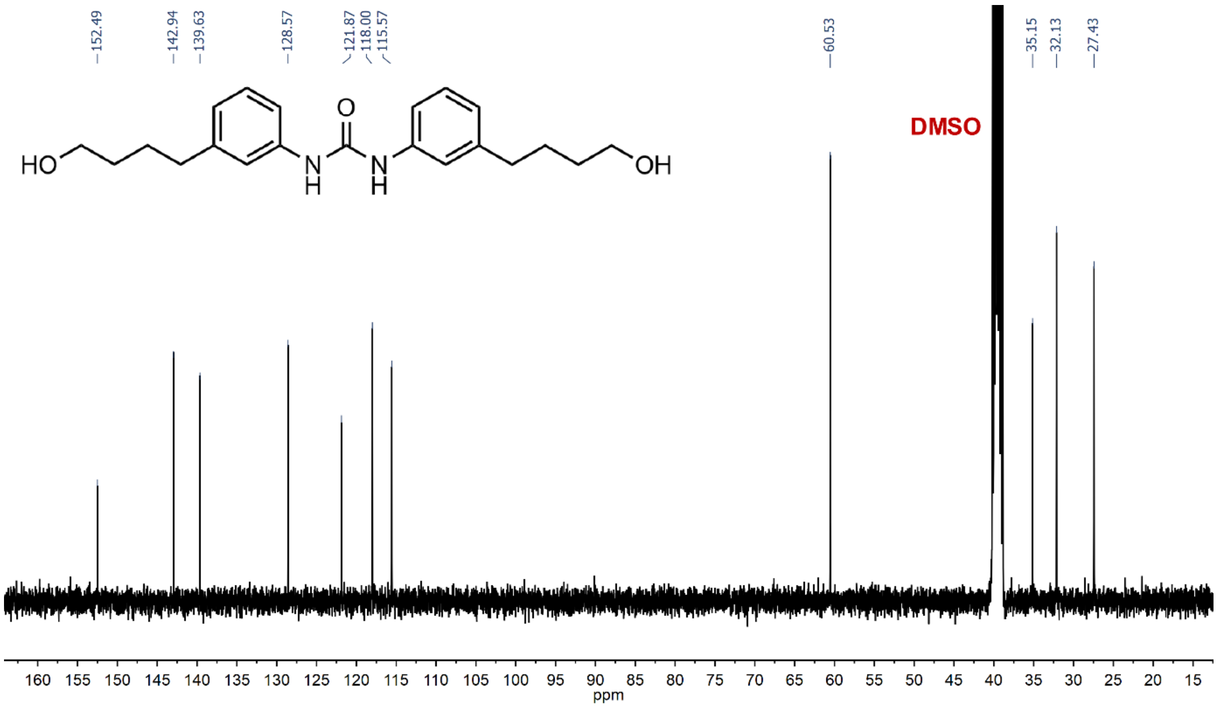

Figure S95: ^{13}C NMR (DMSO-d_6, 101 MHz, 293 K) spectrum of compound (**3**).

References

[1] D. A. Case, H. M. Aktulga, K. Belfon, I. Y. Ben-Shalom, S. R. Brozell, D. S. Cerutti, T. E. Cheatham III, G. A. Cisneros, V. W. D. Cruzeiro, T. A. Darden, R. E. Duke, G. Giambasu, M. K. Gilson, H. Gohlke, A. W. Goetz, R. Harris, S. Izadi, S. A. Izmailov, C. Jin, K. Ka, P. A. Kollman, *Amber 2021*, University of California, San Francisco, **2021**.

[2] J. Wang, R. M. Wolf, J. W. Caldwell, P. A. Kollman, D. A. Case, *J. Comput. Chem.* **2004**, *25*, 1157–1174.

[3] C. I. Bayly, P. Cieplak, W. D. Cornell, P. A. Kollman, *J. Phys. Chem.* **1993**, *97*, 10269–10280.

[4] J. Wang, W. Wang, P. A. Kollman, D. A. Case, *J. Mol. Graph. Model.* **2006**, *25*, 247–260.

[5] T. Fox, P. A. Kollman, *J. Phys. Chem. B* **1998**, *102*, 8070–8079.

[6] P. Li, L. F. Song, K. M. Merz, *J. Chem. Theory Comput.* **2015**, *11*, 1645–1657.

[7] A. Ohtera, Y. Miyamae, K. Yoshida, K. Maejima, T. Akita, A. Kakizuka, K. Irie, S. Masuda, T. Kambe, M. Nagao, *ACS Chem. Biol.* **2015**, *10*, 2794–2804.

[8] N. Umezawa, N. Matsumoto, S. Iwama, N. Kato, T. Higuchi, *Bioorganic Med. Chem.* **2010**, *18*, 6340–6350.

[9] M. J. Thompson, V. Borsenberger, J. C. Louth, K. E. Judd, B. Chen, *J. Med. Chem.* **2009**, *52*, 7503–7511.

[10] P. Thordarson, *Chem. Soc. Rev.* **2011**, *40*, 1305–1323.

[11] D. Brynn Hibbert, P. Thordarson, *Chem. Commun.* **2016**, *52*, 12792–12805.

Author Contributions

S.H. performed all self-assembly experiments, isolated and characterized all products. O.S. made first observation of **[Cl^-⊂*o*-Me_2-ur-C3]PPh_4^+** formation. M.H. and C.M.J. performed all computational chemistry experiments. J.Z. made first observation of **[Cl^-⊂*o*-Me_2-ur-C2][Cs^+⊂*o*-Me_2-2.2.2]** formation**.** T.R. synthesized diol **S9**. O.S. and M.v.D. conceived the project idea. S.H., M.v.D. and C.M.J. wrote the manuscript. M.v.D. directed the project.
